# Two-dimensional liquid chromatography and ion mobility-mass spectrometry for the multicomponent characterization of different parts of the medicinal plant *Gynostemma longipes*

**DOI:** 10.3389/fchem.2023.1203418

**Published:** 2023-09-01

**Authors:** Wei Zheng, Gang Li, Guang Yang, Pengxin Lu, Qi Li, Mengmeng Zhang, Ming Yuan, Xiaojuan Chen, Chenchen Wang, Baolin Guo, Baiping Ma

**Affiliations:** ^1^ Beijing Institute of Radiation Medicine, Beijing, China; ^2^ Waters Technologies Limited, Shanghai, China; ^3^ Shaanxi Cuiyuankang Health Industry Group Co., Ltd., Shaanxi, China; ^4^ Peking Union Medical College, Institute of Medicinal Plant Development, Chinese Academy of Medical Sciences, Beijing, China

**Keywords:** *Gynostemma longipes*, different parts, two-dimensional liquid chromatography, characteristic mass fragments, collision cross-section values

## Abstract

Herba Gynostemma (Jiaogulan) is an herbaceous plant of the genus *Gynostemma* in the family Cucurbitaceae. *Gynostemma longipes* has lipid-lowering activity, thus, it is used as a medicinal material. However, its medicinal using parts have been recorded as whole plants or aerial parts in different provincial quality standards; therefore, it is necessary to conduct a comprehensive compositional analysis of the different parts of *G. longipes* (rhizomes, stems, and leaves) used in traditional medicine. In this study, offline two-dimensional liquid chromatography-ion mobility-quadrupole time-of-flight mass spectrometry (2D-LC/IM-QTOF-MS) was used to analyze the different parts of *G. longipes* obtained from Shaanxi province, China. By combining the retention times, mass fragments, collision cross-section values, reference standards, and information concerning literature compounds, 396 components were identified from the three parts of the plant, including 94 groups of isomers, and 217 components were identified or tentatively identified as new compounds. In the rhizomes, leaves, and stems, 240, 220, and 168 compounds, respectively, were identified. Differential analysis of the compounds in the rhizomes and aerial parts was also carried out, and 36 differential components were identified, of which 32 had higher contents in the rhizomes. Therefore, these findings indicate that the number of chemical components and the content of major differential components are higher in the rhizomes than the leaves and stems of *G. longipes* from the Maobaling Planting Base in Pingli county, Shaanxi province. Thus, the rhizomes of *G. longipes* are also an important part for medicinal use. These results will contribute to the establishment of quality control methods for *G. longipes*.

## 1 Introduction

Herba Gynostemma (Jiaogulan or Jiao-Gu-Lan) is an herbaceous plant in the genus *Gynostemma* and the family Cucurbitaceae, including *G. longipes*. The main active components of *G. longipes* are dammarane-type triterpenoid saponins, named gypenosides, some of which have been identified as ginsenosides. Pharmacological research has revealed that gypenosides are rich in biologically active, having antihyperlipidemic (Yin et al., 2020; Shu et al., 2022), heart-protective (Qiao et al., 2018), hypoglycemic (Pham et al., 2018), anticancer effects (Zu et al., 2020), immunomodulatory, neuroprotective, anti-inflammatory, and hepatoprotective activities (Nguyen et al., 2021). Consequently, many preparations and health products have been developed based on *G. longipes*, for example, Jiaogulan Zonggan tablets, Jiaogulan oral liquid, and gynostemma tea (Yang et al., 2008), and these are widely used in China (Huang et al., 2022). However, Jiaogulan is not recorded in the 2020 edition of the Chinese Pharmacopoeia. And the medicinal using part of Herba Gynostemma was not unique in existing provincial quality standards, such as Shaanxi, Sichuan.

To date, many studies have focused on the differences in the chemical composition of Herba Gynostemma obtained from different geographical locations. For example, 199 saponins of *G. pentaphyllum* from different habitats were identified using the ComMS^n^DB post-processing strategy; however, this method only focused on mass fragments obtained in negative-ion mode, and the discrimination of isomers was ignored (Wang et al., 2020). A few researchers have analyzed the different plant parts of *G. longipes* and showed that a greater variety of saponins are present in the underground parts. In addition, the gypenosides from the underground parts have good lipid-lowering activity (Teng et al., 2022). Therefore, it is necessary to analyze and compare the chemical compounds in different parts of this medicinal plant systematically to enhance quality control.

In recent years, as a result of its efficient separation, high sensitivity, and good resolution, ultra-high performance liquid chromatography-quadrupole time-of-flight mass spectrometry (UHPLC-QTOF/MS) has become a powerful tool to profile and identify the chemical compounds in natural products. Based on MS databases of the components of natural products, new compounds can be rapidly identified by comparing the mass spectra of unknown components with those of known references (Zheng et al., 2017). However, single-dimensional chromatographic separation often has limited peak capacity, which limits characterization. In contrast, two-dimensional liquid chromatography connects two columns having different separation mechanisms, thereby increasing separation ability and peak capacity. Moreover, the use of offline mode increases the content of trace components through enrichment (Zhou et al., 2020; Wang et al., 2022). Further, natural products usually contain hundreds of chemical components; thus, isomerism is common. It is difficult to distinguish a large number of isomers using chromatographic separation and mass fragmentation. However, ion mobility-mass spectrometry (IMS) can separate compounds depending on the charge, size, and shape of the gas-phase ions, thus providing an additional separation dimension. This technique also provides a compound collision cross-section (CCS). Thus, the reliable identification of metabolites (Zhang et al., 2019) and distinction of isomers (Shi et al., 2018) based on the combination of retention times, mass fragments, and CCS values has become possible. Zhou et al. (2017) measured 953 CCS values of 617 metabolites, and Vasilopoulou et al. (2020) measured the CCS values of 1,856 lipids to establish an experimental CCS value database for characterizing. However, because of the lack of reference standards, few studies have been conducted on the experimental CCS values of natural products; only 146 components have been measured by trapped ion mobility spectrometry (TIMS)-MS (Schroeder et al., 2019). Using computational chemistry and machine learning methods, predicted CCS databases for compound prediction have been developed, including MOBCAL, DarkChem, ALLCCS, and CCSondemand (Zhou et al., 2020; Broeckling et al., 2021; Connolly et al., 2021). For example, Song et al. (2022) determined 1,038 experimental CCS values of 675 reference standards using Vion IMS-QTOF and compared the predicted CCS values from three CCS prediction tools: ALLCCS, CCSbase, and CCSondemand, and it was found that CCSondemand contains the most accurate predictions: 57.6% of the predicted values for [M + H]^+^ and 56.3% of the predicted values for [M–H]^−^ were within an error threshold of 2% (acceptable error). Although there were deviations between the experimental and predicted values, the predicted CCS values provide a reference for tentative identification and false-positive elimination. Furthermore, Wang et al. (2022) distinguished 12 pairs of isomers using the CCS prediction values without reference standards.

The goal of this work was to analyze the chemical components of different parts (rhizomes, stems, and leaves) of *G. longipes* grown in Shaanxi province, China. First, offline two-dimensional liquid chromatography-ion mobility-quadrupole time-of-flight mass spectrometry (2D-LC/IM-QTOF-MS) was used to analyze the different parts of *G. longipes*. Secondly, the characteristic aglycone fragments and neutral-loss fragments were summarized from the reference standards in both positive- and negative-ion modes using UHPLC-Q-TOF/MS. Thirdly, for rapid identification, a total component mass spectrometry database of Herba Gynostemma was established through literature research, including 501 components, such as triterpenoid saponins, flavonoids, organic acids, and sterols. To discriminate the isomers, experimental CCS values for [M + HCOO]^−^ were measured, and the applicability of the predicted CCS values for distinguishing isomers was verified by comparison with measured CCS values for 100 reference standards. A total of 396 components were identified from the three parts of *G. longipes*, including 94 groups of isomers, and 217 components were identified or tentatively identified as new compounds. Finally, hierarchical cluster analysis (HCA), principal component analysis (PCA), and orthogonal partial least squares discriminant analysis (OPLS-DA) were used to reveal the differences between the components in the different parts of *G. longipes*. The potential differential components were selected based on the variable importance in the projection (VIP) value and processed using a heatmap to observe the distributions intuitively.

## 2 Materials and methods

### 2.1 Materials and reagents

Acetonitrile and formic acid (MS-grade) were purchased from Fisher Scientific (Loughborough, United Kingdom). Leucine-enkephalin and Major Mix calibration fluid were purchased from Waters Corp. (Milford, MA, United States), and distilled water was purchased from Watson (Guangzhou, China). Other analytical-grade reagents were purchased commercially (Beijing, China).

One hundred gypenoside reference standards (purity >95%) were previously isolated in our laboratory, and their structures were confirmed by MS and NMR (some of which were literature data) (Li et al., 2021; Zhang et al., 2021) (Supplementary Figures S1 and S2; Supplementary Table S1).

Forty-four batches of *G. longipes* were collected from the Maobaling Planting Base in Pingli county, Shaanxi province, China, and identified by Prof. Baolin Guo of the Institute of Medicinal Plant Development, Chinese Academy of Medical Sciences, and Peking Union Medical College, Beijing, China. In total, 15 rhizome samples had been grown within a year, labeled R1–R15; the 15 stem samples were all uniform (20 cm removed from the top and cut down to 30 cm in length) from the longest branch in the plant, labeled S1–S15; and 14 leaves from same branches, labeled L1–L14.

### 2.2 Sample and standard solution preparation

Stock solutions of the 100 reference standards were prepared in methanol at a concentration of 0.3 mg/mL and pipetted in the same amount, and 14 mixed reference solutions were prepared according to the grouping information in Supplementary Table S2. All the solutions were stored at 4 °C for further study.

Forty-four batches of samples were crushed evenly until they passed through a 40-mesh sieve. Then, a fine powder sample was accurately weighed to 0.25 g and added to 10 mL of 70% aqueous methanol. The conical flask was tightly plugged, shaken, and weighed. After sonication for 30 min, the sample was cooled to room temperature, and the weight loss was compensated for using extraction solution. All the solutions were filtered through a 0.22-μm filter membrane before PCA analysis. One milliliter of the supernatant solution from each batch of samples was blended for use as a quality control sample (QC) and applied between every six injections during the UHPLC-Q-TOF-MS analysis to monitor system stability.

Simultaneously, 1 mL of the supernatant solution was mixed into different parts to prepare the QC samples for each plant part, labeled QC-R, QC-S, and QC-L. Then, QC-R, QC-S, and QC-L were concentrated at reduced pressure to dryness (distillation temperature ≤45 °C), dissolved in 1 mL of 70% methanol, and passed through a 0.22-μm filter membrane before one-dimensional analysis.

### 2.3 Offline 2D-LC/IM-QTOF-MS

One-dimensional hydrophilic chromatography (1D HILIC) and two-dimensional reverse-phase chromatography (2D RPC) were combined to establish an offline 2D-LC system for separating the chemical compounds in *G. longipes*. 1D HILIC was performed using a Vanquish Core system (Thermo Fisher Scientific, Germany) equipped with a dual pump, autosampler, column compartment, charged aerosol detector (CAD), and diode array detector (DAD). A YMC-Triart Diol-HILIC column (4.6 mm × 250 mm, 5 μm) was used at a column temperature of 40 °C. The mobile phase comprised water with 0.2% formic acid (A) and acetonitrile (B). The gradient protocol used was as follows: 0–5.0 min, 95%–90% B; 5.0–20.0 min, 90%–77% B; 20.0–25.0 min, 77%–70% B; 25.0–30.0 min; 70%–55% B; 30.0–32.0 min, 55% B; 32.0–33.0 min, 55%–95% B; and 33.0–40.0 min, 95% B. The flow rate was 1 mL/min, the sample injection volume was 5 μL, and the experiment was repeated 20 times. CAD was used to guide separation, the evaporator temperature was set to 35 °C, and the nitrogen gas pressure for the nebulizer was set at 0.6 MPa. The data collection rate was set at 5 Hz using a filter constant of 5 s. The DAD was used to monitor the ultraviolet signals at 254 and 203 nm to guide fractionation. The sub-fractions were collected via valve switching: fraction 1: 0–6 min, fraction 2: 6–8.9 min, fraction 3: 8.9–11.6 min, fraction 4: 11.6–15.2 min, fraction 5: 15.2–19 min, fraction 6: 19–26.8 min, and fraction 7: 26.8–48 min. When combining the same fractions, the distillation temperature was maintained under 45 °C, and the samples were dried to remove the solvent at reduced pressure. The seven fractions were sequentially dissolved in 1 mL of 70% methanol and passed through a 0.22-μm filter membrane before two-dimensional analysis.

The 2D RPC measurements were performed using a Waters ACQUITY I-Class system (Waters Corp., Milford, MA, USA). A Waters ACQUITY UPLC CSH C18 column (2.1 mm × 100, 1.7 μm) was used at a column temperature of 40 °C. The linear mobile phase was water containing 0.1% formic acid water(A) and 0.1% formic acid acetonitrile (B). The gradient protocol was as follows: B was started at 10%, increased to 25% at 2 min, 32% at 7 min, 33% at 10 min, 35% at 12 min, 46% at 22 min, 56% at 27 min, and finally increased to 95% at 30 min. The flow rate was 0.5 mL/min. The sample injection volume was 1 μL.

A VION IMS-QTOF system (Waters Corp., Wilmslow, United Kingdom) was used to acquire the HDMS^E^ data in both negative- and positive-ion modes, and the data were acquired from 50 to 2,000 Da. The electrospray ionization (ESI) source temperature was set to 110 °C, the desolvation gas was nitrogen (flow rate: 850 L/h), and the temperature was 450 °C. The capillary voltages were 3 kV (positive-ion mode, ESI^+^) and 2.5 kV (negative-ion mode, ESI^−^), and the cone voltage was 50 V. In a low-collision energy (CE) scan, the CE was 4 eV; in a high-CE scan, the ramp CEs were 50–70 and 15–25 eV in negative- and positive-ion modes, respectively. Leucine-enkephalin was used as the lock mass at a flow rate of 10 μL/min for calibration, whereas Major Mix was used to calibrate the CCS values. The instrument was controlled using UNIFI 1.9.4 (Waters Corp., Milford, MA, USA).

### 2.4 CCS measurement and prediction

Major Mix calibration kit was used for CCS calibration and was injected at the beginning of the sequence and between every six injections to monitor the variation in the measured CCS values and ensure that it was less than 2%. One hundred reference standards were detected using HDMS^E^ data acquisition mode, to measure the CCS values for [M + H]^+^, [M + Na]^+^, [M + NH_4_]^+^ in positive-ion mode, and [M−H]^−^ and [M + HCOO]^−^ in negative-ion mode. Each compound was measured three times, and the average value was obtained as the CCS value of the compound. CCSondemand (https://ccs.on-demand.waters.com/) was used to predict the CCS values of the gypenosides in the “Herba Gynostemma mass spectrometry database” (Zhou et al., 2020).

### 2.5 Data analysis using the UNIFI informatics platform

The analysis method was as follows. The maximum allowed number for peak detection was 10,000 for 2D peak detection, and the peak intensity threshold was 100 counts for high-energy and 1,000 counts for low-energy 4D peak detection. Mass and fragment errors were both set at 10 mDa for compound identification. Adducts included + HCOO^−^, −H and +H, +Na, and +NH_4_ in negative- and positive-ion modes. Leucine-enkephalin was used to ensure mass accuracy, for which [M−H]^−^ 554.2620 and [M + H]^+^ 556.2766 were used in negative- and positive-ion modes, respectively. The “Common Fragment Settings” and “Common Neutral-Loss Settings” were based on the fragments of gypenosides in the mass spectrum.

### 2.6 Multivariate statistical analysis

Forty-four batches of sample data were reprocessed through deconvolution, alignment, and data reduction to provide a list of mass and retention time (*Rt*) pairs, along with the corresponding peak areas for all the detected peaks from each file in the dataset. The processed data list was then imported into SIMCA 14.1 (Sartorius Stedim, Germany) for PCA, HCA, and OPLS-DA analyses. The parameters used in the analysis were 0–30 min for the range of *Rt*, 50–2,000 Da for the mass range, 0.02 Da for the mass tolerance, and 0.10 min for *Rt* tolerance. All test groups were discriminated using PCA and HCA to determine whether the different groups could be separated. Then, OPLS-DA was carried out to discriminate the ions that contributed most to the classification of the samples, and a VIP-plot was constructed to show the variables that contributed to the classification. Heatmap visualization was performed using TBtools to show changes in the variable marker content (Chen et al., 2020).

## 3 Results and discussion

### 3.1 Establishment of the offline 2D-LC/IM-QTOF-MS method

**FIGURE 1:**
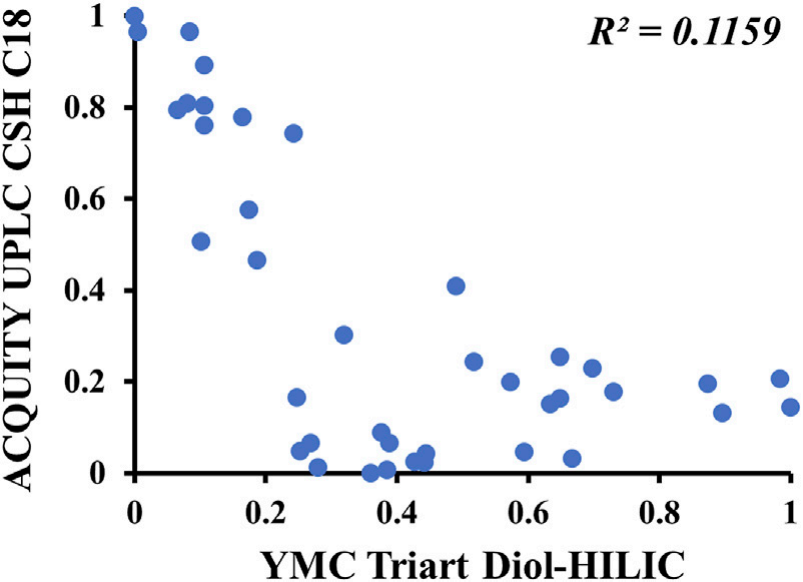
Scatter plots of the separation differences between one-dimensional HILIC column (horizontal axis) and two-dimensional C18 column (vertical axis) for 39 reference standards. X-axis: the relative retention time of 39 reference standards separated on HILIC column. Y-axis: the relative retention time of 39 reference standards separated on C18 column.

Two-dimensional liquid chromatography significantly improves the separation ability and peak capacity of ions and is helpful for the total component analysis of natural products. Reverse-phase chromatography (RPLC) is suitable for the separation of saponins, and HILIC and RPLC show good orthogonality. Therefore, an offline 2D-LC condition was established by optimizing the 1D method in the HILIC column and the 2D method in the RP-C18 column. The gypenosides are the main components of *G. longipes*; therefore, six RP-C18 columns were used to evaluate the separation ability for the gypenosides of *G. longipes*, including a Waters ACQUITY UPLC BEH C18 column (2.1 mm × 100, 1.7 μm), ACQUITY UPLC HSS C18 column (2.1 mm × 100, 1.8 μm), ACQUITY UPLC CSH C18 column (2.1 mm × 100, 1.7 μm), ACQUITY UPLC HSS T3 column (2.1 mm × 100, 1.8 μm), ACQUITY UPLC BEH Shield RP18 column (2.1 mm × 100, 1.7 μm), and Phenomenex Kinetex XB-C18 column (2.1 mm × 100, 1.7 μm). Comparing the separation ability and chromatographic capacity, the CSH C18 column was the most suitable for *G. longipes*. At the same time, the gradient elution conditions, mobile phases (water–acetonitrile; 0.1% formic acid in water–acetonitrile, 0.1% formic acid in water–0.1% formic acid in acetonitrile, 10 mM ammonium formate in water–acetonitrile, and 10 mM ammonium formate in water–0.1% formic acid in acetonitrile), and column temperature (25, 30, 35, 40, and 45 °C) were also optimized to achieve better peak shape and separation. Four HILIC columns were used to evaluate the orthogonality between the 1D and 2D methods by calculating the linear regression correlation coefficient (*R* ^2^) (Qiu et al., 2015; Yang et al., 2016) of the relative retention times of the 1D and 2D separations for 39 reference standards (numbered as GL1-21 and JGL1-20 in Supplementary Table S1) of *G. longipes*. As shown in Supplementary Figure S3, the separation performance of the four HILIC and CSH C18 columns was similar. Of the obtained values, the *R* ^2^ value (*R* ^2^ = 0.1159) of the YMC-Triart Diol-HILIC column (Figure 1) was the lowest when using a mobile phase composed of water with 0.2% formic acid (A) and acetonitrile (B). Finally, the YMC-Triart Diol-HILIC column having a column temperature of 40 °C with 0.2% formic acid water–acetonitrile and the CSH C18 column having a column temperature of 40 °C with 0.1% formic acid water–0.1% formic acid acetonitrile was selected as optimum conditions for the 1D and 2D methods, respectively.

To obtain a better MS response and richer mass fragments, different capillary voltages (1, 1.5, 2, 2.5, 3, and 3.5 kV), ESI source temperatures (100, 110, 120, 130, 140, and 150°C), desolvation gas temperatures (350, 400, 450, and 500°C), and CEs in both negative- and positive-ion modes were optimized. Using the chromatographic peak area as an index, nine reference standards (JGL-19, JGL-6, JGL-4, GL-11, GL-20, GL-7, GL-21, GL-14, and GL-15) were selected to evaluate the responses of the gypenosides. Each parameter was determined in triplicate for reliability. The responses of the main gypenosides were the best when the source temperature was 110°C, desolvation gas temperature was 450 °C, and capillary voltages were 2.5 and 3 kV in negative- and positive-ion mode, respectively. The CE was found to have a significant impact on the mass fragments. Based on fragment richness, CEs of 10–80 eV were investigated sequentially in 10-eV steps in negative-ion mode. However, triterpene saponins are prone to fragmentation in positive-ion mode; thus, the CE was investigated as 5–40 eV in ESI^+^ in 5-eV intervals. Finally, the ramp CE was set to 50–70 eV in negative-ion mode but 15–25 eV in positive-ion mode.

### 3.2 Identification of the fragmentation patterns of reference standards

Studying the mass fragments of reference standards in both negative- and positive-ion modes and summarizing the fragmentation patterns is helpful for identifying unknown components. Thirty-nine reference standards for triterpene saponins isolated from *G. longipes* (numbered as GL1-21 and JGL1-20 in Supplementary Table S1) were classified according to the structure of the C17 branched chain, and components with representative structures were selected to study their mass fragments.

**FIGURE 2:**
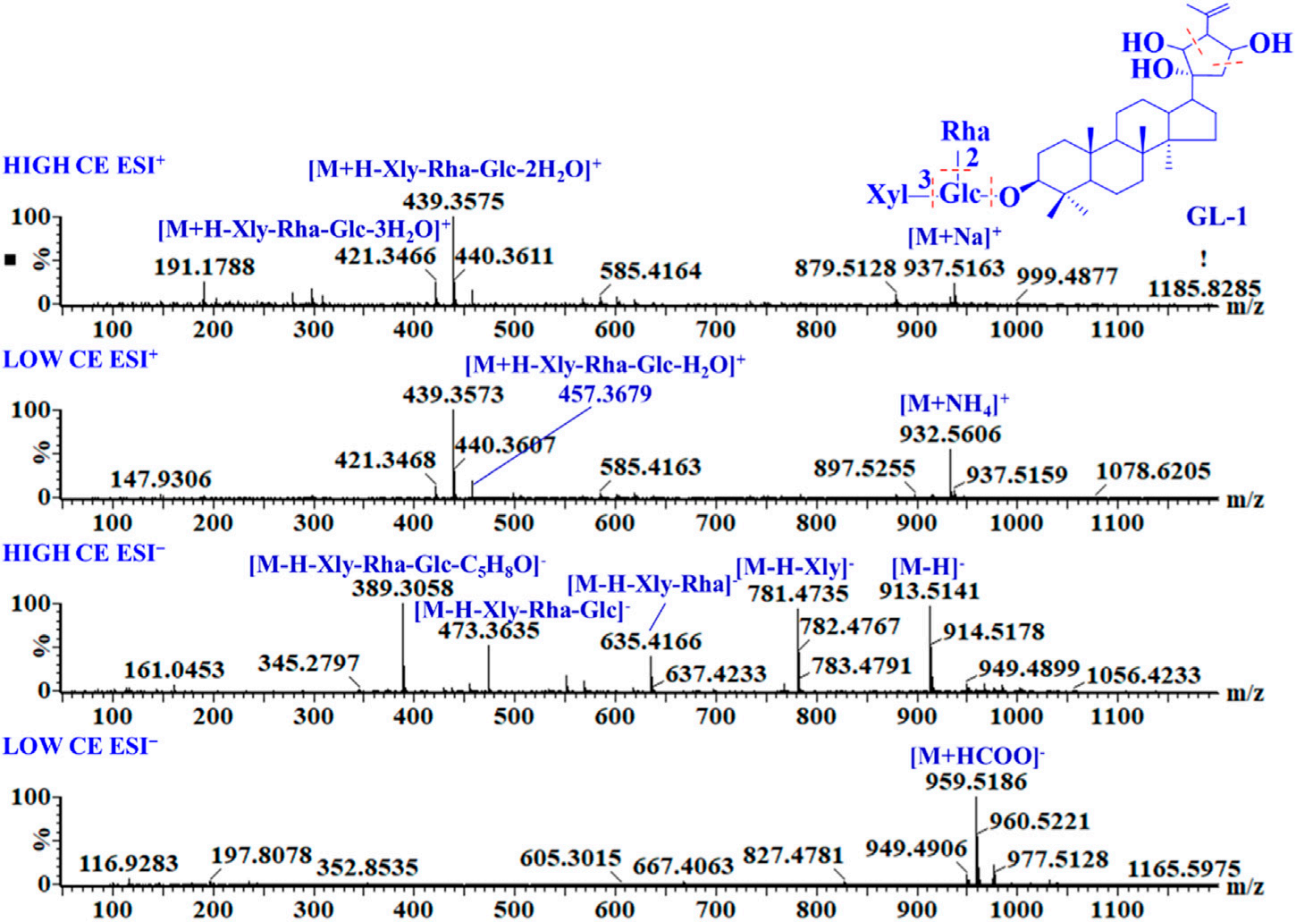
MS and MS^E^ spectra of GL-1.

GL-1 is a triterpene saponin containing a type-A aglycone. In ESI^−^ mode, it produced an adduct ion at *m/z* 959.5186 [M + HCOO]^−^ at low CE and a deprotonated molecular ion at *m/z* 913.5141 [M−H]^−^, as well as a series of glycosyl cleavage fragment ions at *m/z* 781.4735 [M−H−Xyl]^−^, 635.4166 [M−H−Xyl−Rha]^−^, 473.3635 [M−H−Xyl−Rha−Glc]^−^, and 389.3058 [M−H−Xyl−Rha−Glc−C_5_H_8_O]^−^ at high CE. In ESI^+^, the main fragment ions of GL-1 were *m/z* 937.5163 [M + Na]^+^, 932.5606 [M + NH_4_]^+^, 457.3679 [M + H−Xly−Rha−Glc−H_2_O]^+^, 439.3575 [M + H−Xly−Rha−Glc−2H_2_O]^+^, and 421.3466 [M + H−Xly−Rha−Glc−3H_2_O]^+^. Based on the mass spectra (Figure 2), the [M + HCOO]^−^ adduct ions were the main ion fragments in low-CE ESI^−^. In high-CE ESI^−^, the deprotonated precursor ion was detected, and a xylose, rhamnose, and glucose unit connected at C-3 were successively removed from the outside to the inside to obtain the aglycone ion. The cyclic side chain of C-17 was broken and formed a neutral-loss fragment at *m/z* 84.0575. In positive-ion mode, adduct ions of [M + Na]^+^ and [M + NH_4_]^+^ were obtained. Even at a low CE, aglycone fragments and a series of dehydrated ions were detected, but the response of glycosyl cleavage fragments was relatively low.

Aglycones B and C are gypenosides whose C-17 side chains were converted into five-membered oxygen-containing heterocyclic structures. The aglycone of JGL-4 belongs to type B. As shown in Supplementary Figure S4A, the cyclic side chain of C-17 was broken and formed a neutral-loss fragment at *m/z* 100.0524 (C_5_H_8_O_2_) because of a hydroxyl group substituted at C-26 in high-CE ESI^−^. In positive-ion mode, the characteristic aglycone ions were at *m/z* 487, 469, 451, and 433, and the fragment in which the side chain of C-17 was completely cleaved was observed at *m/z* 313 Da. In ESI^−^, GL-14 (the type-C aglycone) produced an adduct ion at *m/z* 999 [M + HCOO]^−^. Owing to an acetyl group connected to glucose, it initially formed a neutral-loss fragment of 42 Da. The glycosyls were then cleaved to the aglycone ion at *m/z* 471, and the side chain of C-17 was broken and formed a neutral-loss fragment at *m/z* 112.0524 (C_6_H_8_O_2_). In ESI^+^, a series of aglycone ions were observed at *m/z* 455, 437, and 419 (Supplementary Figure S4B).

Aglycones of D and E were analyzed using the same method: in negative-ion mode, the aglycone ion was observed at *m/z* 475 for both D and E, whereas in positive-ion mode, the aglycone ions were observed at *m/z* 459, 441, and 423 for D and *m/z* 441, 423, and 405 for E.

The F-aglycone is a gypenoside with a straight chain on C-17. As shown in Supplementary Figure S4C, GL-7 produced an adduct ion at *m/z* 1107 [M + HCOO]^−^ and a doubly charged ion at *m/z* 576 at a low CE in ESI^−^. In high-CE ESI^−^, the fragment ions were observed at *m/z* 929, 783, 621, 459, and 375, attributed to the loss of one xylose, one rhamnose, two glucose residues, a formula of C_6_H_12_, and a neutral-loss fragment of 84.0939 Da because of the side-chain cleavage of C-17. In ESI^+^, GL-7 produced adduct ions at *m/z* 1085 [M + Na]^+^ and 1080 [M + NH_4_]^+^ and a series of aglycone ions at *m/z* 443, 425, and 407. Owing to the different substituents on C-10 and the side chain of C-17, the neutral-loss fragment and aglycone ion also changed. For instance, JGL-22 (type G) is a gypenoside with hydroxyl substitutions at C-23 and C-24, respectively. Thus, the neutral-loss fragment was observed at *m/z* 118 (C_6_H_14_O_2_) in ESI^−^, and the aglycone ions were observed at *m/z* 459, 441 and 423, in ESI^+^.

JGL-18 is a triterpene saponin with a type-H aglycone, whose C-17 side chain is a six-membered heterocyclic oxygen-containing ring. In high-CE ESI^−^, the characteristic neutral-loss fragment was observed at *m/z* 84.0211 (C_4_H_4_O_2_). In high-CE ESI^+^, the aglycone ions were observed at *m/z* 471, 453, and 435 Da, and the fragment ion was identified at *m/z* 341(C_7_H_10_) because of the cleavage of the oxygen-containing heterocycles (Supplementary Figure S4D).

In ESI^−^, gypenosides often produce a [M + HCOO]^−^ adduct ion, and a doubly charged ion can also be obtained at low CE when a gypenoside is connected to two glycosyl chains. In high-CE ESI^−^, the deprotonated molecular ion, neutral-loss fragment ions of the glycosyls, and side-chain cleavage of C-17 were detected. In ESI^+^, aglycone ions were the major fragments, always exhibiting a high response. Therefore, based on the fragment ions obtained in negative-ion mode, the molecular formula, type of sugar moieties, glycosyl connecting position to the aglycone, and structure of C-17 were determined. Further, combined with the fragment ions obtained in both negative- and positive-ion modes, the aglycone could be identified.

### 3.3 Characterization of components of different parts of *G. longipes* with offline 2D-LC/IM-QTOF-MS

#### 3.3.1 Rapid characterization according to aglycone fragments and neutral-loss fragments

To identify the components of *G. longipes* rapidly, a database in UNIFI database format containing the total chemical components of *Gynostemma* was created based on literature published before 2023. This database contains 501 components including triterpenoid saponins, flavonoids, organic acids, and sterols. The major components are triterpenoid saponins, including 434 dammarane-type triterpenoid saponins (Supplementary Table S1). Classify aglycones based on the differences between the substituents and the branched chain structure of C17, as shown in Supplementary Figure S2. In addition, the sugar chains of gypenosides containing rhamnose, xylose, arabinose, and glucose units in different connection orders are classified into 48 types.

**FIGURE 3:**
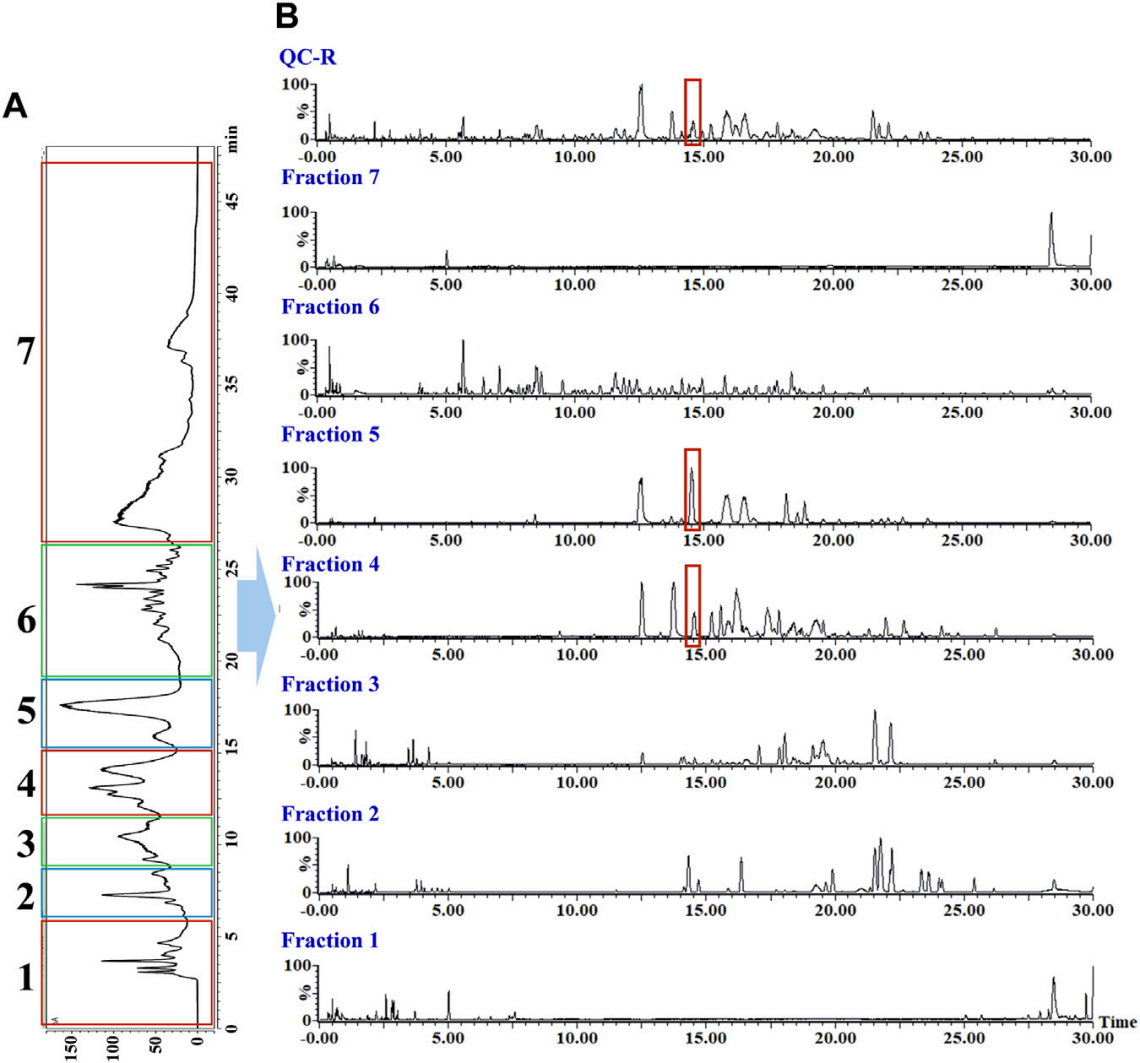
Offline two-dimensional separation chromatogram of rhizomes of *G. longipes*: **(A)** chromatogram from one-dimensional HILIC column and **(B)** chromatogram from two-dimensional RP C18 column.

**FIGURE 4:**
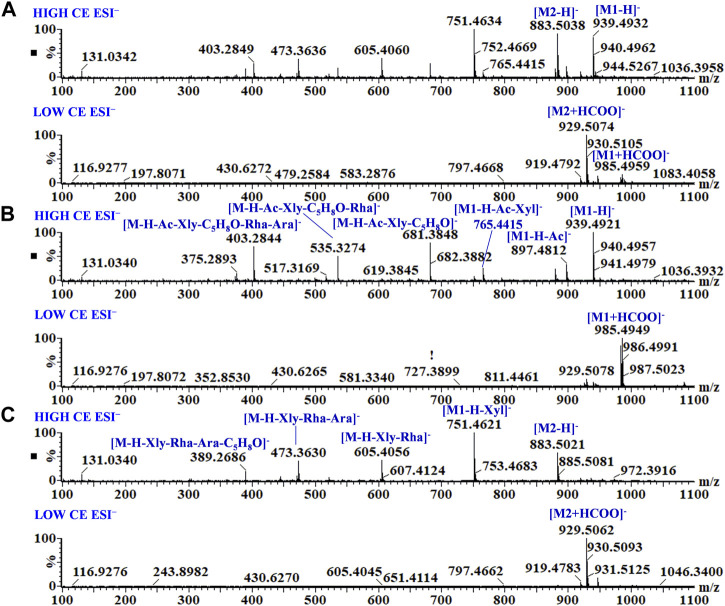
MS and MS^E^ spectra of co-eluted components **(A)**, M1 from fraction 4 **(B)**, and M2 from fraction 5 **(C)**.

As shown in Figure 3, Supplementary Figures S5, S6, the rhizomes, stems, and leaves of *G. longipes* were analyzed integrating with HILIC and RPLC. As a result, the co-eluted components from 1D-RPLC were separated after the analysis of offline 2D-LC/IM-QTOF-MS. A typical example is shown in Figure 3, for which the components co-eluted at 14.55 min were separated into fractions 4 and 5, and their mass fragments were clearly identified (Figure 4). In ESI^−^, M1 produced mass fragments at *m/z* 985, 939, 897, 765, 681, 535, 403, and 375, showing characteristic neutral-loss fragments at *m/z* 84.0573 and 42.0104, indicating that M1 probably contained an acetyl group and a cyclic side chain of C-17. In ESI^+^, M1 produced diagnostic ions at *m/z* 471, 453, and 435. Based on the retention times, fragment ions, and comparison with known compounds in the literature, M1 was tentatively identified as a new compound: 3,20,21-trihydroxy-19-oxo-21,23-epoxydammar-24-ene-3-*O*-[-α-l-rhamnopyranosyl (1→2)] [-β-d-xylopyranosyl (1→3)]-4-*O*-acetyl-α-l-arabinopyranoside. Based on the mass fragments obtained in both negative- and positive-ion modes and the reference standard comparison, M2 was identified as GL-6. Moreover, offline separation significantly enriched the trace components.

Abundant acylated gypenosides, such as acetyl-saponin and malonyl-saponin, have also been found in Herba Gynostemma (Li et al., 2023). For malonyl-gypeniside, the deprotonated molecular ion (rather than the [M + HCOO]^−^ adduct ion) was detected at low CE in ESI^−^. With the improvement in mass CE, the ester bonds were cleaved preferentially and produced characteristic neutral-loss fragments at 42, 44, or 86 Da at high CE in ESI^−^. For example, peak 342 contained fragment ions at *m/z* 1039, 995, 953, 821, 779, 633, 471, and 359 in negative-ion mode. Based on the neutral-loss fragments at 44, 42, 42, and 112 Da, peak 342 contains both malonyl and acetyl substitutions, and the aglycone was type-C. In ESI^+^, diagnostic ions at *m/z* 1058, 455, 437, and 419 were obtained. By comparison with known compounds in the literature, peak 342 was tentatively identified as a novel compound: 3,20,23-trihydroxydammar-24-en-21-oic acid-21,23-lactone-3-*O*-[4-*O*-acetyl-α-l-rhamnopyranosyl (1→2)]-[β-d-xylopyranosyl (1→3)]-6-*O*-malonyl-β-d-glucopyranoside.

In ESI^−^, peaks 86 and 175 yielded the same adduct ion and doubly charged ion at low CE, indicating that these peaks represent a pair of isomers. At a high CE in ESI^−^, peak 86 produced fragment ions at *m/z* 1061, 929, 767, 667, 521, and 389. The sugar moieties were determined from the neutral-loss fragments at 132, 146, and 162 Da, and the neutral-loss fragment (100 Da) indicates that the C17 side chain was connected to an oxygen-containing substituent. In the ESI^+^, peak 86 produced aglycone ions at *m/z* 455, 437, and 423. By comparison with known compounds in the literature, peak 86 was tentatively identified as 3β,20S,21-trihydroxy-25-methoxydammar-23-ene 3-*O*-α-l-rhamnopyranosyl (1→2)-[β-d-glucopyranosyl (1→3)]-β-d-arabinopyranosyl-21-*O*-β-d-xylopyranoside. Based on a comparison with the reference standard, retention time, and fragment ions, peak 175 was identified as GL-7. However, some isomers with the same mass fragments still could not be accurately identified.

#### 3.3.2 Isomer discrimination by CCS values

**FIGURE 5:**
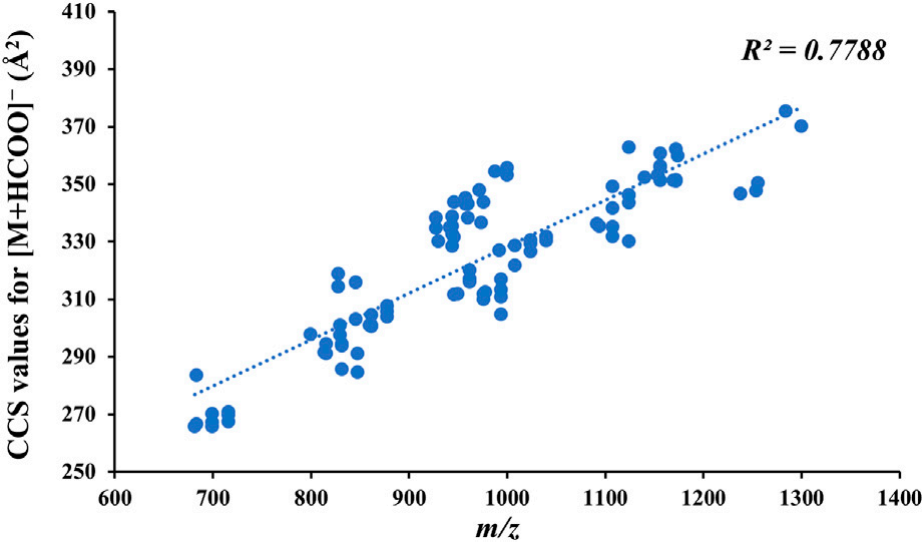
Relationship between the mass-to-charge ratio of compounds and their CCS values for [M + HCOO]^−^.

Owing to the good response of [M + HCOO]^−^ in negative-ion mode at a low CE, 100 CCS values for the reference standards for [M + HCOO]^−^ were obtained, and there was a positive correlation between the mass-to-charge ratio of compounds and their CCS values (Figure 5). The CCS value is an additional parameter related to the size, shape, and charge of gas-phase ions and can be used to identify isomers (Tu et al., 2019). Therefore, the CCS of the isomers in the reference standards were compared. As shown in Table 1, the CCS values of the *S*-configuration were lower than those of the *R*-configuration when the structural difference was only *R*/*S* isomerism, such as in GL-12 (20*S*, 23*S*) and GL-13 (20*R*, 23*R*). Comparing the CCS values of the substituted isomers and those having different positions of -OH or -OOH, it was found that the CCS value of the C-24*S* configuration was smaller than those of the C-24*R* configuration, C-26 substituted isomer, and C-25 substituted isomer, such as FJ-69 (C24-OH(*S*)), FJ-72 (C24-OH(*R*)), FJ-70 (C26-OH), and FJ-67 (C25-OH).

**TABLE 1:** Comparison of the measured and predicted CCS values of reference standards for [M + HCOO]^−^.

No.	Formula	Calc. Mass	configuration	Measured (Å^2^)	Predicted (Å^2^)	Deviations (%)	No.	Formula	Calc. Mass	configuration	Measured (Å^2^)	Predicted (Å^2^)	Deviations (%)
GL-1	C_47_H_78_O_17_	914.5239	20*R*	343.22	333.2	3.01	FJ-12	C_41_H_70_O_14_	786.4766	20*S*, 24*S*	285.71	287.2	0.52
GL-2	C_46_H_76_O_17_	900.5083	20*S*	311.71	324.5	3.94	FJ-13	C_48_H_82_O_19_	962.5450	20*S*	328.76	334.7	1.77
GL-3	C_47_H_76_O_17_	912.5083	20*S*, 23*S*	345.22	331.2	4.23	FJ-14	C_41_H_70_O_14_	786.4766	20*S*	293.87	285.6	2.89
GL-4	C_47_H_76_O_17_	912.5083	20*R*, 23*R*	343.26	331.2	3.64	FJ-17	C_36_H_62_O_11_	670.4292	20*S*, 24*S*	267.48	264.2	1.24
GL-5	C_47_H_76_O_17_	912.5083	20*S*, 23*S*	344.54	332.8	3.53	FJ-18	C_36_H_62_O_9_	638.4394	20*S*	283.73	265.1	7.03
GL-6	C_46_H_76_O_16_	884.5133	20*S*	330.30	328.1	0.67	FJ-19	C_36_H_62_O_9_	638.4394	20*S*	266.77	264.8	0.74
GL-7	C_53_H_90_O_21_	1062.5975	20*S*	349.22	344.2	1.46	FJ-20	C_42_H_72_O_13_	784.4973	20*S*	300.99	295.3	1.93
GL-8	C_46_H_72_O_17_	896.4770	20*S*, 23*S*	335.05	328.6	1.96	FJ-22	C_42_H_70_O_13_	782.4816	n	318.87	295.1	8.05
GL-10	C_52_H_88_O_21_	1048.5818	20*S*	335.45	342.7	2.11	FJ-23	C_41_H_70_O_12_	754.4867	20*S*	298.02	286.4	4.06
GL-11	C_53_H_90_O_22_	1078.5924	20*S*	346.04	343.9	0.62	FJ-24	C_42_H_72_O_13_	784.4973	20*S*	297.67	296.0	0.56
GL-12	C_46_H_74_O_16_	882.4977	20*S*, 23*S*	334.74	329.6	1.56	FJ-25	C_36_H_60_O_9_	636.4237	20*S*	265.77	264.9	0.33
GL-13	C_46_H_74_O_16_	882.4977	20*R*, 23*R*	338.45	329.6	2.68	FJ-26	C_42_H_70_O_13_	782.4816	n	314.51	294.8	6.68
GL-14	C_49_H_78_O_18_	954.5188	20*S*, 23*S*	353.31	331.3	6.64	FJ-27	C_47_H_80_O_17_	916.5396	20*S*	317.15	331.2	4.24
GL-15	C_49_H_78_O_18_	954.5188	20*R*, 23*R*	355.77	331.3	7.39	FJ-28	C_42_H_72_O_14_	800.4922	20*S*	316.03	299.7	5.45
GL-17	C_48_H_78_O_18_	942.5188	20*S*, 23*S*	354.53	333.0	6.46	FJ-30	C_36_H_62_O_11_	670.4292	20*S*, 24*R*	269.74	264.2	2.10
GL-18	C_47_H_80_O_16_	900.5446	20*S*	343.92	331.5	3.75	FJ-31	C_36_H_62_O_10_	654.4343	20*S*	270.2	265.2	1.88
GL-19	C_48_H_78_O_17_	926.5239	23*S*	347.97	333.1	4.46	FJ-32	C_36_H_62_O_11_	670.4292	20*S*	270.97	265.5	2.06
GL-20	C_52_H_86_O_21_	1046.5662	20*S*	336.28	342.7	1.87	FJ-33	C_41_H_70_O_14_	786.4766	20*S*	294.67	291.1	1.23
GL-21	C_46_H_74_O_17_	898.4926	21*S*	338.87	331.2	2.32	FJ-34	C_41_H_68_O_13_	768.4660	20*S*	291.65	285.0	2.33
JGL-1	C_46_H_74_O_17_	898.4926	20*R*, 21*R*, 23*R*, 24*R*	328.49	324.3	1.29	FJ-35	C_36_H_62_O_10_	654.4343	20*S*	267.26	265.7	0.59
JGL-2	C_53_H_90_O_22_	1078.5924	20*S*, 24*R*	362.86	352.5	2.94	FJ-36	C_36_H_62_O_10_	654.4343	20*S*, 24*S*	265.78	264.5	0.48
JGL-3	C_52_H_86_O_22_	1062.5611	20*S*, 24*R*	335.29	342.3	2.05	FJ-37	C_41_H_70_O_13_	770.4816	20*S*, 24*S*	291.14	286.4	1.65
JGL-4	C_46_H_74_O_18_	914.4875	21*S*, 23*S*	338.40	331.2	2.17	FJ-41	C_47_H_80_O_18_	932.5345	20*S*	312.52	332.1	5.89
JGL-5	C_47_H_76_O_18_	928.5032	20*R*, 21*R*, 23*R*, 24*R*	336.71	325.4	3.48	FJ-42	C_48_H_82_O_19_	962.5450	20*S*	321.86	333.4	3.46
JGL-6	C_47_H_78_O_18_	930.5188	20*R*, 21*R*, 23*R*, 24*R*	343.75	326.1	5.41	FJ-43	C_53_H_90_O_21_	1062.5975	20*S*	341.81	343.2	0.41
JGL-7	C_46_H_76_O_17_	900.5083	20*R*, 21*R*, 23*R*, 24*R*	331.71	324.2	2.32	FJ-48	C_48_H_82_O_18_	946.5501	20*S*	326.96	332.9	1.78
JGL-8	C_58_H_96_O_26_	1208.6190	20*S*	347.77	341.8	1.75	FJ-49	C_47_H_78_O_18_	930.5188	20*S*	311.92	323.9	3.70
JGL-9	C_58_H_96_O_25_	1192.6241	20*R*	346.79	338.6	2.42	FJ-50	C_47_H_80_O_19_	948.5294	20*S*, 24*S*	304.94	329.0	7.31
JGL-10	C_59_H_98_O_27_	1238.6295	20*S*, 21*R*, 23*R*	375.56	349.1	7.58	FJ-52	C_47_H_78_O_18_	930.5188	20*S*	310.16	326.2	4.92
JGL-11	C_58_H_98_O_26_	1210.6346	20*S*	350.61	341.6	2.64	FJ-54	C_47_H_80_O_19_	948.5294	20*S*	313.34	332.2	5.68
JGL-13	C_60_H_102_O_27_	1254.6608	20*S*	370.24	342.8	8.00	FJ-56	C_53_H_90_O_22_	1078.5924	20*S*	343.55	351.5	2.26
JGL-14	C_53_H_90_O_22_	1078.5924	20*S*, 24*S*	346.45	352.5	1.72	FJ-57	C_47_H_80_O_19_	948.5294	20*S*, 24*R*	310.96	329.0	5.48
JGL-15	C_53_H_90_O_22_	1078.5924	20*S*, 21*S*, 24*S*	330.17	351.6	6.09	FJ-58	C_48_H_82_O_20_	978.5399	20*S*	330.63	334.1	1.04
JGL-16	C_52_H_86_O_22_	1062.5611	20*S*, 24*S*	331.90	342.3	3.04	FJ-59	C_48_H_82_O_20_	978.5399	20*S*, 24*R*	329.33	333.0	1.10
JGL-17	C_46_H_74_O_17_	898.4926	20*S*, 21*S*, 23*S*, 24*S*	332.73	324.3	2.60	FJ-61	C_48_H_82_O_21_	994.5349	20*S*, 24*R*	331.74	332.5	0.23
JGL-18	C_46_H_74_O_17_	898.4926	20*ξ*, 21*ξ*, 23*ξ*	335.35	328.6	2.05	FJ-62	C_48_H_82_O_21_	994.5349	20*S*	331.91	333.5	0.48
JGL-19	C_46_H_76_O_18_	916.5032	20*S*, 21*S*, 23*S*, 24*S*	316.17	330.9	4.45	FJ-64	C_48_H_82_O_20_	978.5399	20*S*, 24*S*	326.59	333.0	1.92
JGL-20	C_46_H_76_O_18_	916.5032	20*S*, 21*R*, 23*R*, 24*R*	320.08	330.9	3.27	FJ-65	C_48_H_82_O_21_	994.5349	20*S*, 24*S*	330.35	332.5	0.65
JGL-22	C_46_H_80_O_17_	904.5396	20S	311.98	325.5	4.15	FJ-66	C_53_H_90_O_23_	1094.5873	20*S*	352.47	347.1	1.55
FJ-1	C_42_H_72_O_14_	800.4922	20*S*	303.12	296.8	2.13	FJ-67	C_53_H_90_O_24_	1110.5822	20*S*	360.81	347.2	3.92
FJ-2	C_41_H_70_O_13_	770.4816	20*S*	294.64	285.9	3.06	FJ-68	C_53_H_90_O_25_	1126.5771	20*S*	362.37	347.3	4.34
FJ-3	C_42_H_72_O_16_	832.4820	20*S*	307.71	300.6	2.36	FJ-69	C_53_H_90_O_24_	1110.5822	20*S*, 24*S*	351.29	347.7	1.03
FJ-4	C_42_H_72_O_15_	816.4871	20*S*, 24*R*	301.17	298.4	0.93	FJ-70	C_53_H_90_O_24_	1110.5822	20*S*	356.35	340.5	4.65
FJ-5	C_42_H_72_O_16_	832.4820	20*S*, 24*R*	305.80	297.5	2.79	FJ-71	C_53_H_90_O_25_	1126.5771	20*S*, 24*S*	351.25	348.2	0.87
FJ-6	C_42_H_72_O_16_	832.4820	20*S*, 24*S*	303.90	297.5	2.15	FJ-72	C_53_H_90_O_24_	1110.5822	20*S*, 24*R*	356.00	347.7	2.39
FJ-7	C_42_H_72_O_15_	816.4871	20*S*	304.53	299.4	1.71	FJ-73	C_47_H_80_O_19_	948.5294	20*S*	316.91	331.6	4.43
FJ-8	C_42_H_72_O_15_	816.4871	20*S*, 24*S*	300.56	298.4	0.72	FJ-74	C_53_H_88_O_24_	1108.5666	20*S*	353.27	342.1	3.26
FJ-9	C_42_H_70_O_15_	814.4715	20*S*	301.01	294.7	2.14	FJ-75	C_53_H_92_O_25_	1128.5928	20*S*	359.90	347.8	3.48
FJ-10	C_41_H_70_O_15_	802.4715	20*S*, 24*S*	284.65	292.9	2.82	FJ-76	C_54_H_92_O_24_	1124.5979	20*S*	351.35	346.9	1.28
FJ-11	C_41_H_70_O_15_	802.4715	20*S*	291.13	293.7	0.87	FJ-77	C_53_H_90_O_25_	1126.5771	20*S*, 24*R*	351.53	348.2	0.96

n: no *R*/*S* configuration.

The predicted CCS values also showed great potential for isomer discrimination, and 434 gypenosides in the database were predicted using CCSondemand. As shown in Supplementary Table S3, 2,170 CCS values for [M + H]^+^, [M + Na]^+^, [M + K]^+^, [M−H]^−^, and [M + HCOO]^−^ were predicted. A comparison of the differences between the measured and predicted CCS values for each [M + HCOO]^−^ reference standard is shown in Table 1, and the deviations were calculated using Eq. (1). Compared to the measured values, the deviations of predicted CCS values obtained using the CCSondemand prediction platform ranged from 0.23% to 8.05%. A deviation of less than 2% accounted for 38% of the total, and a deviation value between 2% and 4% accounted for 37% of the total amount.

$$Deviation\;\left(\%\right)=\left|\left(measured\;value-predicted\;value\right)\right|÷predicted\;value\times 100\%$$ (1)

The measured and predicted CCS values were combined to discriminate and identify isomers. For example, peaks 189 and 197 had the same mass fragments in both negative- and positive-ion modes. Compared with the measured CCS values for [M + HCOO]^−^, the CCS values of peak 189 was 335.05 Å^2^, and the CCS value of peak 197 was 339.48 Å^2^. Based on the reference standards, peak 189 was identified as GL-8, and peak 197 was tentatively identified as 23*R*-GL-8. Combined CCS values with retention times and fragment ions together to analyze could increase the accuracy of identification. However, for the acylated gypenoside, the positions of the acetyl and malonyl groups could not be accurately discriminated, even using the predicted CCS values.

**FIGURE 6:**
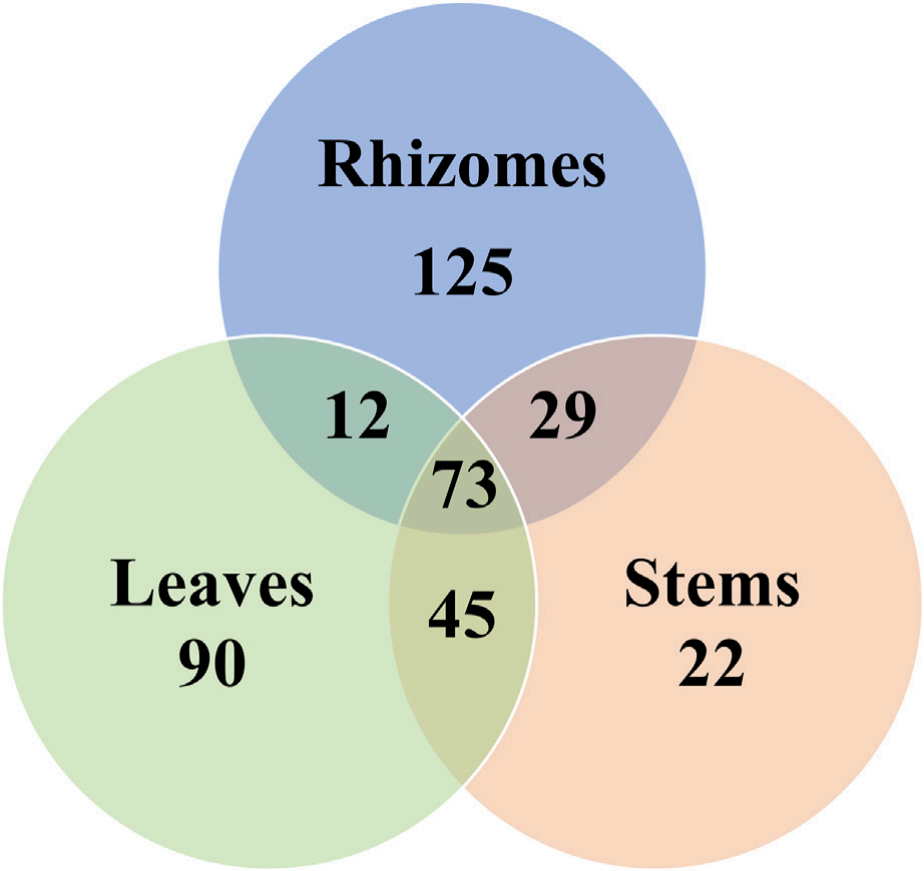
Venn diagram of the composition in different parts of *G. longipes*.

Based on their retention times, fragment ions, and measured and predicted CCS values, 396 components were identified from the different parts of *G. longipes*, of which 217 components were identified or tentatively identified as new compounds and 94 groups of isomers. Most of the new compounds were gypenosides having substituted malonyl and acetyl groups, and detailed information for identification is listed in Supplementary Table S4. The components identified in each plant part are shown in Figure 6: the rhizomes contained the largest number of components, and the stems contained the least. The leaves and stems contained the most components in common, whereas the difference in composition between rhizomes and leaves was the greatest.

### 3.4 Differential component analysis in different parts of *G. longipes*

**FIGURE 7:**
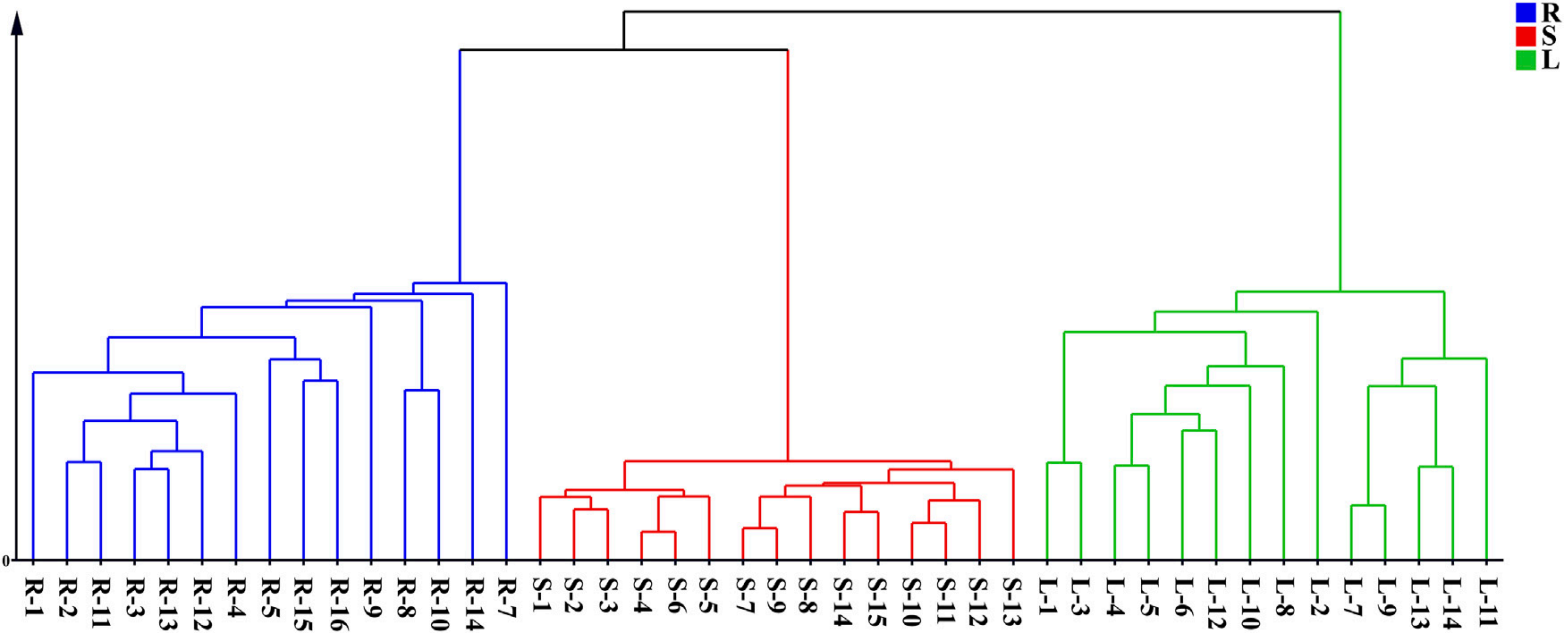
HCA diagram of different parts of *G. longipes*.

**FIGURE 8:**
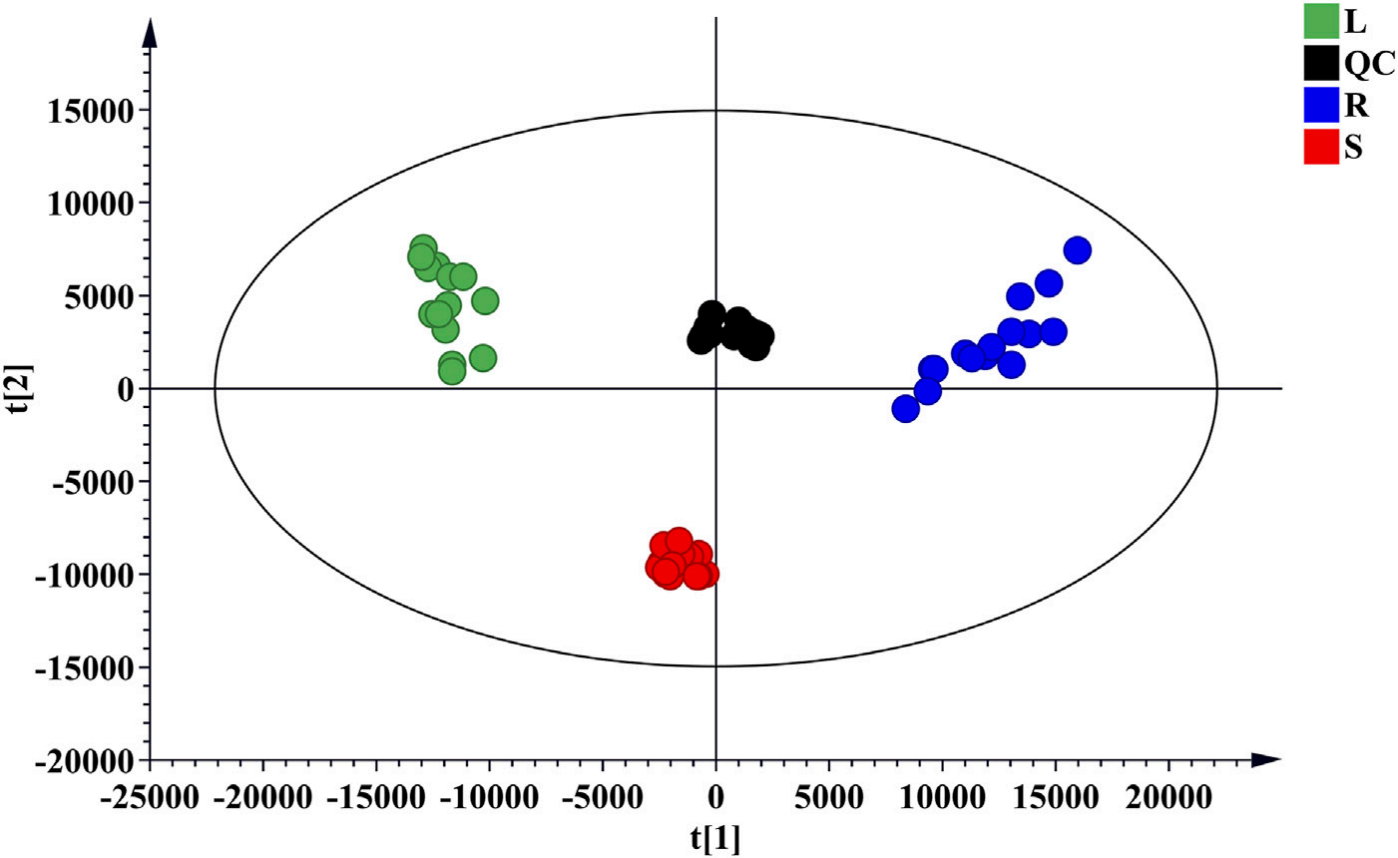
PCA score plot of different parts of *G. longipes*.

Forty-four batches of *G. longipes* comprising different parts of the plant were analyzed using 1D-RPLC/IM-QTOF-MS. Through peak extraction, alignment, and normalization, the MS data were preprocessed and imported into SIMCA for HCA and PCA. HCA was used to divide the samples into specific groups according to similarity criteria, as shown in Figure 7, where the samples are clearly divided into three categories: the rhizomes, stems, and leaves were classified as different categories, indicating that the samples from different plant parts showed significant variation. The PCA analysis is shown in Figure 8, where each point represents an individual sample. Samples were clustered into three groups representing the different parts, and the results are similar to those of HCA.

**FIGURE 9:**
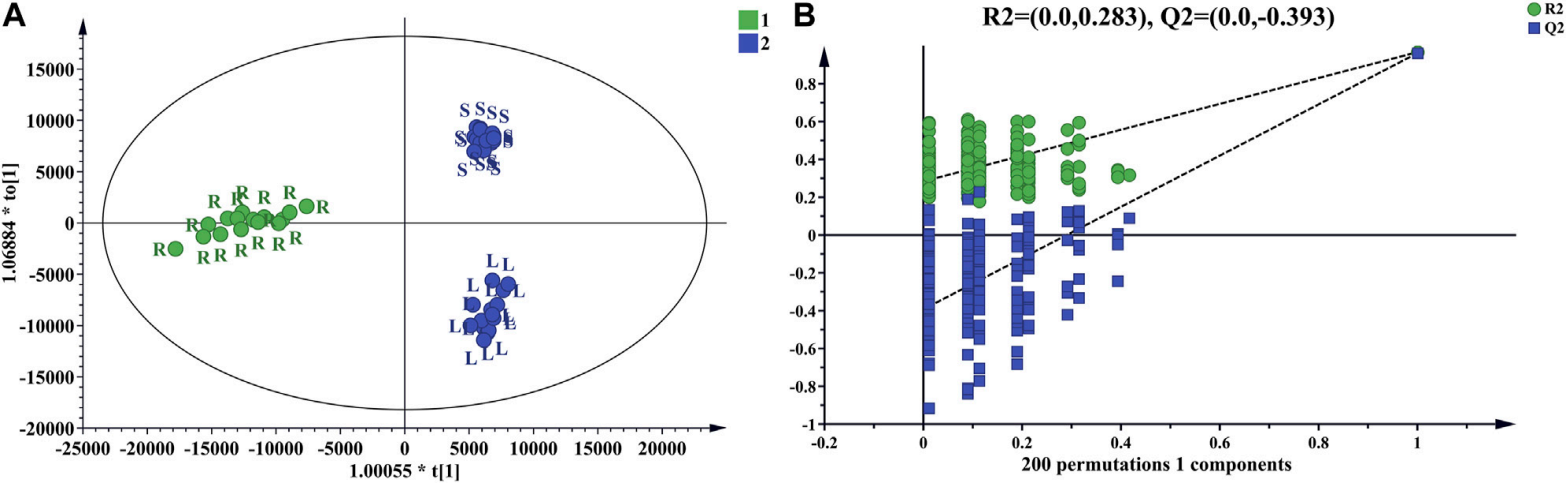
OPLS-DA score plot of rhizomes and aerial parts of *G. longipes* **(A)**, and cross-validation of the OPLS-DA model **(B)**.

**FIGURE 10:**
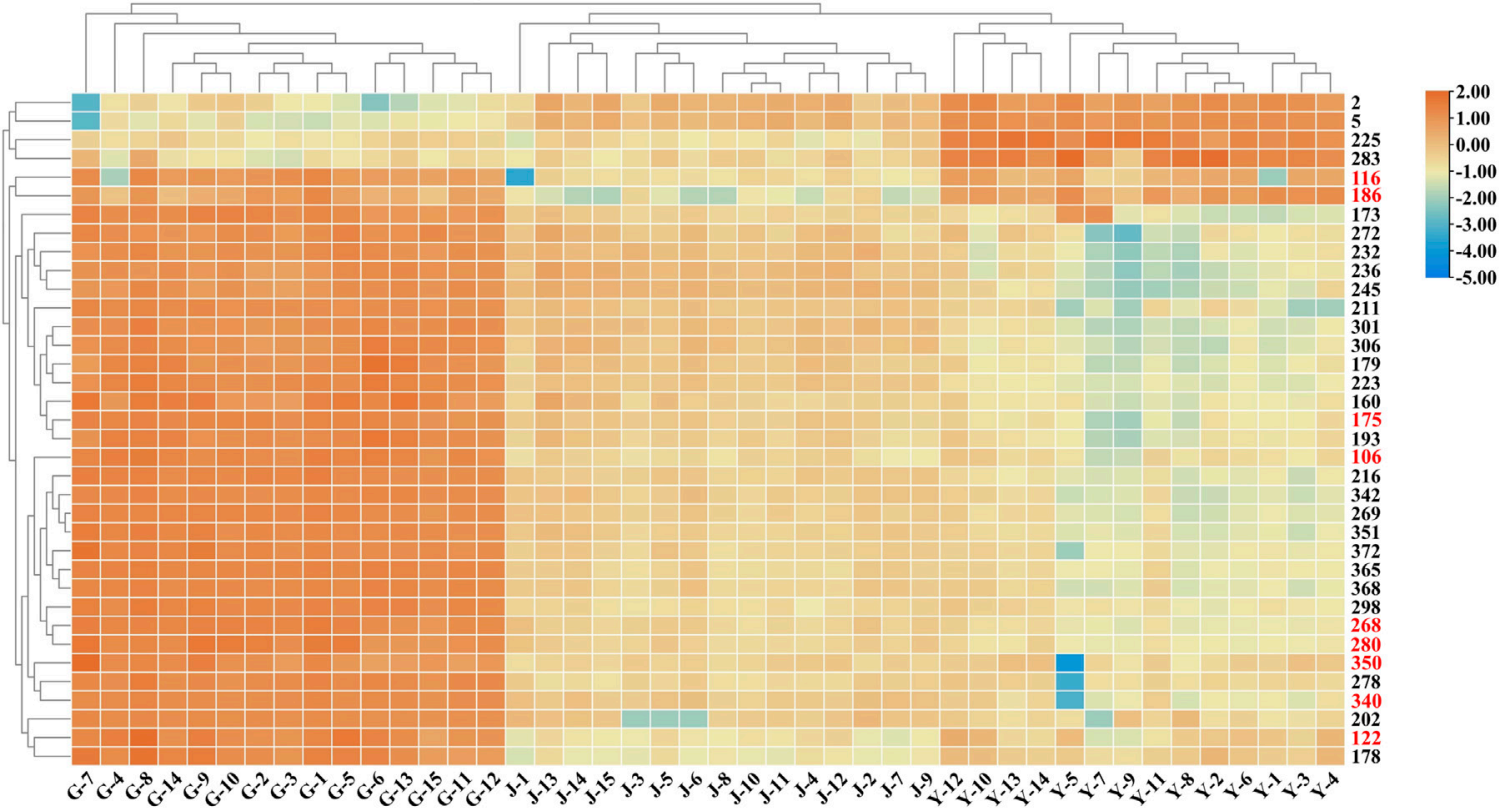
Heatmap based on relative abundance of 36 differential compounds in different parts of *G. longipes*. The rows represent the compounds (peak numbers are consistent with those in Supplementary Table S4, the compounds with red numbers are compared with reference standards).

**FIGURE 11:**
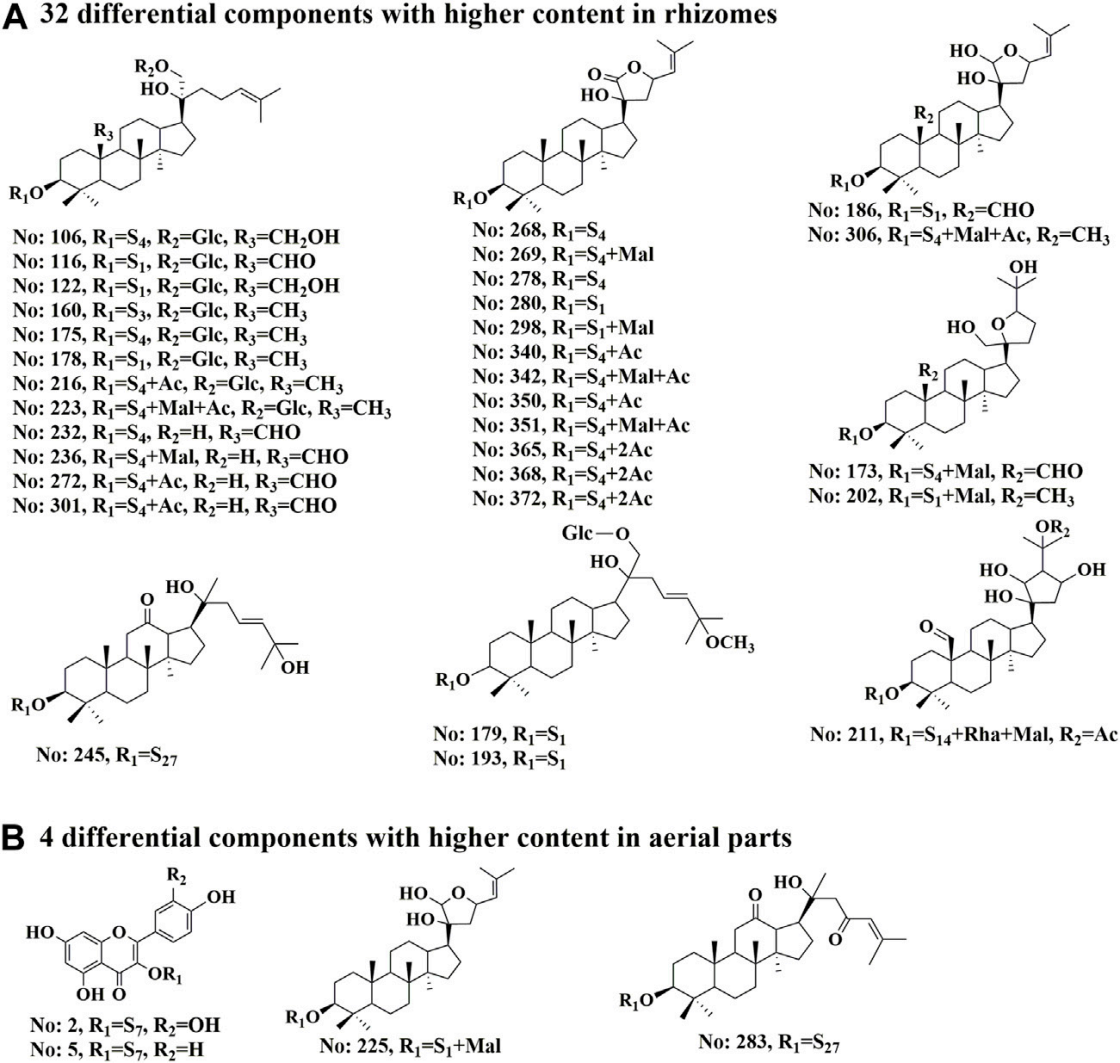
Structures of 36 differential compounds in different parts of *G. longipes*. **(A)** 32 differential components with higher content in rhizomes. **(B)** 4 differential components with higher content in aerial parts. The peak numbers are the same as those in Supplementary Table S4. S presents the sugar chain structures, which are the same as those in Supplementary Figure S2.

To clarify the differences between the underground and aerial parts, rhizomes (Group 1) and stems and leaves (Group 2) were selected for OPLS-DA. Groups 1 and 2 were distributed on the negative and positive sides of the *t1* axis, respectively, indicating significant differences between the two groups (Figure 9). The parameters were *R2X* = 0.642, *R2Y* = 0.966, and *Q2* = 0.961, indicating that 64.2% of the variables (*R2X*) in the model explained 96.6% of the difference (*R2Y*) between the two groups, and the predictive ability of the model was 96.1%. After 200 permutations tests, the intercept of *R2* was 0.283 and the intercept of *Q2* was −0.393, both lower than the theoretical values (*R2* ≤ 0.3, *Q2* ≤ 0.05), indicating that the model was not over-fitted. Based on the OPLS-DA and VIP plots, the variables with VIP values greater than 6.5 were considered as potential chemical markers. Thirty-six differential components were identified, including two flavonoids and 34 triterpenoid saponins, 20 of which were substituted with a malonyl group and (or) acetyl group. A heatmap (Figure 10) was used to visualize the trends in these 36 differential components, and 32 gypenosides were found to be higher in content in the rhizomes. These results combined with the results of total component identification indicate that the composition and content of chemical components in the rhizomes are both higher than in the other parts of the plant, and the structures of these differential compounds are shown in Figure 11. Overall, these findings indicate that the rhizomes are a medicinally important part of *G. longipes*. As reported in the literature (Teng et al., 2022), the total saponins in the underground part of *G. pentaphyllum* from Shaanxi province also have good ability to reduce blood lipids; therefore, both the aerial and underground parts of *G. longipes* are recommended for medicinal use.

## 4 Conclusion

In this study, an offline 2D-LC/IM-QTOF-MS system was established to analyze the chemical components of the different parts of *G. longipes*. Offline 2D chromatographic separation based on HILIC and RPLC not only separated the components co-eluted in 1D-RPLC but also significantly enriched the trace components. Using reference standards analysis, the characteristic aglycone fragments and neutral-loss fragments for both positive and negative ions were summarized. And, a total component mass spectrometry database for Herba Gynostemma was established, and the aglycons of triterpenoid saponins have been summarized for rapid identification. A total of 396 components were identified in the three plant parts, and 217 components were identified or tentatively identified as new compounds, most of which were gypenosides substituted with malonyl and acetyl groups. Based on the measured and predicted CCS values, 94 groups of isomers were identified. As shown in the results, multiple parameters combination analysis could increase the accuracy of identification. Through the systematic identification of the full components in different parts of *G. longipes* and the analysis of the differential components between rhizomes and aerial parts (stems and leaves), it was found that the rhizomes contained the largest variety of components, and the content of 32 differential components was also higher. Gypenosides has numbers of pharmacological effects, such as anticancer, cardioprotective, hepatoprotective, neuroprotective, and anti-inflammatory activities (Nguyen et al., 2021). In addition, Teng et al. (2022) using the rat model of hyperlipidemia to study the lipid-lowering activity of total saponins in the aerial part and underground part of *G*. *pentaphyllum*. The results showed that the gypenosides from the underground parts also have good lipid-lowering activity. In summary, the rhizome of *G. longipes* is also an important plant part that should be considered for medicinal use.

## Data Availability

The original contributions presented in the study are included in the article/Supplementary Material, further inquiries can be directed to the corresponding author.
