# Supplementary material for: Two-dimensional liquid chromatography and ion mobility-mass spectrometry for the multicomponent characterization of different parts of the medicinal plant Gynostemma longipes
Source: Front Chem. 2023 Sep 1;11:1203418. doi: 10.3389/fchem.2023.1203418 (PMC10502315; doi:10.3389/fchem.2023.1203418)
Supplement: Supplementary file 1 [file DataSheet1.docx]

Two-dimensional liquid chromatography and ion mobility-mass spectrometry for the multicomponent characterization of different parts of the medicinal plant *Gynostemma longipes*

Wei Zheng^1^, Gang Li^1^, Guang Yang^1^, Pengxin Lu^1^, Qi Li^1^, Mengmeng Zhang^1^, Ming Yuan^2^, Xiaojuan Chen^1^, Chenchen Wang^3^, Baolin Guo^4^, Baiping Ma^1*^

^1^Beijing Institute of Radiation Medicine, Beijing, China

^2^Waters Technologies Limited, Shanghai, China

^3^Shaanxi Cuiyuankang Health Industry Group Co. LTD., Shaanxi, China

^4^Institute of Medicinal Plant Development, Chinese Academy of Medical Sciences, Peking Union Medical College, Beijing, China

*** Correspondence:**Baiping Ma
mabaiping@sina.com

**Supplementary Material (Figure S1-S6, Tables S1-S4).**

**Figure S1** Aglycone structures of gypenosides in the Herba Gynostemma mass spectrometry database.

**Figure S2** Sugar chain structures of gypenosides in the Herba Gynostemma mass spectrometry database.

**Figure S3** Scatter plots of separation differences between one-dimensional candidate columns (horizontal axis) and two-dimensional C18 columns (vertical axis) for 39 reference standards. The mobile phase in (A) – (D) were 0.1% formic acid in water–0.1% formic acid in acetonitrile, and the mobile phase of (E) – (H) was 0.2% formic acid in water–acetonitrile.

**Figure S4** MS and MS^E^ spectra of JGL-4 (A), GL-14 (B), GL-7 (C), and JGL-18 (D).

**Figure S5** Offline two-dimensional separation chromatogram of stems of *G. longipes*: (A) chromatogram from one-dimensional HILIC column and (B) chromatogram from two-dimensional RP C18 column.

**Figure S6** Offline two-dimensional separation chromatogram of leaves of *G. longipes*: (A) chromatogram from one-dimensional HILIC column and (B) chromatogram from two-dimensional RP C18 column.

**Table S1** Structure list of gypenosides in the Herba Gynostemma mass spectrometry database.

**Table S2** Grouping list of mixed reference standards.

**Table S3** CCS prediction values of gypenosides in the Herba Gynostemma mass spectrometry database based on CCSondemand.

**Table S4** Identification of chemical components from the three parts of *G. longipes*.








**Figure S1** Aglycone structures of gypenosides in the Herba Gynostemma mass spectrometry database.


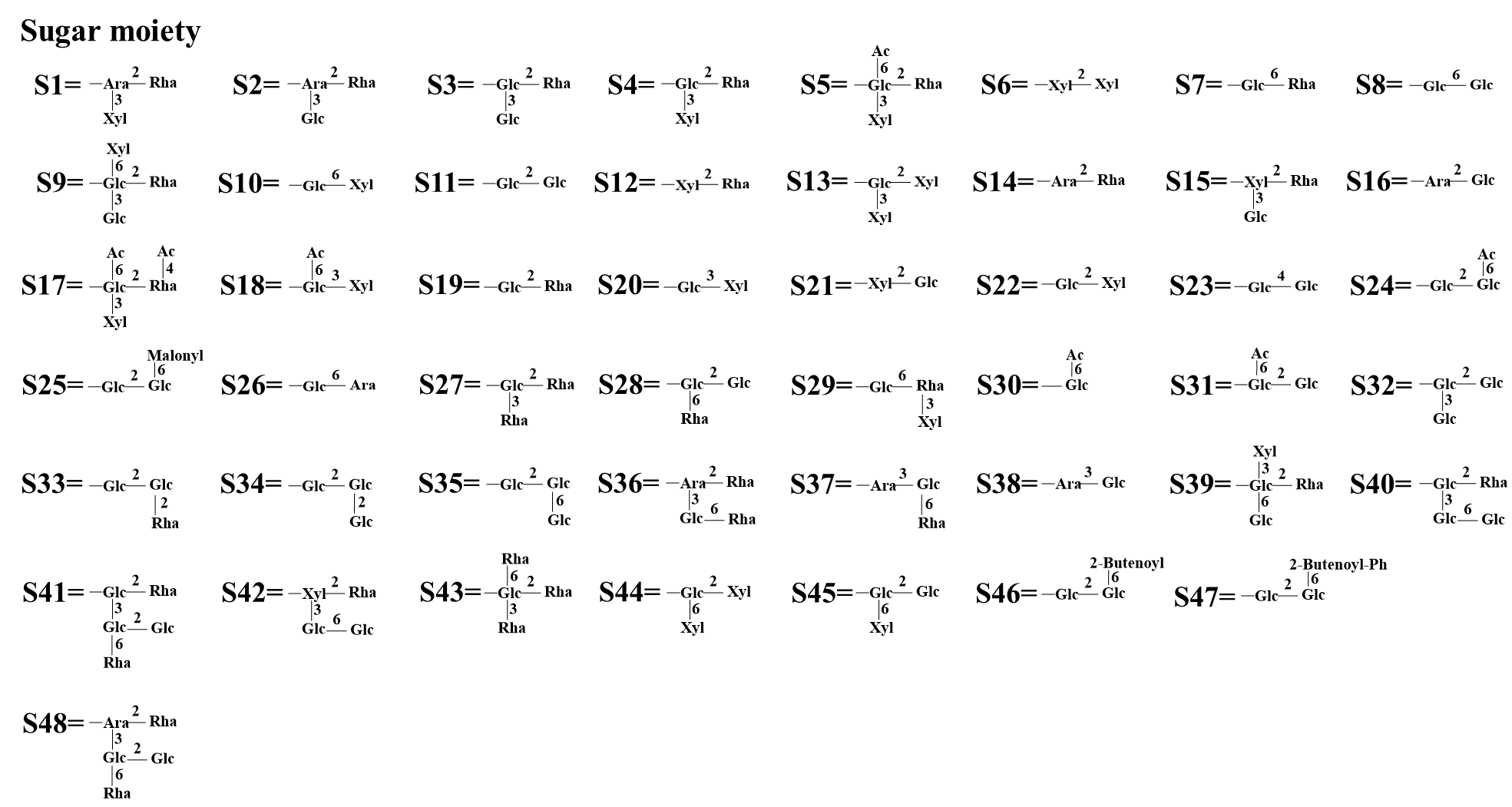


**Figure S2** Sugar chain structures of gypenosides in the Herba Gynostemma mass spectrometry database.


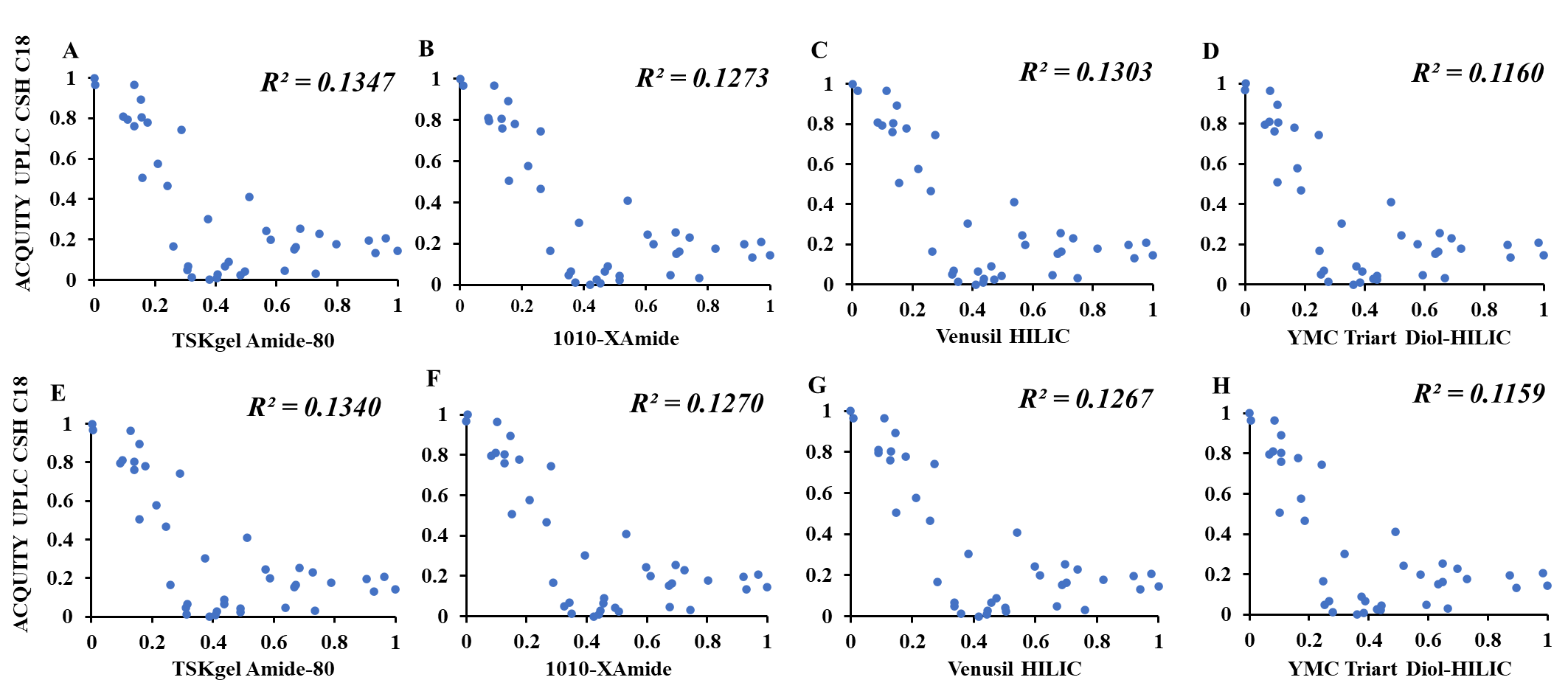


**Figure S3** Scatter plots of separation differences between one-dimensional candidate columns (horizontal axis) and two-dimensional C18 columns (vertical axis) for 39 reference standards. The mobile phase in (A) – (D) were 0.1% formic acid in water–0.1% formic acid in acetonitrile, and the mobile phase of (E) – (H) was 0.2% formic acid in water–acetonitrile.


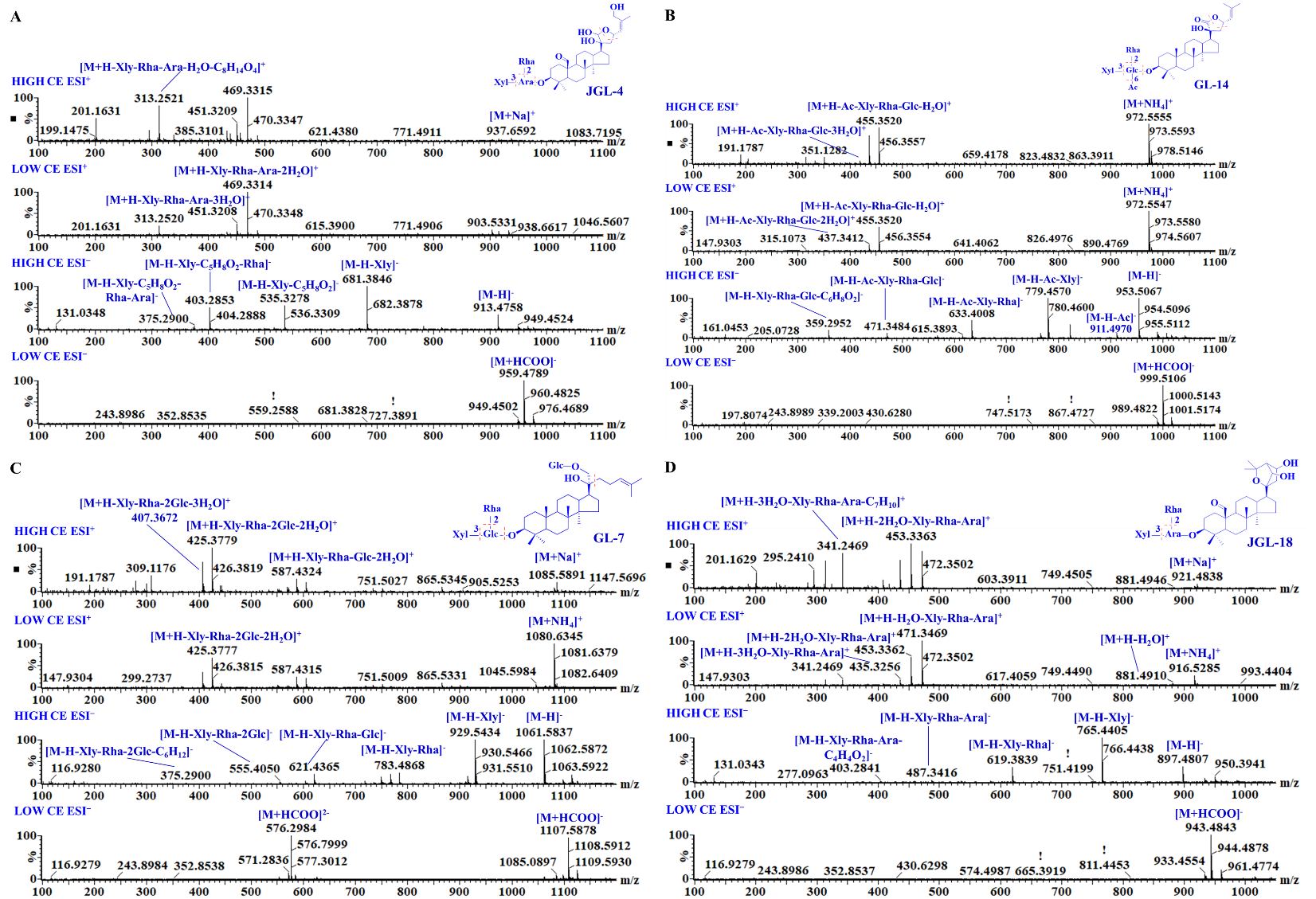


**Figure S4** MS and MS^E^ spectra of JGL-4 (A), GL-14 (B), GL-7 (C), and JGL-18 (D)

**
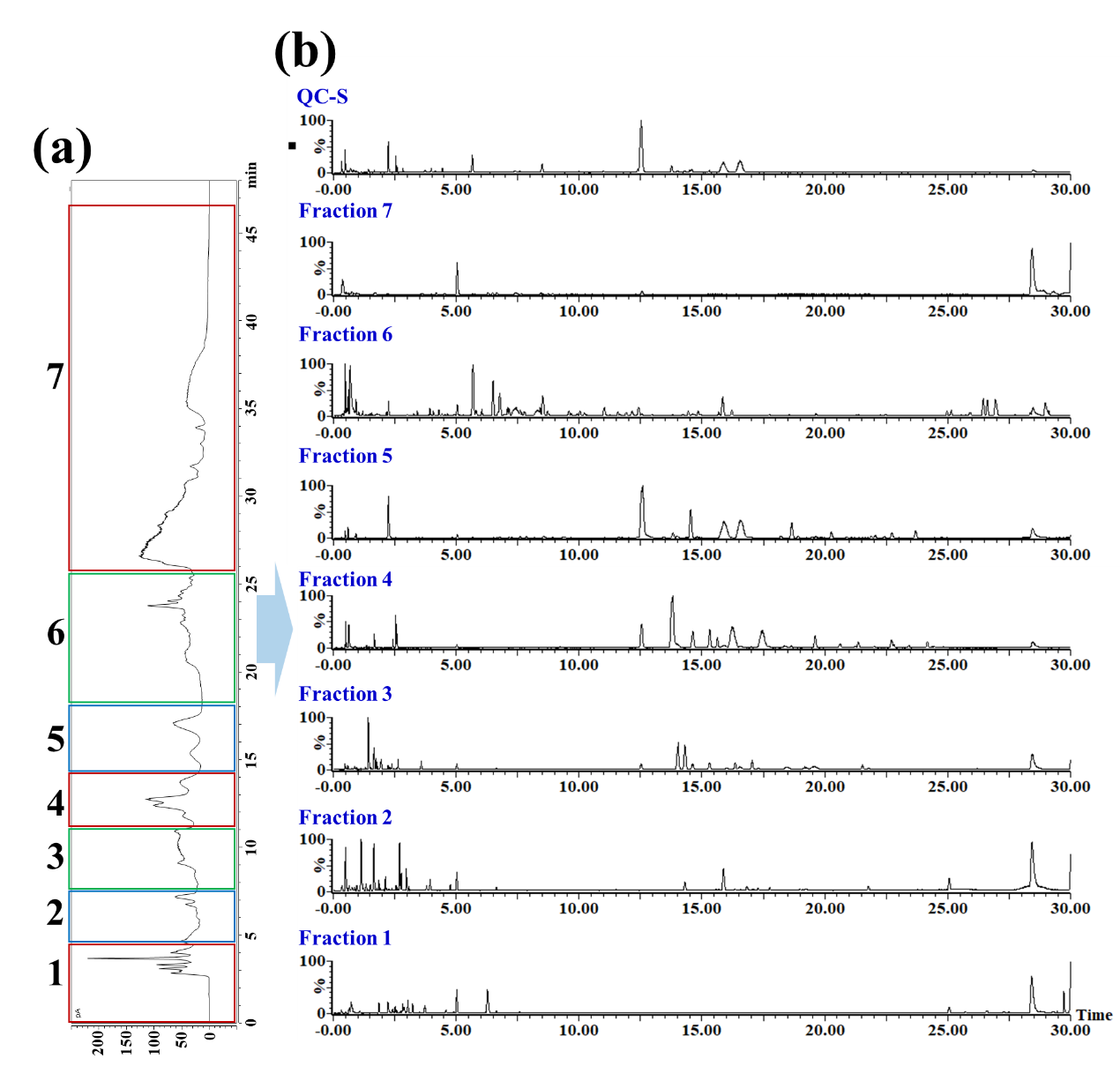
**

**FIGURE S5** Offline two-dimensional separation chromatogram of stems of *G. longipes*: (A) chromatogram from one-dimensional HILIC column and (B) chromatogram from two-dimensional RP C18 column.

**
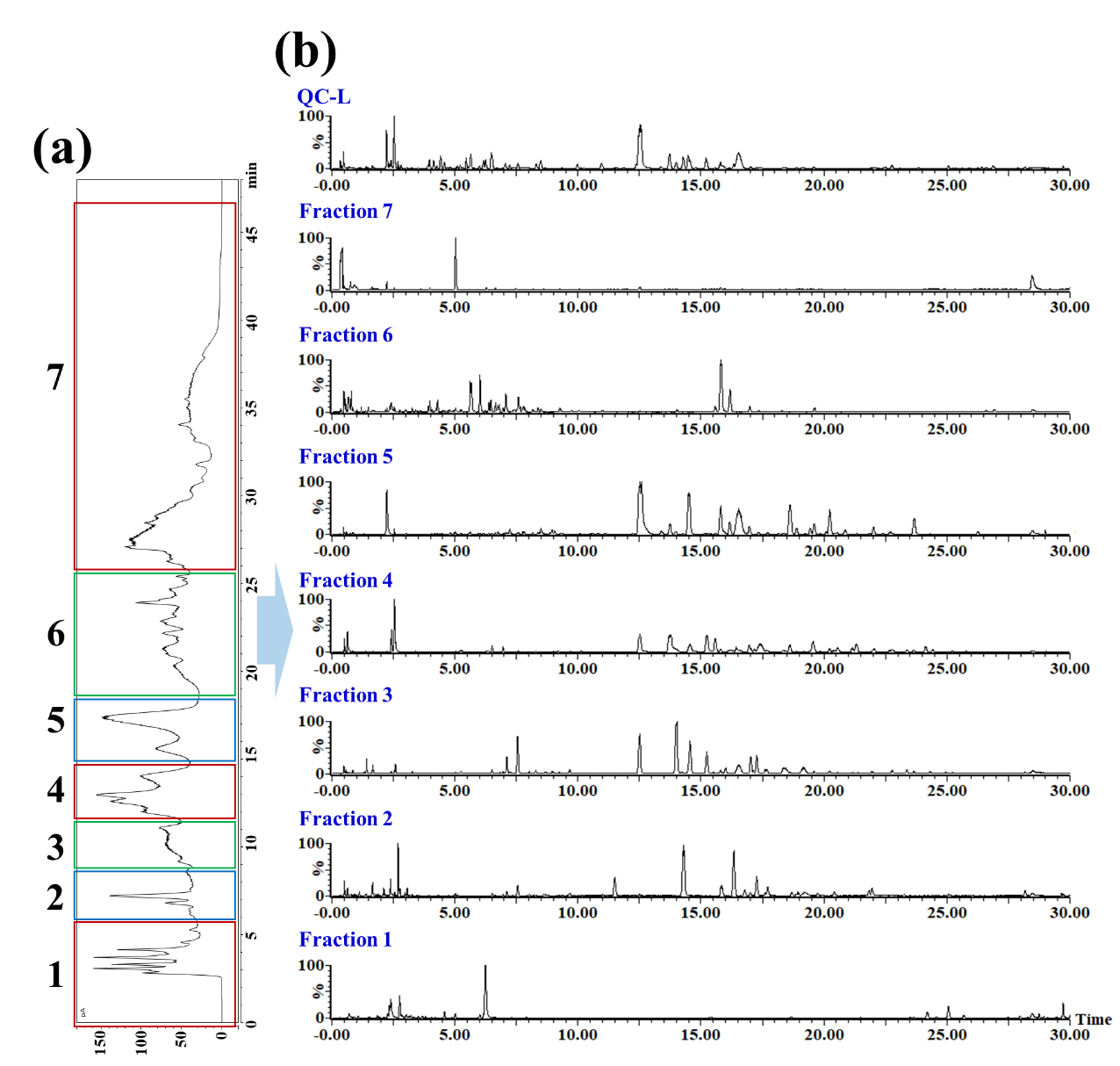
**

**FIGURE S6** Offline two-dimensional separation chromatogram of leaves of *G. longipes*: (A) chromatogram from one-dimensional HILIC column and (B) chromatogram from two-dimensional RP C18 column.

**Table S1** Structure list of gypenosides in the Herba Gynostemma mass spectrometry database.

| No. | Name | R | R1 | R2 | R3 | R4 |
| --- | --- | --- | --- | --- | --- | --- |
| 1 | (20R,21S,23R)-O-n-butyl-3β,20ξ-dihydroxy-21,23-epoxydammar-24-ene-3-O-α-L-rhamnopyranosyl (1→2)-[β-D-xylopyranosyl (1→3)]-6-O-acetyl-β-D-glucopyranoside | B-1 | S5 | C_4_H_9_ | n | n |
| 2 | (20S,21R,23R)-O-n-butyl-3β,20ξ-dihydroxy-21,23-epoxydammar-24-ene 3-O-α-L-rhamnopyranosyl (1→2)-[β-D-xylopyranosyl (1→3)]-β-D-arabinopyranoside | B-1 | S1 | C_4_H_9_ | n | n |
| 3 | (3β,20S,23R)-3,20,23,26-tetrahydroxydammar-24-en-21-oic acid-21,23-lactone 3-O-[α-L-rhamnopyranosyl-(1→ 2)]-[β-D-xylopyranosyl-(1→ 3)]- β-D-glucopyranoside | C-3 | S4 | n | n | n |
| 4 | (3β,20S,23R)-3,20,23-trihydroxydammar-24-en-21-oic acid-21,23-lactone 3-O-[α-L-rhamnopyranosyl-(1→ 2)]-[β-D-xylopyranosyl-(1→ 3)]-α-L-arabinopyranoside | C-2 | S1 | n | n | n |
| 5 | (3β,20S,23R)-3,20,23-trihydroxydammar-24-en-21-oic acid-21,23-lactone 3-O-[α-L-rhamnopyranosyl-(1→2)]-[β-D-xylopyranosyl-(1→3)]-β-D-glucopyranoside | C-1 | S4 | H | n | n |
| 6 | (3β,20S,23S)-3,20,23-trihydroxydammar-24-en-21-oic acid-21,23-lactone 3-O-[α-L-rhamnopyranosyl-(1→ 2)]-[β-D-xylopyranosyl-(1→3)]-6-O-acetyl-β-D-glucopyranoside | C-1 | S17 | H | n | n |
| 7 | (20R)-3β,20,23ξ-trihydroxydammar-24-en-21-oic acid-21,23-lactone-3-O[(β-D-glucopyranosyl-(1→2)-α-L-arabinopyranosyl]-20-O-β-D-rhamnopyranoside | C-1 | S16 | Rha | n | n |
| 8 | (20R)-dammar-25-ene-3β,20,21,24[-tetraol-3-O-[(β-D-glucopyranosyl-( 1→2)-α-L-arabinopyranosyl]-21 -O-β-D-glucopyranosy1-24-O-rhamnopyranoside | K-5 | S16 | H | Glc | Rha |
| 9 | (20R,21R23R,24R)-3β,19,20,21,23- pentahydroxy-21,24-cyclodrammar-25-ene-3-O-[α-L-rhamnopyranosyl(1 →2)][β-D-xylopyranosyl(1→3)]-β-D-glucopyranside | A-2 | S4 | n | n | n |
| 10 | (20R,23R)-3β,20-dihydroxy-19-oxodammar-24-en-21-oic acid-21,23-lactone 3-O-[α-L-rhamnopyranosyl-(1→2)] [β-D-xylopyranosyl-(1→3)]-α-L-arabinopyranoside | C-2 | S1 | n | n | n |
| 11 | (20R,23R)-3β,20-dihydroxyldammarane-24-ene-21-oic acid-21, 23-lactone | C-1 | H | H | n | n |
| 12 | (20R,24R )-3β,21,25-trihydroxy-20,24-epoxydrammar-3-O-{[α-L-rhamnopyranosy l( 1→2)][β-D-xylopyranosyl(1→3)]-α-L-glucopyranosyl}-21-O-β-D-glucopyranoside | D-1 | S4 | Glc | n | n |
| 13 | (20S)-3β,20, 21-trihy droxydammar-24-ene-19-oxo-3-O-[α-L-rhamnopyranosyl-(1→2)][β-D-xylopyranosyl(1→3)]-α-L-arabinopyranosyl-21-O-α-L-rhamnopyranosyl-(1 →6)-β-D-glucopyranoside | F-2 | S1 | H | S8 | n |
| 14 | (20S)-3β,20,21-trihydroxy-19-oxo-dammara-23,25-diene 3-O-{[α-L-rhamnopyranosyl-(1→2)][β-D-xylopyranosyl-(1→3)]-α-L-arabinopyranosyl}-21-O-β-D-glucopyranoside | N-2 | S1 | Glc | n | n |
| 15 | (20S)-3β,20,21-trihydroxydammar-19-oxo- 24-ene-3-O-{[α-L-rhamnopyranosyl(1→2)]-[β-D-xylopyranosyl(1→3)]-α- L-arabinopyranosyl}-21-O-β-D-glucopyranosyl(1→6)-β-D-glucopyranoside | F-1 | S1 | H | S8 | n |
| 16 | (20S)-3β,20,21-trihydroxydammar-24-ene-3-O-{[α-L-rhamnopyranosyl(1→2)][β-D-glucopyranosyl(1→3)]-β-D-glucopyranosyl}-21-O-[β-D-glucopyranosyl(1→6)]-β-D-glucopyranoside | F-3 | S3 | H | S8 | n |
| 17 | (20S)-3β,20,21-trihydroxy-dammara-23,25-diene 3-O-[α-L-rhamnopyranosyl-(1→2)] [β-D-xylopyranosyl-(1→3)]-β-D-glucopyranosyl-21-O-β-D-glucopyranoside | N-3 | S4 | Glc | n | n |
| 18 | (20S)-3β,20,23ξ-trihydroxydammar-24-en-21 -oic acid-21,23-lactone-3-O[(β-D-glucopyranosyl-(1→2)-α-L-arabinopyranosyl]-20-O-β-D-rhamnopyranoside | C-1 | S16 | Rha | n | n |
| 19 | (20S)-3β,20,29-trihydroxydammar-24-en-21-carboxylic acid-3-O-{[α-Lrhamnopyranosyl (1→2)][α-L-rhamnopyranosyl(1→6)-b- D-glucopyranosyl(1→3)]-α-L-arabinopyranosyl}-21-O-b- D-glucopyranoside | O-1 | S36 | Glc | n | n |
| 20 | (20S)-3β,20-dihydroxydammar-24-en-21,29-dioic acid-3-O-[α-L-arabinopyranosyl]-21-O-β-D-glucopyranoside | O-2 | Ara | Glc | n | n |
| 21 | (20S)-3β,20-dihydroxydammar-24-en-21,29-dioic acid-3-O-{[α-L-rhamnopyranosyl(1→6)-β-D-glucopyranosyl(1→3)]-α-L-arabinopyranosyl}-21-O-β- D-glucopyranoside | O-2 | S37 | Glc | n | n |
| 22 | (20S)-3β,20-dihydroxydammar-24-en-21,29-dioic acid-3-O-{[β-D-glucopyranosyl(1→3)]-α-L-arabinopyranosyl}-21-O-β-D-glucopyranoside | O-2 | S38 | Glc | n | n |
| 23 | (20S)-3β,20-dihydroxydammar-24-en-21,29-dioic acid-21-O-[β-D-glucopyranoside(1→2)][α-L-rhamnopyranosyl(1→6)]-β-D-glucopyranoside | O-2 | H | S28 | n | n |
| 24 | (20S)-dammar-23-ene- 3β-20,25,26-tetraol-3-O-[(β-D-glucopyranosyl-(1→2)-α-L-arabinopyranosyl]-20-O-β- D-rhamnopyranosyl-26-O-glucopyranos | J-10 | S11 | Rha | Glc | n |
| 25 | (20S)-dammarane-24(25)-ene-3β,20,21-tetrol | F-3 | H | H | H | n |
| 26 | (20S, 23R)-3β,20β-dihydroxydamma-24-dien-21-oic acid 21,23-lactone | C-1 | H | H | n | n |
| 27 | (20S, 24S)-20,24-epoxydammarane-3β,12β,25-triol | D-2 | H | n | n | n |
| 28 | (20S,23S)-3β,20-dihydroxyldammarane-24-ene-21-oic acid-21, 23-lactone | C-1 | H | H | n | n |
| 29 | (20S,23ξ,24ξ)-3β,20,21,23,24- pentahydroxy-3-O-[α-L-rhamnopyranosyl(1→2)][β-D-xylopyranosyl(1→3) ]-α-L-arabinopyranoside | G-2 | S1 | n | n | n |
| 30 | (20S,24S)-dammarane-25(26)-ene-3β,12β,20,24-tetrol | K-1 | H | H | n | n |
| 31 | (23S)-3β-dihydroxyl-dammarane-24-ene-21-oic acid-21, 23-lactone | R-1 | H | n | n | n |
| 32 | (23S)-21β-O-methyl-3β,20ξ-dihydroxy-12-oxo-21,23-epoxydammar-24-ene-3-O-[α-L-rhamnopyranosyl(1→2)][β-D-glucopyranosyl (1→3)]-α-L-arabinopyranoside | B-2 | S2 | CH_3_ | n | n |
| 33 | (23ξ)-21ξ-O-n-butyl-3β,20ξ,21ξ-trihydroxy-19-oxo-21,23-epoxydammar-24-ene 3-O-[α-L-Rhamnopyranosyl-(1→ 2)]-[β-D-xylopyranosyl-(1→ 3)]-α-L-arabinopyranoside | B-3 | S1 | C_4_H_9_ | n | n |
| 34 | 1R,3S,20S-1,3-epoxy-20,25-epoxy-dammar | QT-3 | Glc | n | n | n |
| 35 | 2α,3β,12β,20S-tetrahydroxydammar-24-ene-3-O-[β-D-glucopyranosyl(1→4)-β-D-glucopyranosyl]-20-O-[β-D-xylopyranosyl-(1→6)-β-D-glucopyranoside] | I-1 | S23 | S10 | n | n |
| 36 | 2α,3β,12β,20S-tetrahydroxydammar-24-ene-3-O-β-D-glucopyranosyl-20-acetylglucopyranosyl-glucopyranoside | I-1 | Glc | S24 | n | n |
| 37 | 3-O-β-D-glucopyranosyl-(1→6)-β-D-glucopyranosyl-12β-hydroxy-25-hydroperoxy dammaran-23,24(E)-ene-20(S)-yl 0-β-D-xylopyranosyl-(1→6)-β-D-glucopyranoside | J-1 | S11 | S10 | n | n |
| 38 | 3-O-β-D-glucopyranosyl-2α-hydroxy-24-en-dammaran-20(S)-yl O-β-D-xylopyranosyl-(1→6)-β-D-glucopyranoside | I-4 | Glc | S10 | n | n |
| 39 | 3-O-β-D-glucopyranosyl-gypensapogenin D | R-1 | Glc | n | n | n |
| 40 | 3S,20S,23R-3,20,23-Trihydroxydammar-24-en-21-oic Acid-21,23-lactone 3-O-β-D-Glucopyranoside | C-1 | Glc | H | n | n |
| 41 | 3S,20S,23R-3,20,23-Trihydroxydammar-24-en-21-oic Acid-21,23-lactone-3-β-D-xylopyranosyl-(1→3)-6-O-acetyl-β-D-glucopyranosyl | C-1 | S18 | H | n | n |
| 42 | 3S,20S,23R-3,20,23-Trihydroxydammar-24-en-21-oic Acid-21,23-lactone-3-α-L-rhamnopyranosyl-(1→ 2)-β-D-glucopyranosyl | C-1 | S19 | H | n | n |
| 43 | 3S,20S,23S-3,20,23-Trihydroxydammar-24-en-21-oic Acid-21,23-lactone 3-O-β-D-Glucopyranoside | C-1 | Glc | H | n | n |
| 44 | 3S,20S,23S-3,20,23-Trihydroxydammar-24-en-21-oic Acid-21,23-lactoneβ-D-glucopyranoside | C-1 | S19 | H | n | n |
| 45 | 3S,20S,23S-19-Oxo-3,20,23-trihydroxydammar-24-en-21-oicAcid-21,23-lactone 3-O-α-L-Arabinopyranoside | C-2 | Ara | n | n | n |
| 46 | 3β, 19,20(S)-dihydroxydammar-24-ene-3-O-[β-D-glucopyranosyl(2→1)-β-D-glucopyranosyl]-20-O-[α-L-rhamnopyranosyl(6→1)-β-D-glucopyranoside | I-6 | S11 | S7 | n | n |
| 47 | 3β, 20S-dihydroxydammar-24-en-21-carboxylic acid 3-O-[α-L-rhamnopyranosyl(1→2)]-β-D-xylopyranosyl(1→3)-β-D-glucopyranoside-21-O-[β-D-glucopyranoside(1→2)][α-L-rhamnopyranosyl(1→6)]-β-D-glucopyranoside | O-3 | S4 | S28 | n | n |
| 48 | 3β,12β,20S,21-tetrahydroxydammar-24-ene 3-O-{[α-L-rhamnopyranosyl(1→2)][β-D-xylopyranosyl(1→3)]-6-O-acetyl-β-D-glucopyranosyl}-21-O-β- D-glucopyranoside | F-4 | S5 | Glc | n | n |
| 49 | 3β,12β,20S-trihydroxydammar-24-ene-3-O-β-D-xylopyranosyl-20-O-[β-D-xylopyranosyl-(1→6)-β-Dglucopyranoside] | I-5 | Xyl | S10 | n | n |
| 50 | 3β,12β,23S,24R-tetrahydroxy-20S,25-epoxydammarane-3-O-β-D-glucopyranosyl (1→2)-β-D-xylopyranosyl | Q-1 | S21 | H | n | n |
| 51 | 3β,12β,23S,24R-tetrahydroxy-20S,25-epoxy dammarane-3-O-[β-D-xylopyranosyl(1→2)]-β-D-xylopyranosyl(1→6)-β-D-Glc | Q-1 | S44 | H | n | n |
| 52 | 3β,12β,23S,24R-tetrahydroxy-20S,25-epoxy dammarane-3-O-β-D-xylopyranosyl(1→2)-β-D-glucopyranosyl | Q-1 | S22 | H | n | n |
| 53 | 3β,12β,23S,24R-tetrahydroxy-20S,25-epoxydammarane-3-O-[β-D-glucopyranosyl (1→2)]-β-D-xylopyranosyl(1→6)-β-D-glucopyranosyl | Q-1 | S45 | H | n | n |
| 54 | 3β,12β,23S,25-tetrahydroxy-20S,24S-epoxydammarane-3-O-[β-D-xylopyranosyl (1→2)]-β-D-glucopyranoside | D-4 | S22 | n | n | n |
| 55 | 3β,19,20(S)-trihydroxydammar-24-ene-3-O-[β-D-glucopyranosyl(1→2)-β-D-glucopyranosyl]-20-O-β-D-glucopyranoside | I-6 | S11 | Glc | n | n |
| 56 | 3β,20-dihydroxydammar-23,25-diene-21-carboxylic acid 3-O-[α-L-rhamnopyranosyl-(1→ 2)]-[β-D-xylopyranosyl-(1→3)]-β-D-glucopyranoside | N-4 | S4 | H | n | n |
| 57 | 3β,20-dihydroxydammar-23,25-diene-21-carboxylic acid 3-O-[α-L-rhamnopyranosyl-(1→2)]-[β-D-xylopyranosyl-(1→ 3)]-β-D-6-O-acetylglucopyranoside | N-4 | S5 | H | n | n |
| 58 | 111.3β,20S,21,25-tetrahydroxydammar-23-ene-3-O-{[α-L-rhamnopyranosyl(1→ 2)][β- D-glucopyranosyl(1→ 3)]-β-D-glucopyranosyl}-21-O-β-D-glucopyranoside | J-5 | S3 | Glc | n | n |
| 59 | 112.3β,20S,21-trihydroxy-25-methoxydammar-23-ene 3-O-α-L-rhamnopyranosyl(1→2)-[β-D-xylopyranosyl(1→3)]-β-D-glucopyranosyl-21-O-β-D-xylopyranoside | J-6 | S4 | H | Xyl | n |
| 60 | 3β,20S,21-trihydroxydammar-24-ene 3-O-[α-L-rhamnopyranosyl(1→ 2)][β-D-glucopyranosyl (1→ 3)]β-D-glucopyranoside | F-3 | S3 | H | H | n |
| 61 | 3β,20S,21-trihydroxydammar-24-ene 3-O-{[α-L-rhamnopyranosyl(1→ 2)][β-D-xylopyranosyl(1→ 3)]-β-D-glucopyranosyl}-20-O-[β-D-xylopyranosyl(1→ 6)]-β-D-glucopyranoside | F-3 | S4 | S10 | H | n |
| 62 | 3β,20S,21-trihydroxydammar-24-ene 3-O-{[α-L-rhamnopyranosyl(1→2)][β-D-glucopyranosyl (1→3)]-α-L-arabinopyranosyl}-21-O-β- D-glucopyranoside | O-3 | S2 | Glc | n | n |
| 63 | 3β,20S,21-trihydroxydammar-24-ene 3-3-O-[α-L- rhamnopyranosyl(1→2)][β-D-xylopyranosyl (1→3)]-6-O-acetyl-β-D-glucopyranosyl-21-O-β- D-glucopyranoside | F-3 | S5 | H | Glc | n |
| 64 | 3β,20S,21-trihydroxydammar-24-ene-3-O-{[α- L-rhamnopyranosyl(1→ 2)][β-D-glucopyranosyl (1→ 3)] [β-D-xylopyranosyl(1→ 6)]-β-D-glucopyranosyl}-20-O-β-D-glucopyranoside | F-3 | S9 | Glc | H | n |
| 65 | 3β,20S,21-trihydroxydammar-24-ene-3-O-[α-L-rhamnopyranosyl(1→2)]-β-D-glucopyranosyl (1→3)-β-D-glucopyranosyl-21-O-[β-D-glucopyranosyl | F-3 | S3 | H | Glc | n |
| 66 | 3β,20S,23R-3,20,23-trihydroxydammar-24-en-21-oic acid-21,23-lactone 3-O-α-L-rhamnopyranosyl-1→2-β-D-xylopyranosyl-1→3-β-D-6- | C-1 | S5 | H | n | n |
| 67 | 124.3β,20S,25-trihydroxydammar-23-en-21,28-dioic acid-3-O-{[α-L-rhamnopyranosyl(1→2)][α-L-rhamnopyranosyl(1→6)-β- D-glucopyranosyl (1→3)]-α-L-arabinopyranosyl}-21-O-β-D-glucopyranoside | J-7 | S36 | H | Glc | n |
| 68 | 3β,20S,29-trihydroxydammar-24-en-21-carboxylic acid 3-O-[α-L-rhamnopyranosyl (1→2)]-β-D-xylopyranosyl(1→3)-β-D-glucopyranoside-21-O-[β-D-glucopyranoside (1→2)][α-L-rhamnopyranosyl(1→6)]-β-D-glucopyranoside | O-1 | S4 | S28 | n | n |
| 69 | 3β,20S,29-trihydroxydammar-24-en-21-carboxylic acid 3-O-[α-L-rhamnopyranosyl (1→2)]-α-L-rhamnopyranosyl(1→3)-β-D-glucopyranoside-21-O-β-D-glucopyranoside (1→2)-β-D-glucopyranoside | O-1 | S27 | S11 | n | n |
| 70 | 3β,20S,29-trihydroxydammar-24-en-21-carboxylicacid 3-O-[α-L-rhamnopyranosyl (1→2)]-α-L-rhamnopyranosyl(1→3)-β-D-glucopyranoside-21-O-[β-D-glucopyranoside (1→2)][α-L-rhamnopyranosyl(1→6)]-β-D-glucopyranoside | O-1 | S27 | S28 | n | n |
| 71 | 3β,20S-dihydroxydammar-24-en-21-carboxylic acid-3-O-[α-L-rhamnopyranosyl(1→2)]-β-D-glucopyranoside(1→3)-β-D-glucopyranoside-21-O-β-D-glucopyranoside(1→2)-β-D-glucopyranoside | O-3 | S3 | S11 | n | n |
| 72 | 3β,20S-dihydroxydammar-24-en-21,28-dioic acid 3-O- {[α-L-rhamnopyranosyl(1→2)][β-D-glucopyranosyl(1→3)]-α-L-arabinopyranosyl}-21-O-β-D-glucopyranoside | O-2 | S2 | Glc | n | n |
| 73 | 3β,20S-dihydroxydammar-24-en-21,28-dioic acid 3-O-{[α-L-rhamnopyranosyl(1→2)][α-L-rhamnopyranosyl(1→6)-β-D-glucopyranosyl-(13)]-α-L-arabinopyranosyl}-21-O-β-D-glucopyranoside | O-2 | S36 | Glc | n | n |
| 74 | 3β,20S-dihydroxydammar-24-en-21,28-dioicacid-3-O-[β-D-glucopyranosyl(1→3)-α-L-arabinopyranosyl]-21-O- β-D-glucopyranoside | O-2 | S38 | Glc | n | n |
| 75 | 3β,20S-dihydroxydammar-24-en-21-carboxylic acid-3-O-[α-L-rhamnopyranosyl(1→2)]-β-D-glucopyranoside(1→3)-β-D-glucopyranoside-21-O-β-D-glucopyranoside(1→2)[α-L-rhamnopyranosyl(1→6)]-β-D-glucopyranoside | O-3 | S3 | S28 | n | n |
| 76 | 3β,20S-dihydroxydammar-24-en-21-carboxylic acid 3-O-[α-L-rhamnopyranosyl(1→2)]-α-L-rhamnopyranosyl(1→3)-β-D-glucopyranoside-21-O-β-D-glucopyranoside(1→2)[α-L-rhamnopyranosyl(1→6)]-β-D-glucopyranoside | O-3 | S27 | S28 | n | n |
| 77 | 3β,20S-dihydroxydammar-24-ene- 21-carboxylic acid 3-O-{[α-L-rhamnopyranosyl(1→ 2)] [β-D-glucopyranosyl(1→ 3)]β-D-glucopyranosyl}-21-O-β-D-glucopyranoside | O-3 | S3 | Glc | n | n |
| 78 | 3β,20S-dihydroxydammar-24-ene-3-O-β-D-glucopyranosyl-20-O-[β-D-xylopyranosyl-(1→6)-β-D-glucopyranoside] | I-7 | Glc | S10 | n | n |
| 79 | 3β,20S-dihydroxydammar-24-ene-21-carboxylic acid 3-O-[α-L-rhamnopyranosyl(1→ 2)] [β-D-glucopyranosyl(1→ 3)]-β-D-glucopyranoside | O-3 | S3 | H | n | n |
| 80 | 3β,20S-dihydroxydammar-24-ene-21-carboxylic acid 3-O-{[α-L-rhamnopyranosyl(1→ 2)] [β-D-xylopyranosyl(1→ 3)][β-D-glucopyranosyl(1→ 6)]β-D-glucopyranosyl}-21-O-β-D-glucopyranoside | O-3 | S39 | Glc | n | n |
| 81 | 3β,20S-dihydroxydammar-24-ene-21-carboxylic acid 3-O-{[α-L-rhamnopyranosyl(1→ 2)][β-D-glucopyranosyl(1→ 6)-β-D-glucopyranosyl(1→ 3)]β-D-glucopyranosyl}-21-O-β-D-glucopyranoside | O-3 | S40 | Glc | n | n |
| 82 | 6’’-Malonylginsenoside Rb1 | I-5 | S25 | S8 | n | n |
| 83 | 6’’-Malonylginsenoside Rd | I-5 | S25 | Glc | n | n |
| 84 | 6’’-Malonylginsenoside V | I-5 | S25 | S7 | n | n |
| 85 | 12-oxo-2α,3β,20(S)-trihydroxydammar-24-ene-3-O-[β-Dglucopyranosyl(1→2)-β-D-glucopyranosyl]-20-O-[α-L-rhamnopyranosyl (1→6)-β-D-glucopyranoside] | I-2 | S11 | S7 | n | n |
| 86 | 148.12-oxo-3β,20S,21,25-tetrahydroxydammar-23-ene-3-O- {[α-L-rhamnopyranosyl(1→ 2)] [β-D-glucopyranosyl(1→ 6)-β-D-glucopyranosyl (1→ 3)]-α-L-arabinopyranosyl}-21-O-β-D-glucopyranoside | J-8 | S42 | H | Glc | n |
| 87 | 12-oxo-3β,20S-dihydroxydammar-24-ene-3-O-[β-D-glucopyranosyl(1→2)-β-D-glucopyranosyl]-20-O-[β-D-xylopyranosyl-(1→6)-β-D-glucopyranoside] | I-8 | S11 | S10 | n | n |
| 88 | 19-oxo-3β,20(s)-dihydroxydammar-24-ene-3-O- [α-L-arabinopyranosyl(2→1)-β-D-glucopyranosyl]-20-O-β-D-glucopyranoside | I-9 | S16 | Glc | n | n |
| 89 | 19-oxo-3β,20(S)-dihydroxydammar-24-ene-3-O-[β-D-glucopyranosyl[2→1)-β-D-glucopyranosyl]-20-O-[α-L-rhamnopyranosyl [6→1)-β-D-glucopyranoside] | I-9 | S11 | S7 | n | n |
| 90 | 19-oxo-3β,20S,21,24S-tetrahydroxydammar-25-ene 3-O-α-L-rhamnopyranosyl(1→2)-[β-D-xylopyranosyl(1→3)]-α-L-arabinopyranosyl-21-O-β-D-glucopyranoside | K-6 | S1 | H | Glc | H |
| 91 | 19-oxo-3β,20S,21-trihydroxy-25-hydroperoxy dammar-23-ene-3-O-α-L-rhamnopyranosyl(1→2) -[β-D-xylopyranosyl(1→3)]-α-L-arabinopyranosyl | J-9 | S1 | H | H | n |
| 92 | 19-oxo-3β,20S,21-trihydroxy-25-hydroperoxy dammar-23-ene-3-O-α-L-rhamnopyranosyl (1→2)-[β-D-xylopyranosyl(1→3)]-α-L-arabinopyranosyl-21-O-β-D-glucopyranoside | J-9 | S1 | H | Glc | n |
| 93 | 19-oxo-20(S)-hydroxy-3-O-β-D-glucopyranosyl-dammer-24-ene | I-9 | Glc | H | n | n |
| 94 | 20(R)-ginsenoside Rg2 | I-10 | H | H | S19 | n |
| 95 | 20(R)-ginsenoside Rh1 | I-10 | H | H | Glc | n |
| 96 | 20(R)-Protopanaxadiol | I-5 | H | H | n | n |
| 97 | 20(S)-dammarane-25(26)-ene-3β,12β,20-triol | K-7 | H | H | n | n |
| 98 | 20(S)-ginsenoside Rg2 | I-10 | H | H | S19 | n |
| 99 | 20(S)-ginsenoside Rh1 | I-10 | H | H | Glc | n |
| 100 | 20(S)-protopanaxadiol | F-2 | H | H | H | n |
| 101 | (20R,21S,23S,24S)-3β,20,21,23-tetrahydroxy-19-oxo-21,24-cyclodammar-25-ene-3-O-[α-L-rhamnopyranosyl(1→2)][β-D-xylopyranosyl (1→3)] -α-L-arabinopyranoside | A-3 | S1 | n | n | n |
| 102 | 170.20R,21S,23S,24S-3β,20,21,23-tetrahydroxy-21,24-cyclodammar-25-en 3-O-β-D-glucopyranoside | A-1 | Glc | n | n | n |
| 103 | 171.20R,21S,23S,24S-3β,20,21,23-tetrahydroxy-21,24-cyclodammar-25,26-ene | A-1 | H | n | n | n |
| 104 | 20R-3β,20,21ξ,23ξ-Tetrahydroxy-21,24ξ-cyclodammar-25-ene-3-O-[α-L-rhamnopyranosyl (1 →2)][β-D-xylopyranosyl(1→3)]-6-O-acetyl-β-D-glucopyranside | A-1 | S5 | n | n | n |
| 105 | 20S,24S-3β,20,21β,23β,25-pentahydroxy-21,24-cyclodammarane | E-3 | H | n | n | n |
| 106 | 20S-3β,20,21ξ,23,25-pentahydroxy-21,24ξ-cyclo-dammarane-3-O-[α-L-rhamnopyranosyl (1→ 2)][β-D-xylopyranosyl(1→3)]-6-O-acetyl-β-D-glucopyranosyl | E-3 | S5 | n | n | n |
| 107 | 20S-dammar-24-en-2a,3β,12b,20-tetrol | I-1 | H | H | n | n |
| 108 | 20S-protopanaxadiol 3-O-{[α-L-rhamnopyranosyl (1→ 2)][β-D-glucopyranosyl(1→3)]-β-D-glucopyranosyl}-20-O-β-D-glucopyranoside | I-5 | S3 | Glc | n | n |
| 109 | 20S-protopanaxadiol 3-O-{[α-L-rhamnopyranosyl (1→ 2)][β-D-xylopyranosyl(1→ 3)]-6-O-acetyl-β-Dglucopyranosyl}-20-O-β-D-glucopyranoside | I-5 | S5 | Glc | n | n |
| 110 | 21-norgypenoside A | P-1 | S3 | n | n | n |
| 111 | 21-norgypenoside Aa | P-1 | S3 | n | n | n |
| 112 | 21-norgypenoside B | P-1 | S2 | n | n | n |
| 113 | 23(S)-3β,20ξ,21ξ-trihydroxy-19-oxo-21,23-epoxydammar-24-ene 3-O-α-L-rhamnopyranosyl (1→2)-[β-D-xylopyranosyl (1→3)]-β-D-arabinopyranoside | B-3 | S1 | H | n | n |
| 114 | 23(S)-21(R)-O-n-butyl-3β,20ξ-dihydroxy-21,23-epoxydammar-24-ene 3-O-α-L-rhamnopyranosyl (1→2)-[β-D-xylopyranosyl (1→3)]-β-D-arabinopyranoside | B-1 | S1 | C_4_H_9_ | n | n |
| 115 | 23-O-acetyl-3β,12β,23S,24R-tetrahydroxy-20S,25-epoxy dammarane-3-O-β-D-xylopyranosyl(1→2)-β-D-xylopyranosyl | Q-1 | S6 | CH_3_CO | n | n |
| 116 | 23-O-acetyl-3β,12β,23S,24R-tetrahydroxy-20S,25-epoxy dammarane-3-O-β-D-xylopyranosyl(1→2)-β-D-glucopyranosyl | Q-1 | S22 | CH_3_CO | n | n |
| 117 | 23-O-acetyl-3β,12β,23S,24R-tetrahydroxy-20S,25-epoxy dammarane-3-O-[β-D-xylopyranosyl(1→2)]-β-D-xylopyranosyl(1→6)-β-D-glucopyranosyl | Q-1 | S44 | CH_3_CO | n | n |
| 118 | 23S-3β,20ξ,21ξ-Trihydroxy-19-oxo-21,23-epoxydammar-24-ene-3-O-[α-L-rhamnopyranosyl -(1→ 2)]-[β-D-xylopyranosyl-(1→ 3)]-α-L-arabinopyranoside | B-4 | S1 | H | n | n |
| 119 | 23S-3β,20ξ,21ξ-Trihydroxy-21,23-epoxydammar-24-ene-3-O-α-L-rhamnopyranosyl (1→2)-[β-D-xylopyranosyl (1→3)]-β-D-glucopyranoside | B-1 | S4 | H | n | n |
| 120 | 23S-3β,20ξ,21ξ-Trihydroxy-21,23-epoxydammar-24-ene-3-O-α-L-rhamnopyranosyl (1→2)-[β-D-xylopyranosyl (1→3)]-6-O-acetyl-β-D-glucopyranoside | B-1 | S5 | H | n | n |
| 121 | 23(S)-21(R)-O-n-butyl-3β,20ξ-dihydroxy-21,23-epoxydammar-24-ene 3-O-α-L-rhamnopyranosyl (1→2)-[β-D-xylopyranosyl (1→3)]-β-D-glucopyranoside | B-1 | S4 | C_4_H_9_ | n | n |
| 122 | 23S-21R-O-n-butyl-3β,20ξ,21-trihydroxy-21,23-epoxydammar-24-ene-3-O-[α-L-rhamnopyranosyl -(1→ 2)]-[β-D-xylopyranosyl-(1→ 3)]-α-L-arabinopyranoside | B-1 | S1 | C_4_H_9_ | n | n |
| 123 | 23S-21R-O-n-butyl-19-oxo-3β,20ξ,21-trihydroxy-21,23-epoxydammar-24-ene-3-O-[α-L-rhamnopyranosyl-(1→ 2)]-[β-D-xylopyranosyl-(1→ 3)]-α-L-arabinopyranoside | B-3 | S1 | C_4_H_9_ | n | n |
| 124 | 23S-21S-O-n-butyl-3β,20ξ,21-trihydroxy-21,23-epoxydammar-24-ene-3-O-[α-L-rhamnopyranosyl -(1→ 2)]-[β-D-xylopyranosyl-(1→ 3)]-β-D-glucopyranoside | B-1 | S4 | C_4_H_9_ | n | n |
| 125 | 23S-21S-O-n-butyl-19-oxo-3β,20ξ,21-trihydroxy-21,23-epoxydammar-24-ene-3-O-[α-L-rhamnopyranosyl-(1→ 2)]-[β-D-xylopyranosyl-(1→ 3)]-α-L-arabinopyranoside | B-3 | S1 | C_4_H_9_ | n | n |
| 126 | 23S-21ξ-O-Ethyl-3β,20ξ,21-trihydroxy-21,23-epoxydammar-24-ene-3-O-[α-L-Rhamnopyranosyl-(1→ 2)]-[β-D-xylopyranosyl-(1→ 3)]-β-D-glucopyranoside | B-1 | S4 | C_2_H_5_ | n | n |
| 127 | 23β-H-3β,20ξ-dihydroxy-19-oxo-21,23-epoxy dammar-24-ene-3-O-[α-L-rhamnopyranosyl (1→ 2)][β-D-xylopyranosyl(1→3)]-α-L- arabinopyranoside | B-5 | S4 |  | n | n |
| 128 | compound K | I-5 | H | Glc | n | n |
| 129 | Dammara-20(22), 24-diene-3, 12-diol | M-2 | H | n | n | n |
| 130 | dammarane-(E)-20(22)-ene-3β,12β,25-triol | I-5 | H | H | n | n |
| 131 | damulin C | M-1 | S31 | n | n | n |
| 132 | damulin D | L-1 | S31 | n | n | n |
| 133 | damulin E | M-1 | Glc | n | n | n |
| 134 | damulin F | L-1 | Glc | n | n | n |
| 135 | ginsenoside F1 | I-10 | H | Glc | H | n |
| 136 | ginsenoside Rc | I-5 | S11 | S26 | n | n |
| 137 | ginsenoside Rg5 | I-5 | H | S10 | n | n |
| 138 | Gycomoside I | I-11 | H | S7 | n | n |
| 139 | Gycomoside II | I-12 | Glc | S8 | n | n |
| 140 | Gycomoside III | I-11 | H | Glc | n | n |
| 141 | Gycomoside IV | I-11 | Glc | S8 | n | n |
| 142 | gylongiposide II | B-1 | S16 | H | n | n |
| 143 | gylongiposide III | U-1 | S16 | n | n | n |
| 144 | gymnemaside VI | J-2 | H | S7 | n | n |
| 145 | gynosaponin I | I-5 | H | S19 | n | n |
| 146 | gynosaponin II | I-5 | H | S27 | n | n |
| 147 | gynosaponin IV | I-5 | Glc | S27 | n | n |
| 148 | Gynosaponin TN-2 | I-1 | H | S7 | n | n |
| 149 | gynosaponin V | I-5 | S11 | S14 | n | n |
| 150 | gynosaponin VI | I-5 | S11 | S27 | n | n |
| 151 | gynoside A | D-2 | S21 | n | n | n |
| 152 | gynoside B | D-2 | S11 | n | n | n |
| 153 | gynoside C | D-2 | S21 | n | n | n |
| 154 | gynoside D | D-4 | S21 | n | n | n |
| 155 | gypenbioside A | C-1 | S20 | H | n | n |
| 156 | gypenbioside B | C-1 | S20 | H | n | n |
| 157 | Gypenoside XXXI | F-2 | S11 | H | H | n |
| 158 | gypenoside 3 | F-3 | Glc | H | Glc | n |
| 159 | gypenoside 4 | F-1 | Ara | H | Glc | n |
| 160 | gypenoside 5 | K-6 | S1 | H | Glc | OH |
| 161 | Gypenoside C | K-1 | H | S29 | n | n |
| 162 | Gypenoside CI | K-1 | S11 | S29 | n | n |
| 163 | Gypenoside CP1 | I-1 | S24 | S10 | n | n |
| 164 | Gypenoside CP2 | I-1 | S46 | Glc | n | n |
| 165 | Gypenoside CP3 | I-1 | S46 | S10 | n | n |
| 166 | Gypenoside CP4 | I-5 | S46 | S10 | n | n |
| 167 | Gypenoside CP5 | I-1 | S47 | Glc | n | n |
| 168 | Gypenoside CP6 | I-1 | S47 | S10 | n | n |
| 169 | Gypenoside D | F-1 | S12 | H | H | n |
| 170 | gypenoside GC1 | K-3 | H | S7 | n | n |
| 171 | gypenoside GC2 | K-2 | H | S7 | n | n |
| 172 | gypenoside GC3 | K-4 | H | S7 | n | n |
| 173 | gypenoside GC4 | J-3 | H | S7 | n | n |
| 174 | gypenoside GC5 | K-2 | H | S8 | n | n |
| 175 | gypenoside GC6 | K-2 | Glc | S7 | n | n |
| 176 | gypenoside GC7 | J-2 | Glc | S7 | n | n |
| 177 | gypenoside GD2 | K-3 | S11 | S7 | n | n |
| 178 | gypenoside GD3 | J-3 | H | S7 | n | n |
| 179 | gypenoside GD4 | J-3 | S11 | S7 | n | n |
| 180 | Gypenoside I | I-5 | S28 | S8 | n | n |
| 181 | Gypenoside I-AH | I-5 | S28 | H | n | n |
| 182 | Gypenoside I-EH | I-5 | S7 | S8 | n | n |
| 183 | Gypenoside II | I-5 | S28 | S7 | n | n |
| 184 | Gypenoside III | I-5 | S11 | S8 | n | n |
| 185 | Gypenoside IV | I-5 | S11 | S10 | n | n |
| 186 | Gypenoside L-Em | I-1 | Glc | H | n | n |
| 187 | Gypenoside LI | I-1 | S11 | H | n | n |
| 188 | Gypenoside LII | F-5 | S14 | Glc | n | n |
| 189 | Gypenoside LIII | I-13 | S16 | H | n | n |
| 190 | Gypenoside LIV | I-6 | S16 | S10 | n | n |
| 191 | Gypenoside LIX | I-3 | H | S10 | n | n |
| 192 | Gypenoside LV | I-6 | Glc | S10 | n | n |
| 193 | Gypenoside LVIII | I-5 | S16 | S10 | n | n |
| 194 | Gypenoside LXII | I-14 | S11 | S10 | n | n |
| 195 | Gypenoside LXIII | I-6 | S11 | S10 | n | n |
| 196 | Gypenoside LXIV | I-14 | Glc | S10 | n | n |
| 197 | Gypenoside LXIX | J-11 | S11 | S10 | n | n |
| 198 | Gypenoside LXV | I-14 | H | S10 | n | n |
| 199 | Gypenoside LXVI | I-14 | H | S7 | n | n |
| 200 | Gypenoside LXVII | I-15 | S11 | S10 | n | n |
| 201 | Gypenoside LXX | I-16 | S11 | S10 | n | n |
| 202 | Gypenoside LXXI | K-1 | S11 | S10 | n | n |
| 203 | Gypenoside LXXII | I-14 | S7 | Glc | n | n |
| 204 | Gypenoside LXXIII | I-4 | Glc | S7 | n | n |
| 205 | Gypenoside LXXIV | I-1 | H | S8 | n | n |
| 206 | Gypenoside LXXIX | I-14 | Glc | Glc | n | n |
| 207 | Gypenoside LXXV | I-5 | H | S8 | n | n |
| 208 | Gypenoside LXXVI | I-14 | H | Glc | n | n |
| 209 | .Gypenoside LXXVIII | I-4 | H | S10 | n | n |
| 210 | Gypenoside LXXXIX | QT-1-2 | S11 | S29 | n | n |
| 211 | Gypenoside LXXXVIII | I-5 | Glc | S29 | n | n |
| 212 | gypenoside S1 | J-12 | S2 | H | Glc | n |
| 213 | gypenoside S2 | J-12 | S1 | H | Glc | n |
| 214 | gypenoside S3 | J-13 | S1 | H | Glc | n |
| 215 | gypenoside S4 | J-14 | S1 | H | Glc | n |
| 216 | Gypenoside V | I-5 | S11 | S7 | n | n |
| 217 | Gypenoside VI | I-5 | S28 | Glc | n | n |
| 218 | Gypenoside VII | I-5 | S7 | S7 | n | n |
| 219 | gypenoside VN1 | F-3 | S2 | H | S8 | n |
| 220 | gypenoside VN1a | F-3 | S2 | H | Glc | n |
| 221 | gypenoside VN2 | F-6 | S2 | Glc | n | n |
| 222 | gypenoside VN3 | F-6 | S15 | Glc | n | n |
| 223 | gypenoside VN4 | J-8 | S2 | H | Glc | n |
| 224 | gypenoside VN5 | E-4 | S2 | n | n | n |
| 225 | gypenoside VN6 | E-5 | S2 | n | n | n |
| 226 | gypenoside VN7 | B-6 | S2 | H | n | n |
| 227 | Gypenoside X | I-5 | Glc | S7 | n | n |
| 228 | Gypenoside XC | J-11 | S11 | S29 | n | n |
| 229 | Gypenoside XCI | I-5 | S21 | Glc | n | n |
| 230 | Gypenoside XCII | I-7 | S11 | S29 | n | n |
| 231 | Gypenoside XCIII | I-7 | S11 | S30 | n | n |
| 232 | Gypenoside XCIV | I-5 | S31 | S29 | n | n |
| 233 | Gypenoside XCIX | K-1 | H | S29 | n | n |
| 234 | Gypenoside XCV | I-7 | S31 | Glc | n | n |
| 235 | Gypenoside XCVI | I-5 | S30 | S29 | n | n |
| 236 | Gypenoside XCVII | I-1 | H | S26 | n | n |
| 237 | Gypenoside XCVIII | I-5 | S11 | S29 | n | n |
| 238 | Gypenoside XI | I-5 | S7 | Glc | n | n |
| 239 | Gypenoside XIII | I-5 | H | S10 | n | n |
| 240 | Gypenoside XIV | I-5 | H | S7 | n | n |
| 241 | Gypenoside XIX | I-16 | S11 | S7 | n | n |
| 242 | Gypenoside XL | I-13 | S11 | H | n | n |
| 243 | Gypenoside XLI | I-6 | S11 | H | n | n |
| 244 | Gypenoside XLIII | I-1 | S11 | S7 | n | n |
| 245 | Gypenoside XLIV | I-1 | Glc | S8 | n | n |
| 246 | Gypenoside XLV | I-1 | Glc | S7 | n | n |
| 247 | Gypenoside XLVII | I-3 | S11 | S7 | n | n |
| 248 | Gypenoside XLVIII | F-1 | S2 | H | Glc | n |
| 249 | Gypenoside XV | I-5 | S21 | S10 | n | n |
| 250 | Gypenoside XVI | I-5 | S21 | S7 | n | n |
| 251 | Gypenoside XVII | I-5 | Glc | S8 | n | n |
| 252 | Gypenoside XVIII | I-16 | S28 | S7 | n | n |
| 253 | Gypenoside XX | I-16 | S28 | S8 | n | n |
| 254 | Gypenoside XXI | I-16 | H | S10 | n | n |
| 255 | Gypenoside XXII | F-2 | S11 | S10 | H | n |
| 256 | Gypenoside XXIII | F-2 | S11 | H | Glc | n |
| 257 | Gypenoside XXIII-Em | F-2 | Glc | H | H | n |
| 258 | Gypenoside XXIV | F-1 | S11 | H | Glc | n |
| 259 | Gypenoside XXIV-Em | F-1 | Glc | H | H | n |
| 260 | Gypenoside XXIX | I-9 | S16 | H | n | n |
| 261 | Gypenoside XXV | F-1 | S16 | H | Glc | n |
| 262 | Gypenoside XXV-Em | F-1 | Ara | H | H | n |
| 263 | Gypenoside XXVI | F-1 | S16 | Glc | H | n |
| 264 | Gypenoside XXVII | I-6 | S11 | H | n | n |
| 265 | Gypenoside XXVII-Em | I-6 | Glc | H | n | n |
| 266 | Gypenoside XXVIII | I-9 | S11 | H | n | n |
| 267 | Gypenoside XXX | F-2 | Glc | Glc | H | n |
| 268 | Gypenoside XXXII | F-2 | Glc | H | Glc | n |
| 269 | Gypenoside XXXIII | F-1 | S11 | H | H | n |
| 270 | Gypenoside XXXIV | F-1 | S11 | S7 | H | n |
| 271 | Gypenoside XXXIX | I-14 | S11 | H | n | n |
| 272 | Gypenoside XXXV | F-1 | S11 | S10 | H | n |
| 273 | Gypenoside XXXVI | I-9 | S16 | S7 | n | n |
| 274 | Gypenoside XXXVII | I-9 | S16 | S10 | n | n |
| 275 | Gypenoside XXXVIII | I-14 | S11 | H | n | n |
| 276 | gypensapogenin A | QT-8 | n | n | n | n |
| 277 | gypensapogenin B | QT-8 | n | n | n | n |
| 278 | gypensapogenin C | R-1 | H | n | n | n |
| 279 | gypensapogenin D | R-1 | H | n | n | n |
| 280 | gypensapogenin E | S-1 | H | n | n | n |
| 281 | gypensapogenin F | S-1 | H | n | n | n |
| 282 | gypensapogenin G | S-1 | CH_3_CO | n | n | n |
| 283 | gypensapogenin H | QT-4-2 | Glc | n | n | n |
| 284 | gypensapogenin I | QT-4-3 | Glc | n | n | n |
| 285 | gypensapogenin L | QT-5 | Glc | n | n | n |
| 286 | gypensapogenin M | QT-6 | Glc | n | n | n |
| 287 | Gypensapogenin P | QT-4-1 | n | n | n | n |
| 288 | Gypensapogenin T | E-6 | Glc | n | n | n |
| 289 | Gypensapogenin W | N-4 | Glc | CH_3_ | n | n |
| 290 | Gypensapogenin X | A-4 | Glc | n | n | n |
| 291 | 23β-H-3β,20ξ-dihydroxy-12-oxo-21,23-epoxy dammar-24-ene-3-O-[α-L-rhamnopyranosyl (1→2)][β-D-glucopyranoside(1→3)]-α-L-arabinopyranoside | B-7 | S2 | n | n | n |
| 292 | longipenosides GL1 | G-3 | S43 | n | n | n |
| 293 | longipenosides GL3 | J-15 | S27 | H | n | n |
| 294 | longipenosides GL4 | J-15 | S43 | H | n | n |
| 295 | longipenosides GL5 | I-8 | S19 | H | n | n |
| 296 | Phanoside | B-1 | S15 | H | n | n |
| 297 | Yixinoside A | I-12 | S11 | S7 | n | n |
| 298 | Yixinoside B | Q-2 | H | n | n | n |
| 299 | yunnangypenoside A | K-8 | H | S11 | n | n |
| 300 | yunnangypenoside B | J-1 | H | S11 | n | n |
| 301 | Yunnangypenoside C | I-1 | H | S11 | n | n |
| 302 | Yunnangypenoside D | I-5 | S11 | S11 | n | n |
| 303 | Yunnangypenoside E | I-5 | S24 | S11 | n | n |
| 304 | Yunnangypenoside F | K-9 | S11 | S11 | n | n |
| 305 | Yunnangypenoside G | I-7 | Glc | S32 | n | n |
| 306 | Yunnangypenoside H | I-5 | S33 | S11 | n | n |
| 307 | Yunnangypenoside I | I-5 | Glc | S11 | n | n |
| 308 | Yunnangypenoside J | I-1 | Glc | S34 | n | n |
| 309 | Gymnemaside II | I-9 | S11 | Glc | n | n |
| 310 | (3β,20S)-3,19,20,21-tetrahydroxydammar-24-ene 3-O-[α-L-rhamnopyranosyl(1→2)]-[β-D-xylopyranosyl(1→3)]-α-L-arabinopyranosyl | F-2 | S1 | H | H | n |
| 311 | (20S,21ξ,24ξ)-3β,20,21,25-tetrahydroxy-20,24-cyclodammar-3-O-[α-L-rhamnopyranosyl (1→2)][β-D-xylopyranosyl(1→3)]-6-O-acetyl-β-D-glucopyranosyl | E-2 | S5 | H | n | n |
| 312 | (20S,21R,23R,24R)-3β, 20,21,25-tetrahydroxy-19-oxo-21,24-cyclodammar-3-O-[α-L-rhamnopyranosyl(1→2)][β-D-xylopyranosyl (1→3)]-α-L-arabinopyranoside | E-7 | S1 | n | n | n |
| 313 | 23ξ-21ξ-O-Ethyl-3β,20ξ,21-trihydroxy-21,23-epoxydammar-24-ene-3-O-[α-L-rhamnopyran-osyl-(1→ 2)]-[β-D-xylopyranosyl-(1→ 3)]-β-D-glucopyranoside | B-3 | S4 | C_2_H_5_ | n | n |
| 314 | (3β,20R,23R)-3-{{O-4-O-acetyl-6-deoxy-α-L-rhamannopyranosyl-(1→2)-O-[β-D-xylopyran-osyl-(1→3)]-6-O-acetyl-β-D-glucopyran-osyl}oxy}-20,23-dihydroxydammar-24-en-21-oic acid 21,23-lactone | C-1 | S17 | H | n | n |
| 315 | (20S)-3β,20,29-trihydroxydammar-24-en-21-carboxylic acid-3-O-{[α-L-rhamnopyranosyl (1→2)]{[β-D-glucopyranosyl(1→2)]-[α-L-rhamnopyranosyl(1→6)]-β-D-glucopyranosyl(1→ 3)}-β-D-glucopyranosyl}-21-O-β-D-glucopyranoside | O-1 | S41 | Glc | n | n |
| 316 | (20S)-3β,20-dihydroxydammar-24-en-29-aldehyde-21-carboxylic acid-3-O-{[α-L- rhamnopyranosyl(1→2)]{[β-D-glucopyranosyl (1→2)][α-L- rhamnopyranosyl(1→6)]-β-D-glucopyranosyl(1→3)}-α-L-arabinopyranosyl}-21-O-β-D-glucopyranoside | O-4 | S48 | Glc | n | n |
| 317 | Dammar-24-ene-1,3,12,20,26-pentol, (1β,3β,12β)- (9CI) | I-11 | H | H | n | n |
| 318 | Gycomoside III | I-12 | H | Glc | n | n |
| 319 | Gycomoside VN2 | I-12 | H | S35 | n | n |
| 320 | Gycomoside VN3 | I-12 | Glc | S35 | n | n |
| 321 | Gycomoside VN4 | I-12 | H | S30 | n | n |
| 322 | Gycomoside VN6 | L-2 | Glc | n | n | n |
| 323 | (3S,19S,20S,23R)-3,19-oxa-19-methoxy-20,23-dihydroxydammar-21-oic acid-21,23-lactone-24-ene | QT-9 | n | n | n | n |
| 324 | (3S,19S,20S,23S)-3,19-oxa-19-methoxy-20,23-dihydroxydammar21-oic acid-21,23-lactone-24-ene | QT-9 | n | n | n | n |
| 325 | (3S,19S,23R)-3,19-oxa-19-methoxy-20,23-dihydroxydammar-21-oicacid-21,23-lactone-20,24-diene | QT-10 | n | n | n | n |
| 326 | (3S,20R,23S,23S,24R)-20,25-oxa3,20,23-trihydroxy-21,24-cyclodammar 3-O-β-D-glucopyranoside | T-1 | Glc | n | n | n |
| 327 | Gypenoside LVI | I-1 | S11 | S26 | n | n |
| 328 | Gypenoside CXVII | I-1 | Xyl | S26 | n | n |
| 329 | Gypenoside CXVIII | I-5 | Xyl | S7 | n | n |
| 330 | Gypenoside CXX | I-1 | H | S29 | n | n |
| 331 | Gypensapogenin Q | QT-7-1 | n | n | n | n |
| 332 | Gypensapogenin O | QT-7-2 | Glc | n | n | n |
| FJ-1 ^a^ | Gypenoside XLV-Ed | I-1 | Glc | Glc | n | n |
| FJ-10 ^a^ | 2α,3β,12β,20(S)-tetrahydroxy-24(S)-hydroperoxyldammar-25-ene-20-O-[β-D-xylopyranosyl(1→6)]-β-D-glucopyranoside | K-4 | H | S10 | n | n |
| FJ-11 ^a^ | 2α,3β,12β,20(S)-tetrahydroxy-25-hydroperoxyl dammar-23-ene-20-O-[β-D-xylopyranosyl (1→6)]-β-D-glucopyranoside | J-3 | H | S10 | n | n |
| FJ-12 ^a^ | 2α,3β,12β,20(S),24(S)-pentahydroxydammar-25-ene-20-O-[β-D-xylopyranosyl(1→6)]-β-D-glucopyranoside | K-2 | H | S10 | n | n |
| FJ-13 ^a^ | 3-O-[β-D-glucopyranosyl(1→2)]-β-D-glucopyranosyl-2α,3β,12β,20(S)-tetrahydroxy dammar-24-ene-20-O-β-D-glucopyranoside | I-1 | S11 | Glc | n | n |
| FJ-14 ^a^ | 2α,3β,12β,20(S),25-pentahydroxydammar-23-ene-20-O-[β-D-xylopyranosyl(1→6)]-β-D-glucopyranoside | J-2 | H | S10 | n | n |
| FJ-16 ^a^ | 2α,3β,12β,20(S)-tetrahydroxy-24(S)-hydroperoxyl dammar-25-ene-3-O-β-D-glucopyranoside | K-4 | Glc | H | n | n |
| FJ-17 ^a^ | 2α,3β,12β,20(S)-tetrahydroxy-24(S)hydroperoxyl dammar-25-ene-20-O-β-D-glucopyranoside | K-4 | H | Glc | n | n |
| FJ-18 ^a^ | 2*α*-OH-20(*S*)-ginsenoside Rh2 | I-1 | Glc | H | n | n |
| FJ-19 ^a^ | 2α,3β,12β,20(S)-tetrahydroxydammar-24-ene-20-O-β-D-glucopyranoside | I-1 | H | Glc | n | n |
| FJ-2 ^a^ | 2α,3β,12β,20(S)-tetrahydroxydammar-24-ene-20-O-[β-D-xylopyranosyl(1→6)]-β-D-glucopyranoside | I-1 | H | S10 | n | n |
| FJ-20 ^a^ | ginsenoside F2 | I-5 | Glc | Glc | n | n |
| FJ-22 ^a^ | 2α,3β,12β-trihydroxydammar-20(22),24-diene-3-O-[β-D-glucopyranosyl(1→2)]-β-D-glucopyranoside | M-1 | S11 | n | n | n |
| FJ-23 ^a^ | 3β,12β,20(S)-trihydroxydammar-24-ene-20-O-[β-D-xylopyranosyl(1→6)]-β-D-glucopyranoside | I-5 | H | S10 | n | n |
| FJ-24 ^a^ | 3β,12β,20(S)-trihydroxydammar-24-ene-3-O-[β-D-glucopyranosyl(1→2)]-β-D-glucopyranoside | I-5 | S11 | H | n | n |
| FJ-25 ^a^ | 2α,3β,12β,20(S)-tetrahydroxydammar-23,25-diene-20-O-β-D-glucopyranoside | N-1 | H | Glc | n | n |
| FJ-26 ^a^ | 2α,3β,12β-trihydroxydammar-20,24-diene-3-O-[β-D-glucopyranosyl(1→2)]-β-D-glucopyranoside | L-1 | S11 | n | n | n |
| FJ-27 ^a^ | 3-O-β-D-glucopyranosyl-3β,12β,20(S)-trihydroxy dammar-24-ene-20-O-[β-D-xylopyranosyl (1→6)]-β-D-glucopyranoside | I-5 | Glc | S10 | n | n |
| FJ-28 ^a^ | 2α,3β,12β,20(S)-tetrahydroxydammar-24-ene-3-O-[β-D-glucopyranosyl(1→2)]-β-D-glucopyranoside | I-1 | S11 | H | n | n |
| FJ-29 ^a^ | 2α,3β,12β,20(S),24(R)-pentahydroxydammar-25-ene-20-O-β-D-glucopyranoside | K-2 | H | Glc | n | n |
| FJ-3 ^a^ | 2α,3β,12β,20(S)-tetrahydroxy-25-hydroperoxyl dammar-23-ene-3-O-[β-D-glucopyranosyl (1→2)]-β-D-glucopyranoside | J-3 | S11 | H | n | n |
| FJ-30 ^a^ | 2α,3β,12β,20(S)-tetrahydroxy-24(R)hydroperoxyl dammar-25-ene-20-O-β-D-glucopyranoside | K-4 | H | Glc | n | n |
| FJ-31 ^a^ | 2α,3β,12β,20(S),25-pentahydroxydammar-23-ene-20-O-β-D-glucopyranoside | J-2 | H | Glc | n | n |
| FJ-32 ^a^ | 2α,3β,12β,20(S)-tetrahydroxy-25-hydroperoxyl dammar-23-ene-20-O-β-D-glucopyranoside | J-3 | H | Glc | n | n |
| FJ-33 ^a^ | 3β,12β,20(S)-trihydroxy-25-hydroperoxyl dammar-23-ene-20-O-[β-D-xylopyranosyl (1→6)]-β-D-glucopyranoside | J-1 | H | S10 | n | n |
| FJ-34 ^a^ | 12β,20(S),25-trihydroxydammar-3-keto-23-ene-20-O-[β-D-xylopyranosyl(1→6)]-β-D-glucopyranoside | QT-2 | n | S10 | n | n |
| FJ-35 ^a^ | 2α,3β,12β,20(S),26-pentahydroxydammar-24-ene-20-O-β-D-glucopyranoside | I-3 | H | Glc | n | n |
| FJ-36 ^a^ | 2α,3β,12β,20(S),24(S)-pentahydroxydammar-25-ene-20-O-β-D-glucopyranoside | K-2 | H | Glc | n | n |
| FJ-37 ^a^ | 3β,12β,20(S),24(S)-tetrahydroxydammar-25-ene-20-O-[β-D-xylopyranosyl(1→6)]-β-D-glucopyranoside | K-1 | H | S10 | n | n |
| FJ-4 ^a^ | 3-O-β-D-glucopyranosyl-2α,3β,12β,20(S),24(R)-pentahydroxydammar-25-ene-20-O-β-D-glucopyranoside | K-2 | Glc | Glc | n | n |
| FJ-41 ^a^ | Gypenoside LVII | I-1 | Glc | S10 | n | n |
| FJ-42 ^a^ | 3-O-β-D-glucopyranosyl2α,3β,12β,20(S)-tetrahydroxy-25-methoxydamar-24-ene-20-O-[β-D-xylopyranosyl(1→6)]-β-D-glucopyranoside | J-4 | S8 | Xyl | n | n |
| FJ-43 ^a^ | 3-O-[β-D-glucopyranosyl(1→2)]-β-D-glucopyranosyl-3β,20(S)-dihydroxydammar-24-ene-20-O-[β-D-xylopyranosyl(1→6)]-β-D-glucopyranoside | I-7 | S11 | S10 | n | n |
| FJ-48 ^a^ | 3-O-[β-D-glucopyranosyl(1→2)]-β-D-glucopyranosyl-3β,12β,20(S)-trihydroxydammar-24-ene-20-O-β-D-glucopyranoside | I-5 | S11 | Glc | n | n |
| FJ-49 ^a^ | 3-O-β-D-glucopyranosyl-2α,3β,12β,20(S)-tetrahydroxydammar-23,25-diene-20-O-[β-D-xylopyranosyl(1→6)]-β-D-glucopyranoside | N-1 | Glc | S10 | n | n |
| FJ-5 ^a^ | 3-O-β-D-glucopyranosyl-2α,3β,12β,20(S)-tetrahydroxy-24®-hydroperoxyldammar-25-ene-20-O-β-D-glucopyranoside | K-4 | Glc | Glc | n | n |
| FJ-50 ^a^ | 3-O-β-D-glucopyranosyl-2α,3β,12β,20(S),24(S)-pentahydroxydammar-25-ene-20-O-[β-D-xylopyranosyl(1→6)]-β-D-glucopyranoside | K-2 | Glc | S10 | n | n |
| FJ-52 ^a^ | 3-O-β-D-glucopyranosyl-2α,3β,20(S),25-trihydroxy-12β,23R-epoxydammar-24-ene-20-O-[β-D-xylopyranosyl(1→6)]-β-D-glucopyranoside | QT-1-1 | Glc | S10 | n | n |
| FJ-53 ^a^ | 3-β-D-glucopyranosyl-2α,3β,12β,20(S)-tetrahydroxy-25-hydroperoxyldammar-23-ene-20-O-[β-D-xylopyranosyl(1→6)]-β-D-glucopyranoside | J-3 | Glc | S10 | n | n |
| FJ-56 ^a^ | 3-O-[β-D-glucopyranosyl(1→2)]-β-D-glucopyranosyl-2α,3β,20(S)-trihydroxydammar-24-ene-20-O-[β-D-xylopyranosyl(1→6)]-β-D-glucopyranoside | I-4 | S11 | S10 | n | n |
| FJ-57 ^a^ | 3-O-β-D-glucopyranosyl-2α,3β,12β,20(S),24(R)-pentahydroxydammar-25-ene-20-O-[β-D-xylopyranosyl(1→6)]-β-D-glucopyranoside | K-2 | Glc | S10 | n | n |
| FJ-58 ^a^ | 3-O-[β-D-glucopyranosyl(1→2)]-β-D-glucopyranosyl-2α,3β,12β,20(S),25-pentahydroxydammar-23-ene-20-O-β-D-glucopyranoside | J-2 | S11 | Glc | n | n |
| FJ-59 ^a^ | 3-O-[β-D-glucopyranosyl(1→2)]-O-β-D-glucopyranosyl-2α,3β,12β,20(S),24(R)-pentahydroxydammar-25-ene-20-O-β-D-glucopyranoside | K-2 | S11 | Glc | n | n |
| FJ-6 ^a^ | 3-O-β-D-glucopyranosyl-2α,3β,12β,20(S)-tetrahydroxy-24(S)hydroperoxyldammar-25-ene-20-O-β-D-glucopyranoside | K-4 | Glc | Glc | n | n |
| FJ-61 ^a^ | 3-O-[β-D-glucopyranosyl(1→2)]-O-β-D-glucopyranosyl-2α,3β,12β,20(S)-tetrahydroxy-24(R)-hydroperoxyldammar-25-ene-20-O-β-D-glucopyranoside | K-4 | S11 | Glc | n | n |
| FJ-62 ^a^ | 3-O-[β-D-glucopyranosyl(1→2)]-β-D-glucopyranosyl-2α,3β,12β,20(S)-tetrahydroxy-25-hydroperoxyldammar-23-ene-20-O-β-D-glucopyranoside | J-3 | S11 | Glc | n | n |
| FJ-63 ^a^ | 3-O-[β-D-glucopyranosyl(1→2)]-β-D-glucopyranosyl-2α,3β,20(S)-trihydroxydammar-12-keto-24-ene-20-O-[β-D-xylopyranosyl(1→6)]-β-D-glucopyranoside | I-2 | S11 | S10 | n | n |
| FJ-64 ^a^ | 3-O-[β-D-glucopyranosyl(1→2)]-O-β-D-glucopyranosyl-2α,3β,12β,20(S),24(S)-pentahydroxydammar-25-ene-20-O-β-D-glucopyranoside | K-2 | S11 | Glc | n | n |
| FJ-65 ^a^ | 3-O-[β-D-glucopyranosyl(1→2)]-O-β-D-glucopyranosyl-2α,3β,12β,20(S)-tetrahydroxy-24(S)-hydroperoxyldammar-25-ene-20-O-β-D-glucopyranoside | K-4 | S11 | Glc | n | n |
| FJ-66 ^a^ | 3-O-[β-D-glucopyranosyl(1→2)]-β-D-glucopyranosyl-2α,3β,12β,20(S)-tetrahydroxydammar-24-ene-20-O-[β-D-xylopyranosyl(1→6)]-β-D-glucopyranoside | I-1 | S11 | S10 | n | n |
| FJ-67 ^a^ | 3-O-[β-D-glucopyranosyl(1→2)]-β-D-glucopyranosyl-2α,3β,12β,20(S),25-pentahydroxydammar-23-ene-20-O-[β-D-xylopyranosyl(1→6)]-β-D-glucopyranoside， | J-2 | S11 | S10 | n | n |
| FJ-68 ^a^ | 3-O-[β-D-glucopyranosyl(1→2)]-β-D-glucopyranosyl-2α,3β,12β,20(S)-tetrahydroxy-25-hydroperoxyldammar-23-ene-20-O-[β-D-xylopyranosyl(1→6)]-β-D-glucopyranoside | J-3 | S11 | S10 | n | n |
| FJ-69 ^a^ | 3-O-[β-D-glucopyranosyl(1→2)]-O-β-D-glucopyranosyl-2α,3β,12β,20(S),24(S)-pentahydroxydammar-25-ene-20-O-[β-D-xylopyranosyl(1→6)]-β-D-glucopyranoside | K-2 | S11 | S10 | n | n |
| FJ-7 ^a^ | 3-O-β-D-glucopyranosyl-2α,3β,12β,20(S),25-pentahydroxydammar-23-ene-20-O-β-D-glucopyranoside | J-2 | Glc | Glc | n | n |
| FJ-70 ^a^ | Gypenoside LXI | I-3 | S11 | S10 | n | n |
| FJ-71 ^a^ | 3-O-[β-D-glucopyranosyl(1→2)]-O-β-D-glucopyranosyl-2α,3β,12β,20(S)-tetrahydroxy-24(S)-hydroperoxyldammar-25-ene-20-O-[β-D-xylopyranosyl(1→6)]-β-D-glucopyranoside | K-4 | S11 | S10 | n | n |
| FJ-72 ^a^ | 3-O-[β-D-glucopyranosyl(1→2)]-O-β-D-glucopyranosyl-2α,3β,12β,20(S),24(R)-pentahydroxydammar-25-ene-20-O-[β-D-xylopyranosyl(1→6)]-β-D-glucopyranoside | K-2 | S11 | S10 | n | n |
| FJ-73 ^a^ | 3-O-β-D-glucopyranosyl-2α,3β,12β,20(S),25-pentahydroxydammar-23-ene-20-O-[β-D-xylopyranosyl(1→6)]-β-D-glucopyranoside | J-2 | Glc | S10 | n | n |
| FJ-74 ^a^ | gypenoside J3 | K-3 | S11 | S10 | n | n |
| FJ-75 ^a^ | 3-O-[β-D-glucopyranosyl(1→2)]-β-D-glucopyranosyl-2α,3β,12β,20(S),24,25-hexahydroxy dammar-20-O-[β-D-xylopyranosyl (1→6)]-β-D-glucopyranoside | G-1 | S11 | S10 | n | n |
| FJ-76 ^a^ | 3-O-[β-D-glucopyranosyl(1→2)]-β-D-glucopyranosyl-2α,3β,12β,20(S)-tetrahydroxy dammar-24-ene-20-O-[β-D-glucopyranosyl (1→6)]-β-D-glucopyranoside | I-1 | S11 | S8 | n | n |
| FJ-77 ^a^ | 3-O-[β-D-glucopyranosyl(1→2)]-O-β-D-glucopyranosyl-2α,3β,12β,20(S)-tetrahydroxy-24(R)-hydroperoxyldammar-25-ene-20-O-[β-D-xylopyranosyl(1→6)]-β-D-glucopyranoside | K-4 | S11 | S10 | n | n |
| FJ-8 ^a^ | 3-O-β-D-glucopyranosyl-2α,3β,12β,20(S),24(S)-pentahydroxydammar-25-ene-20-O-β-D-glucopyranoside | K-2 | Glc | Glc | n | n |
| FJ-9 ^a^ | 3-O-β-D-glucopyranosyl-2α,3β,12β,20(S)-tetrahydroxydammar-25-ene-24-keto-20-O-β-D-glucopyranoside | K-3 | Glc | Glc | n | n |
| GL-1 ^a^ | (3β,20R)-3,20,21ξ,23ξ-tetrahydroxy-21,24ξ-cyclodammar-25-ene 3-O-[α-L-rhamnopyranosyl (1→2)][β-D-xylopyranosyl(1→3)]-β-D-glucopyranoside | A-1 | S4 | n | n | n |
| GL-10 ^a^ | (3β,20S)-3,19,20,21-tetrahydroxydammar-24-ene 3-O-{[α-L-rhamnopyranosyl(1→2)]-[β-D-xylopyranosyl(1→3)]-α-L-arabinopyranosyl}-21-O-β-D-glucopyranoside | F-2 | S1 | H | Glc | n |
| GL-11 ^a^ | (3β,20S)-3,19,20,21-tetrahydroxydammar-24-ene 3-O-{[α-L-rhamnopyranosyl(1→2)]-[β-D-xylopyranosyl(1→3)]-β-D-glucopyranosyl}-21-O-β-D-glucopyranoside | F-2 | S4 | H | Glc | n |
| GL-12 ^a^ | (3β,20S,23S)-3,20,23-trihydroxydammar-24-en-21-oic acid-21,23-lactone 3-O-[α-L-rhamnopyran-osyl(1→2)]-[β-D-xylopyranosyl(1→3)]-α-L-arabinopyranoside | C-1 | S1 | H | n | n |
| GL-13 ^a^ | (3β,20R,23R)-3,20,23-trihydroxydammar-24-en-21-oic acid-21,23-lactone 3-O-[α-L-rhamnopyran-osyl(1→2)]-[β-D-xylopyranosyl(1→3)]-α-L-arabinopyranoside | C-1 | S1 | H | n | n |
| GL-14 ^a^ | (3β,20S,23S)-3,20,23-trihydroxydammar-24-en-21-oic acid 21,23-lactone 3-O-[α-L-rhamnopyran-osyl(1→2)]-[β-D-xylopyranosyl(1→3)]-6-O-acetyl-β-D-glucopyranoside | C-1 | S5 | H | n | n |
| GL-15 ^a^ | (3β,20R,23R)-3,20,23-trihydroxydammar-24-en-21-oic acid 21,23-lactone 3-O-[α-L-rhamnopyran-osyl(1→2)]-[β-D-xylopyranosyl(1→3)]-6-O-acetyl-β-D-glucopyranoside | C-1 | S5 | H | n | n |
| GL-17 ^a^ | (3β,20S,23S)-3,20,23-trihydroxydammar-24-en-21-oic acid 21,23-lactone 3-O-[α-L-rhamnopyran-osyl(1→2)]-[β-D-glucopyranosyl(1→3)]-β-D-glucopyranoside | C-1 | S3 | H | n | n |
| GL-18 ^a^ | (3β,20S)-3,20,21-trihydroxydammar-24-ene 3-O-[α-L-rhamnopyranosyl(1→2)]-[β-D-xylopyran-osyl(1→3)]-β-D-glucopyranoside | F-3 | S4 | H | H | n |
| GL-19 ^a^ | (3β,23S)-21ξ-O-ethyl-3,20ξ,21-trihydroxy-19-oxo-21,23-epoxydammar-24-ene 3-O-[α-L-rhamnopyranosyl(1→2)]-[β-D-xylopyranosyl (1→3)]-α-L-arabinopyranoside | B-3 | S1 | C_2_H_5_ | n | n |
| GL-2 ^a^ | (3β,20S)-19-oxo-3,20,21-trihydroxydammar-24-ene 3-O-β-D-xylopyranosyl(1→2)-β-D-xylopyranosyl-21-O-β-D-glucopyranoside | F-1 | S6 | H | Glc | n |
| GL-20 ^a^ | Gypenoside XLIX | F-1 | S1 | H | Glc | n |
| GL-21 ^a^ | Gypenoside A | B-3 | S1 | H | n | n |
| GL-3 ^a^ | (3β,20S,23S)-3,20,23-trihydroxydammar-24-en-21-oic acid 21,23-lactone 3-O-[α-L-rhamnopyran-osyl(1→2)]-[β-D-xylopyranosyl (1→3)]-β-D-glucopyranoside | C-1 | S4 | H | n | n |
| GL-4 ^a^ | (3β,20R,23R)-3,20,23-trihydroxydammar-24-en-21-oic acid 21,23-lactone 3-O-[α-L-rhamnopyran-osyl(1→2)]-[β-D-xylopyranosyl (1→3)]-β-D-glucopyranoside | C-1 | S4 | H | n | n |
| GL-5 ^a^ | (3β,23S)-21ξ-O-methyl-3,20ξ,21-trihydroxy-19-oxo-21,23-epoxydammar-24-ene-3-O-[α-L-rhamnopyranosyl(1→2)][β-D-xylopyranosyl (1→3)]-α-L-arabinopyranoside | B-3 | S1 | CH_3_ | n | n |
| GL-6 ^a^ | Gylongiposide I | F-1 | S1 | H | H | n |
| GL-7 ^a^ | (3β,20S)-3,20,21-trihydroxydammar-24-ene 3-O-{[α-L-rhamnopyranosyl(1→2)]-[β-D-xylopyran-osyl(1→3)]-β-D-glucopyranosyl}-21-O-β-D-glucopyranoside (Gypenoside C) | F-3 | S4 | H | Glc | n |
| GL-8 ^a^ | (3β,20S,23S)-19-oxo-3,20,23-trihydroxydammar-24-en-21-oci acid 21,23-lactone 3-O-[α-L-rhamnopyranosyl(1→2)]-[β-D-xylopyranosyl (1→3)]-α-L-arabinopyranoside | C-2 | S1 | n | n | n |
| JGL-1 ^a^ | (20R)-3β,20,21ξ,23ξ-tetrahydroxy-19-oxo-21,24ξ-cyclodammar-25-ene-3-O-[α-L-rhamnopyranosyl(1→2)][β-D-xylopyranosyl (1→3)]-α-L-arabinopyranoside | A-3 | S1 | n | n | n |
| JGL-10 ^a^ | (20S,21R,23R)-3β,20,21-trihydroxy-24-ene-epoxydrammar-3-O-{[α-L-rhamnopyranosyl (1→2)][β-D-xylopyranosyl(1→3)]-β-D-glucopyranosyl}-21-O-[β-D-glucopyranosyl (1→6)]-β-D-glucopyranoside | B-1 | S4 | S8 | n | n |
| JGL-11 ^a^ | (20S)-3β,19,20,21-terahydroxydammar-24-ene-3-O-{[α-L-rhamnopyranosyl(1→2)][β-D-xylopyranosyl(1→3)]-α-L-arabinopyranosyl}-21-O-[β-D-glucopyranosyl(1→6)]-β-D-glucopyranoside | F-2 | S1 | H | S7 | n |
| JGL-13 ^a^ | (20S)-3β,20,21-trihydroxydammar-24-ene-3-O-{[α-L-rhamnopyranosyl(1→2)][β-D-glucopyranosyl(1→3)]-β-D-glucopyranosyl}-21-O-[β-D-glucopyranosyl(1→6)]-β-D-glucopyranoside | F-3 | S3 | H | S7 | n |
| JGL-14 ^a^ | (20S,24S)-3β,21,25-trihydroxy-20,24-epoxydrammar-3-O-{[α-L-rhamnopyranosyl (1→2)][β-D-xylopyranosyl(1→3)]-α-L-glucopyranosyl}-21-O-β-D-glucopyranoside | D-1 | S4 | Glc | n | n |
| JGL-15 ^a^ | (20S,21S,24S)-3β,20,21,25-tetrahydroxy-21,24-cyclodammar-3-O-{[α-L-rhamnopyranosyl (1→2)][β-D-xylopyranosyl(1→3)]}-α-L-glucopyranosyl-25-O-β-D-glucopyranoside | E-2 | S4 | Glc | n | n |
| JGL-16 ^a^ | (20S,24S)-3β,21,25-trihydroxy-20,24-epoxy drammar-19-oxo-3-O-{[α-L-rhamnopyranosl(1→ 2)][β-D-xylopyranosyl(1→3)]-α-L-arabinpyran-osyl}-21-O-β-D-glucopyranoside | D-3 | S1 | Glc | n | n |
| JGL-17 ^a^ | (20S,21S,23S,24S)-3β,20,21,23-tetrahydroxy-19-oxo-21,24-cyclodammar-25-ene-3-O-[α-L-rhamnopyranosyl(1→2)][β-D-xylopyranosyl (1→3)]-α-L-arabinopyranoside | A-3 | S1 | n | n | n |
| JGL-18 ^a^ | (20ξ,21ξ,23ξ,24ξ)-3β,21,23-trihydroxy-19-oxo-21,24-cycly-20,25-epoxydrammar-3-O-[α-L-rhamnopyranosyl(1→2)][β-D-xylopyranosyl (1→3)]-α-L-arabinopyranoside | H-1 | S1 | n | n | n |
| JGL-19 ^a^ | (20S,21S,23S,24S)-3β,20,21,23,25-pentahydroxy-19-oxo-21,24-cyclodammar-3-O-[α-L-rhamnopyranosyl(1→2)][β-D-xylopyranosyl (1→3)]-α-L-arabinopyranoside | E-1 | S1 | n | n | n |
| JGL-2 ^a^ | (20S,24R)-3β,21,25-trihydroxy-20,24-epoxydrammar-3-O-{[α-L-rhamnopyranosyl (1→2)][β-D-xylopyranosyl(1→3)]-β-D-glucopyranosyl}-21-O-β-D-glucopyranoside | D-1 | S4 | Glc | n | n |
| JGL-20 ^a^ | (20S,21R,23R,24R)-pentahydroxy-19-oxo-21,24α-cyclodammar-3β-O-[α-L-rhamnopyran-osyl(1→2)][β-D-xylopyranosyl(1→3)]-α-L-arabinopyranoside | E-1 | S1 | n | n | n |
| JGL-3 ^a^ | 3β,21,25-trihydroxy-20S,24R-epoxydrammar-19-oxo-3-O-{[α-L-rhamnopyranosyl(1→2)][β-D-xylopyranosyl(1→3)]-α-L-arabinpyranosyl}-21-O-β-D-glucopyranoside | D-3 | S1 | Glc | n | n |
| JGL-4 ^a^ | (21S,23S)-3β,20ξ,21,26-tetrahydroxy-19-oxo-21,23-epoxydammar-24-ene-3-O-[α-L-rhamnopy-ranosyl(1→2)][β-D-xylopyranosyl(1→3)]-α-L-arabinopyranoside | B-4 | S1 | H | n | n |
| JGL-5 ^a^ | (20R,21R,23R,24R)-3β,20,21,terahydroxy-19-oxo-21,24-cyclodrammar-25-ene-3-O-[α-L-rhamnopyranosyl(1→2)][β-D-glucopyranosyl (1→3)]-α-L-arabinopyranside | A-3 | S2 | n | n | n |
| JGL-6 ^a^ | (20R,21R,23R,24R)-3β,19,20,21,23-pentahy droxy-21,24-cyclodrammar-25-ene-3-O-[α-L-rhamnopyranosyl(1→2)][β-D-xylopyranosyl (1→3)]-β-D-glucopyranside | A-2 | S4 | n | n | n |
| JGL-7 ^a^ | (20R,21R,23R,24R)-3β,19,20,21,23-pentahy droxy-21,24-cyclodrammar-25-ene-3-O-[α-L-rhamnopyranosyl(1→2)][β-D-xylopyranosyl (1→3)]-α-L-arabinopyranside | A-2 | S1 | n | n | n |
| JGL-8 ^a^ | (20S)-3β,20,21-trihydroxydammar-19-oxo-24-ene-3-O-{[α-L-rhamnopyranosyl(1→2)][β-D-xylopyranosyl(1→3)]-α-L-arabinopyranosyl}-21-O-[β-D-glucopyranosyl(1→6)]-β-D-glucopyranoside | F-1 | S1 | H | S7 | n |
| JGL-9 ^a^ | (20R)-3β,20,21-trihydroxydammar-24-ene-19-oxo-3-O-[α-L-rhamnopyranosyl(1→2)][β-D-xylopyranosyl(1→3)]-α-L-arabinopyranosyl-21-O-[α-L-rhamnopyranosyl(1→6)]-β-D-glucopyranoside | F-1 | S1 | H | S7 | n |

a: reference standards, n: no substituents

**Table S2** Grouping list of mixed reference standards.

| No. | Composition of reference standards |
| --- | --- |
| PLHB-1 | JGL-19, JGL-22, JGL-3, JGL-11, JGL-10, GL-10, GL-2, GL-21, GL-17 |
| PLHB-2 | JGL-5, JGL-7, GL-1, JGL-2, GL-20, GL-13, GL-15 |
| PLHB-3 | JGL-20, JGL-16, JGL-1, JGL-14, JGL-13, GL-8, GL-18, GL-5 |
| PLHB-4 | JGL-17, JGL-8, JGL-15, GL-7, GL-6, GL-3, GL-14 |
| PLHB-5 | JGL-18, JGL-6, JGL-4, JGL-9, GL-11, GL-4, GL-12, GL-19 |
| FJHB-1 | FJ-66, FJ-13, FJ-41, FJ-1, FJ-2, FJ-19 |
| FJHB-2 | FJ-72, FJ-8, FJ-48, FJ-27, FJ-24, FJ-23, FJ-18 |
| FJHB-3 | FJ-70, FJ-54, FJ-76, FJ-56, FJ-42, FJ-43, FJ-35, FJ-28 |
| FJHB-4 | FJ-69, FJ-64, FJ-50, FJ-4, FJ-12, FJ-37 |
| FJHB-5 | FJ-59, FJ-57, FJ-36, FJ-20, FJ-26 |
| FJHB-6 | FJ-71, FJ-74, FJ-61, FJ-5, FJ-9, FJ-49, FJ-17, FJ-25 |
| FJHB-7 | FJ-75, FJ-52, FJ-65, FJ-77, FJ-6, FJ-10, FJ-30 |
| FJHB-8 | FJ-67, FJ-58, FJ-73, FJ-7, FJ-14, FJ-31, FJ-34, FJ-22 |
| FJHB-9 | FJ-68, FJ-62, FJ-3, FJ-11, FJ-32, FJ-33 |

**Table S3** CCS prediction values of gypenosides in the Herba Gynostemma mass spectrometry database based on CCSondemand.

| No. | [M+H]^+^ | [M+Na]^+^ | [M+K]^+^ | [M–H]^−^ | [M+HCOO] ^−^ |
| --- | --- | --- | --- | --- | --- |
| 1 | 342.4 | 342.6 | 343.3 | 339.5 | 343.7 |
| 2 | 329.8 | 329.1 | 329.9 | 324.8 | 333.5 |
| 3 | 327.5 | 327.6 | 330.1 | 324.9 | 330.8 |
| 4 | 325.3 | 325.0 | 327.5 | 322.6 | 328.6 |
| 5 | 327.9 | 328.2 | 330.3 | 324.9 | 331.2 |
| 6 | 331.5 | 332.4 | 334.0 | 330.9 | 333.6 |
| 7 | 325.4 | 325.2 | 328.0 | 322.7 | 328.5 |
| 8 | 348.3 | 348.0 | 349.0 | 345.2 | 350.8 |
| 9 | 322.1 | 322.2 | 324.1 | 318.9 | 326.1 |
| 10 | 325.3 | 325.0 | 327.5 | 322.6 | 328.6 |
| 11 | 222.1 | 222.5 | 224.9 | 220.2 | 226.8 |
| 12 | 351.2 | 350.1 | 351.2 | 348.0 | 352.5 |
| 13 | 339.7 | 339.6 | 339.6 | 336.4 | 341.6 |
| 14 | 337.7 | 338.2 | 338.7 | 335.0 | 340.7 |
| 15 | 339.5 | 340.0 | 340.4 | 336.8 | 341.8 |
| 16 | 341.1 | 340.4 | 341.4 | 337.8 | 342.8 |
| 17 | 340.5 | 340.9 | 341.0 | 338.4 | 342.9 |
| 18 | 325.4 | 325.2 | 328.0 | 322.7 | 328.5 |
| 19 | 338.2 | 339.9 | 338.6 | 335.2 | 339.4 |
| 20 | 285.3 | 285.3 | 286.5 | 281.0 | 288.2 |
| 21 | 337.7 | 338.0 | 337.7 | 334.3 | 339.5 |
| 22 | 319.4 | 319.6 | 320.8 | 315.5 | 321.5 |
| 23 | 321.5 | 321.7 | 322.4 | 318.1 | 322.7 |
| 24 | 348.4 | 347.6 | 349.0 | 346.1 | 350.3 |
| 25 | 219.7 | 220.3 | 218.2 | 217.8 | 224.6 |
| 26 | 222.1 | 222.5 | 224.9 | 220.2 | 226.8 |
| 27 | 221.5 | 222.4 | 220.6 | 220.6 | 225.5 |
| 28 | 222.1 | 222.5 | 224.9 | 220.2 | 226.8 |
| 29 | 322.8 | 321.9 | 324.3 | 319.6 | 325.5 |
| 30 | 223.7 | 225.3 | 222.7 | 222.7 | 228.3 |
| 31 | 221.1 | 221.9 | 223.8 | 219.2 | 225.8 |
| 32 | 322.0 | 320.7 | 323.4 | 318.3 | 325.6 |
| 33 | 330.8 | 330.6 | 332.2 | 327.2 | 334.2 |
| 34 | 257.6 | 258.1 | 259.0 | 253.6 | 262.5 |
| 35 | 345.4 | 345.0 | 346.1 | 342.5 | 347.7 |
| 36 | 337.0 | 336.8 | 338.3 | 333.0 | 339.0 |
| 37 | 345.7 | 345.2 | 346.2 | 341.9 | 347.6 |
| 38 | 329.7 | 328.8 | 330.8 | 325.9 | 332.7 |
| 39 | 253.4 | 255.0 | 255.5 | 252.4 | 257.0 |
| 40 | 257.8 | 259.6 | 260.8 | 256.0 | 262.4 |
| 41 | 293.1 | 293.7 | 295.4 | 289.4 | 295.8 |
| 42 | 289.9 | 290.5 | 292.3 | 285.3 | 292.6 |
| 43 | 257.8 | 259.6 | 260.8 | 256.0 | 262.4 |
| 44 | 289.9 | 290.5 | 292.3 | 285.3 | 292.6 |
| 45 | 252.6 | 254.2 | 256.1 | 251.5 | 257.2 |
| 46 | 350.0 | 349.7 | 350.2 | 346.8 | 351.9 |
| 47 | 339.7 | 340.2 | 339.9 | 336.6 | 340.6 |
| 48 | 347.6 | 347.7 | 348.3 | 344.2 | 349.1 |
| 49 | 326.9 | 325.7 | 327.9 | 323.0 | 328.9 |
| 50 | 286.7 | 286.1 | 288.3 | 282.9 | 290.6 |
| 51 | 328.0 | 327.1 | 329.5 | 323.6 | 332.0 |
| 52 | 287.1 | 286.5 | 288.7 | 283.4 | 291.0 |
| 53 | 329.0 | 328.3 | 330.5 | 324.4 | 333.0 |
| 54 | 286.9 | 286.0 | 288.4 | 283.0 | 290.6 |
| 55 | 331.7 | 331.7 | 333.0 | 327.3 | 334.8 |
| 56 | 321.4 | 320.4 | 323.8 | 317.4 | 323.7 |
| 57 | 323.1 | 322.8 | 325.4 | 319.7 | 325.5 |
| 58 | 348.1 | 347.7 | 348.8 | 345.0 | 350.5 |
| 59 | 341.0 | 340.9 | 341.5 | 338.7 | 343.4 |
| 60 | 331.8 | 332.3 | 332.5 | 326.5 | 335.4 |
| 61 | 342.2 | 341.1 | 342.5 | 339.2 | 343.8 |
| 62 | 342.2 | 342.0 | 343.1 | 339.7 | 343.7 |
| 63 | 349.4 | 350.1 | 350.3 | 347.0 | 351.5 |
| 64 | 341.4 | 340.1 | 341.5 | 337.7 | 342.8 |
| 65 | 349.5 | 350.0 | 350.0 | 347.1 | 351.3 |
| 66 | 329.4 | 330.1 | 331.4 | 327.2 | 331.3 |
| 67 | 344.0 | 344.1 | 344.0 | 341.5 | 344.7 |
| 68 | 332.0 | 333.4 | 332.2 | 329.2 | 333.2 |
| 69 | 338.0 | 339.5 | 338.4 | 335.0 | 339.3 |
| 70 | 338.7 | 340.2 | 339.0 | 336.0 | 339.8 |
| 71 | 344.0 | 344.7 | 344.3 | 341.0 | 345.2 |
| 72 | 338.1 | 338.4 | 338.0 | 334.6 | 339.1 |
| 73 | 338.6 | 339.0 | 338.6 | 335.7 | 339.7 |
| 74 | 319.4 | 319.6 | 320.8 | 315.5 | 321.5 |
| 75 | 338.8 | 339.3 | 338.9 | 336.0 | 339.7 |
| 76 | 339.5 | 340.2 | 339.6 | 336.4 | 340.4 |
| 77 | 345.5 | 345.0 | 345.8 | 342.7 | 346.6 |
| 78 | 329.4 | 329.5 | 330.3 | 324.8 | 332.3 |
| 79 | 329.9 | 328.9 | 332.2 | 325.9 | 332.2 |
| 80 | 343.9 | 344.5 | 344.2 | 341.0 | 345.2 |
| 81 | 345.4 | 345.6 | 345.7 | 342.8 | 346.7 |
| 82 | 345.2 | 344.5 | 345.5 | 342.1 | 347.3 |
| 83 | 342.2 | 341.0 | 342.7 | 339.9 | 343.7 |
| 84 | 346.1 | 345.5 | 346.5 | 343.2 | 348.5 |
| 85 | 346.5 | 346.4 | 347.2 | 343.0 | 348.2 |
| 86 | 332.9 | 333.0 | 333.5 | 329.1 | 334.3 |
| 87 | 339.7 | 339.4 | 340.0 | 335.7 | 342.0 |
| 88 | 329.6 | 329.2 | 331.8 | 326.9 | 333.3 |
| 89 | 349.9 | 349.9 | 350.8 | 347.4 | 352.3 |
| 90 | 337.3 | 337.7 | 338.3 | 334.5 | 340.3 |
| 91 | 328.3 | 328.7 | 330.9 | 324.9 | 332.9 |
| 92 | 339.4 | 340.0 | 340.5 | 336.6 | 342.4 |
| 93 | 259.5 | 260.9 | 261.7 | 256.9 | 263.5 |
| 94 | 292.6 | 292.5 | 294.1 | 287.4 | 296.6 |
| 95 | 260.4 | 260.3 | 262.6 | 257.8 | 264.5 |
| 96 | 218.3 | 218.6 | 216.8 | 216.5 | 222.3 |
| 97 | 217.6 | 219.3 | 216.5 | 216.0 | 221.6 |
| 98 | 292.6 | 292.5 | 294.1 | 287.4 | 296.6 |
| 99 | 260.4 | 260.3 | 262.6 | 257.8 | 264.5 |
| 100 | 223.8 | 226.1 | 222.4 | 221.4 | 227.7 |
| 101 | 319.0 | 319.7 | 321.4 | 315.8 | 324.3 |
| 102 | 255.6 | 256.5 | 257.8 | 252.4 | 260.1 |
| 103 | 221.1 | 221.6 | 219.8 | 218.2 | 225.0 |
| 104 | 331.3 | 331.6 | 333.7 | 328.3 | 334.8 |
| 105 | 228.6 | 230.2 | 227.5 | 226.8 | 232.8 |
| 106 | 332.6 | 332.9 | 334.9 | 329.1 | 336.2 |
| 107 | 224.6 | 225.0 | 223.5 | 223.3 | 228.6 |
| 108 | 349.3 | 348.2 | 349.5 | 346.0 | 350.6 |
| 109 | 350.3 | 349.7 | 351.0 | 347.0 | 351.7 |
| 110 | 325.1 | 324.0 | 326.5 | 320.9 | 327.5 |
| 111 | 325.1 | 324.0 | 326.5 | 320.9 | 327.5 |
| 112 | 311.9 | 310.9 | 313.1 | 307.8 | 313.9 |
| 113 | 327.2 | 327.0 | 328.9 | 323.3 | 331.2 |
| 114 | 329.8 | 329.1 | 329.9 | 324.8 | 333.5 |
| 115 | 298.2 | 298.1 | 300.4 | 295.0 | 301.6 |
| 116 | 312.1 | 312.1 | 314.4 | 308.8 | 315.7 |
| 117 | 329.7 | 329.9 | 331.8 | 326.6 | 334.1 |
| 118 | 327.2 | 327.0 | 328.9 | 323.3 | 331.2 |
| 119 | 330.9 | 330.0 | 331.3 | 326.1 | 334.2 |
| 120 | 331.5 | 331.2 | 332.8 | 327.0 | 333.7 |
| 121 | 333.5 | 332.5 | 333.6 | 328.8 | 336.7 |
| 122 | 329.8 | 329.1 | 329.9 | 324.8 | 333.5 |
| 123 | 330.8 | 330.6 | 332.2 | 327.2 | 334.2 |
| 124 | 333.5 | 332.5 | 333.6 | 328.8 | 336.7 |
| 125 | 330.8 | 330.6 | 332.2 | 327.2 | 334.2 |
| 126 | 330.1 | 329.4 | 330.2 | 325.0 | 333.4 |
| 127 | 331.2 | 331.7 | 332.7 | 327.7 | 334.4 |
| 128 | 260.9 | 262.7 | 263.2 | 259.2 | 265.5 |
| 129 | 217.9 | 219.2 | 214.1 | 216.5 | 223.6 |
| 130 | 218.3 | 218.6 | 216.8 | 216.5 | 222.3 |
| 131 | 305.4 | 304.5 | 307.1 | 301.5 | 308.6 |
| 132 | 305.2 | 304.2 | 306.7 | 300.7 | 308.4 |
| 133 | 257.5 | 257.9 | 259.1 | 255.3 | 261.6 |
| 134 | 258.4 | 258.6 | 259.8 | 256.3 | 262.3 |
| 135 | 260.2 | 259.9 | 262.3 | 257.1 | 263.8 |
| 136 | 349.9 | 348.8 | 350.1 | 346.9 | 352.2 |
| 137 | 282.6 | 281.4 | 284.2 | 278.9 | 286.4 |
| 138 | 293.8 | 293.6 | 295.3 | 289.0 | 297.3 |
| 139 | 322.7 | 323.8 | 324.3 | 319.2 | 325.4 |
| 140 | 261.1 | 261.7 | 263.4 | 257.7 | 265.9 |
| 141 | 323.5 | 324.4 | 324.2 | 319.9 | 325.3 |
| 142 | 284.1 | 284.4 | 285.4 | 279.4 | 288.2 |
| 143 | 283.5 | 283.3 | 284.7 | 279.1 | 287.9 |
| 144 | 294.2 | 293.9 | 296.2 | 290.5 | 297.7 |
| 145 | 291.5 | 290.5 | 292.9 | 286.7 | 295.1 |
| 146 | 330.8 | 329.7 | 331.7 | 326.4 | 332.9 |
| 147 | 349.8 | 349.3 | 350.0 | 347.1 | 350.8 |
| 148 | 294.6 | 293.6 | 295.7 | 290.0 | 297.5 |
| 149 | 349.5 | 348.8 | 349.8 | 346.6 | 351.4 |
| 150 | 346.6 | 346.5 | 346.9 | 343.1 | 348.3 |
| 151 | 285.4 | 286.0 | 286.6 | 281.5 | 289.2 |
| 152 | 308.4 | 308.0 | 309.8 | 305.7 | 311.6 |
| 153 | 285.4 | 286.0 | 286.6 | 281.5 | 289.2 |
| 154 | 286.8 | 285.9 | 288.3 | 282.8 | 290.5 |
| 155 | 280.6 | 281.2 | 283.1 | 275.6 | 283.4 |
| 156 | 280.6 | 281.2 | 283.1 | 275.6 | 283.4 |
| 157 | 294.0 | 293.8 | 295.7 | 289.3 | 297.9 |
| 158 | 291.7 | 292.3 | 293.0 | 287.2 | 295.9 |
| 159 | 284.7 | 284.6 | 286.9 | 279.7 | 288.5 |
| 160 | 339.1 | 339.5 | 340.0 | 336.3 | 341.4 |
| 161 | 328.0 | 326.9 | 329.3 | 324.3 | 330.5 |
| 162 | 346.3 | 346.4 | 346.7 | 342.6 | 347.8 |
| 163 | 347.1 | 347.1 | 348.3 | 344.2 | 349.2 |
| 164 | 340.8 | 341.3 | 342.3 | 338.6 | 342.9 |
| 165 | 346.4 | 346.5 | 347.8 | 344.2 | 348.6 |
| 166 | 349.3 | 348.7 | 350.2 | 346.5 | 351.8 |
| 167 | 345.7 | 346.7 | 347.2 | 344.2 | 348.3 |
| 168 | 344.7 | 346.0 | 346.2 | 343.1 | 347.2 |
| 169 | 283.1 | 282.7 | 285.2 | 278.3 | 286.8 |
| 170 | 291.5 | 290.5 | 292.9 | 285.8 | 294.1 |
| 171 | 291.8 | 291.1 | 293.1 | 287.0 | 294.6 |
| 172 | 295.2 | 294.3 | 296.4 | 290.0 | 298.2 |
| 173 | 295.1 | 294.6 | 297.0 | 291.0 | 298.9 |
| 174 | 293.9 | 293.7 | 296.2 | 290.3 | 298.1 |
| 175 | 328.9 | 329.0 | 330.7 | 324.8 | 331.7 |
| 176 | 330.1 | 330.2 | 332.3 | 326.1 | 333.3 |
| 177 | 347.6 | 346.9 | 348.2 | 343.3 | 348.9 |
| 178 | 295.1 | 294.6 | 297.0 | 291.0 | 298.9 |
| 179 | 347.0 | 346.8 | 347.9 | 344.2 | 348.5 |
| 180 | 348.0 | 347.3 | 348.3 | 344.6 | 349.7 |
| 181 | 331.6 | 330.9 | 333.0 | 326.2 | 334.7 |
| 182 | 348.8 | 347.9 | 349.0 | 346.3 | 350.3 |
| 183 | 348.0 | 347.3 | 348.2 | 344.7 | 349.6 |
| 184 | 348.1 | 346.9 | 348.3 | 345.2 | 350.1 |
| 185 | 349.9 | 348.8 | 350.1 | 346.9 | 352.2 |
| 186 | 261.2 | 261.1 | 263.4 | 258.1 | 265.1 |
| 187 | 295.9 | 295.3 | 297.7 | 291.5 | 299.7 |
| 188 | 326.8 | 328.3 | 329.1 | 323.4 | 330.4 |
| 189 | 286.8 | 286.4 | 288.7 | 282.0 | 290.5 |
| 190 | 339.5 | 339.1 | 339.7 | 335.8 | 342.0 |
| 191 | 288.3 | 287.0 | 289.4 | 283.6 | 291.6 |
| 192 | 330.0 | 329.4 | 331.1 | 325.6 | 332.6 |
| 193 | 340.2 | 339.1 | 340.5 | 337.7 | 342.4 |
| 194 | 333.1 | 333.5 | 333.8 | 330.1 | 335.3 |
| 195 | 341.3 | 341.0 | 341.5 | 338.2 | 344.0 |
| 196 | 330.1 | 330.2 | 331.6 | 326.1 | 333.4 |
| 197 | 346.0 | 345.7 | 346.5 | 342.4 | 348.1 |
| 198 | 283.5 | 283.0 | 285.0 | 278.5 | 287.2 |
| 199 | 292.5 | 291.9 | 293.9 | 287.5 | 295.8 |
| 200 | 340.6 | 340.2 | 341.0 | 337.7 | 343.0 |
| 201 | 340.6 | 339.6 | 340.8 | 337.1 | 343.3 |
| 202 | 346.5 | 346.1 | 346.9 | 342.8 | 348.7 |
| 203 | 330.7 | 331.0 | 332.2 | 327.2 | 333.5 |
| 204 | 331.4 | 330.9 | 332.7 | 327.1 | 334.6 |
| 205 | 296.3 | 295.2 | 297.7 | 292.2 | 299.3 |
| 206 | 294.4 | 294.0 | 296.2 | 289.5 | 298.2 |
| 207 | 291.2 | 290.4 | 292.8 | 286.9 | 295.0 |
| 208 | 260.2 | 260.9 | 262.7 | 257.0 | 264.6 |
| 209 | 283.4 | 282.4 | 284.9 | 279.8 | 287.3 |
| 210 | 347.3 | 346.3 | 347.6 | 344.3 | 348.7 |
| 211 | 340.9 | 340.0 | 341.2 | 338.5 | 343.1 |
| 212 | 332.0 | 332.1 | 332.6 | 328.4 | 333.9 |
| 213 | 332.9 | 333.0 | 333.6 | 329.8 | 335.1 |
| 214 | 338.9 | 339.9 | 339.9 | 336.5 | 341.9 |
| 215 | 337.6 | 338.3 | 338.9 | 335.2 | 341.1 |
| 216 | 349.2 | 348.4 | 349.5 | 346.4 | 351.2 |
| 217 | 348.8 | 347.9 | 349.1 | 345.5 | 350.8 |
| 218 | 340.2 | 339.3 | 340.4 | 337.1 | 342.1 |
| 219 | 341.0 | 340.3 | 341.3 | 338.1 | 342.7 |
| 220 | 341.9 | 342.6 | 342.2 | 339.6 | 344.0 |
| 221 | 333.2 | 332.9 | 333.5 | 328.9 | 335.4 |
| 222 | 333.2 | 332.9 | 333.5 | 328.9 | 335.4 |
| 223 | 338.9 | 339.1 | 339.5 | 335.2 | 340.5 |
| 224 | 329.5 | 329.3 | 331.2 | 326.1 | 333.3 |
| 225 | 320.1 | 320.5 | 322.1 | 315.6 | 324.2 |
| 226 | 329.1 | 329.1 | 330.2 | 325.0 | 333.7 |
| 227 | 330.3 | 330.0 | 331.8 | 326.8 | 333.3 |
| 228 | 346.7 | 346.5 | 347.2 | 343.3 | 348.3 |
| 229 | 329.0 | 328.1 | 330.5 | 325.5 | 332.0 |
| 230 | 348.7 | 348.8 | 348.9 | 345.4 | 350.3 |
| 231 | 337.8 | 337.8 | 338.5 | 334.0 | 338.9 |
| 232 | 348.1 | 348.1 | 348.9 | 345.1 | 349.7 |
| 233 | 328.0 | 326.9 | 329.3 | 324.3 | 330.5 |
| 234 | 337.8 | 337.8 | 338.5 | 333.9 | 338.8 |
| 235 | 349.7 | 349.0 | 350.3 | 346.9 | 351.5 |
| 236 | 282.8 | 281.8 | 283.9 | 278.9 | 285.9 |
| 237 | 348.0 | 347.2 | 348.2 | 344.6 | 349.5 |
| 238 | 330.5 | 330.0 | 332.0 | 327.0 | 333.4 |
| 239 | 282.6 | 281.4 | 284.2 | 278.9 | 286.4 |
| 240 | 291.1 | 290.4 | 292.8 | 286.7 | 295.0 |
| 241 | 348.9 | 347.9 | 349.1 | 345.4 | 351.0 |
| 242 | 293.2 | 292.5 | 295.1 | 288.5 | 296.6 |
| 243 | 292.5 | 293.4 | 293.8 | 287.8 | 296.6 |
| 244 | 345.7 | 345.4 | 346.4 | 343.0 | 347.3 |
| 245 | 331.2 | 330.9 | 333.1 | 326.4 | 334.5 |
| 246 | 329.6 | 329.3 | 331.4 | 324.4 | 332.9 |
| 247 | 345.8 | 345.8 | 346.6 | 342.9 | 347.3 |
| 248 | 342.0 | 341.9 | 342.9 | 338.9 | 345.2 |
| 249 | 340.2 | 339.1 | 340.5 | 337.7 | 342.4 |
| 250 | 340.8 | 339.9 | 341.1 | 338.4 | 343.1 |
| 251 | 329.7 | 329.3 | 331.2 | 326.2 | 332.4 |
| 252 | 347.2 | 347.0 | 347.6 | 343.9 | 348.9 |
| 253 | 334.7 | 334.5 | 335.1 | 331.5 | 336.3 |
| 254 | 283.4 | 283.2 | 285.3 | 279.0 | 287.8 |
| 255 | 338.2 | 338.2 | 338.1 | 334.6 | 340.9 |
| 256 | 329.1 | 329.8 | 330.5 | 325.3 | 332.4 |
| 257 | 259.8 | 261.6 | 261.6 | 256.6 | 264.3 |
| 258 | 328.6 | 329.4 | 330.7 | 324.9 | 332.5 |
| 259 | 264.3 | 264.9 | 266.8 | 260.7 | 268.0 |
| 260 | 283.1 | 283.4 | 284.8 | 278.3 | 287.1 |
| 261 | 325.4 | 326.1 | 327.5 | 321.6 | 329.4 |
| 262 | 256.9 | 257.2 | 258.9 | 253.9 | 260.6 |
| 263 | 326.2 | 327.0 | 328.4 | 322.6 | 330.5 |
| 264 | 292.5 | 293.4 | 293.8 | 287.8 | 296.6 |
| 265 | 258.7 | 260.7 | 260.6 | 256.9 | 262.8 |
| 266 | 294.3 | 294.5 | 295.9 | 288.6 | 298.2 |
| 267 | 291.7 | 291.6 | 293.7 | 287.0 | 295.7 |
| 268 | 293.1 | 292.9 | 295.1 | 288.4 | 297.1 |
| 269 | 293.1 | 292.8 | 295.4 | 288.7 | 297.3 |
| 270 | 336.6 | 337.0 | 337.7 | 333.8 | 339.0 |
| 271 | 296.2 | 295.7 | 297.9 | 291.0 | 299.8 |
| 272 | 330.6 | 331.0 | 331.7 | 327.9 | 333.4 |
| 273 | 341.5 | 341.4 | 342.4 | 339.0 | 344.3 |
| 274 | 340.2 | 340.1 | 341.1 | 337.5 | 343.1 |
| 275 | 296.2 | 295.7 | 297.9 | 291.0 | 299.8 |
| 276 | 216.4 | 220.0 | 217.7 | 214.6 | 221.6 |
| 277 | 216.0 | 219.4 | 213.2 | 214.0 | 221.3 |
| 278 | 223.2 | 225.2 | 224.0 | 220.2 | 229.5 |
| 279 | 221.1 | 221.9 | 223.8 | 219.2 | 225.8 |
| 280 | 219.5 | 220.8 | 222.3 | 218.7 | 224.5 |
| 281 | 219.5 | 220.8 | 222.3 | 218.7 | 224.5 |
| 282 | 235.5 | 237.7 | 238.5 | 236.9 | 240.8 |
| 283 | 260.5 | 262.5 | 261.9 | 258.4 | 266.1 |
| 284 | 260.8 | 261.1 | 262.2 | 257.4 | 264.7 |
| 285 | 255.2 | 255.6 | 256.7 | 252.3 | 258.5 |
| 286 | 255.6 | 255.8 | 257.2 | 253.6 | 260.4 |
| 287 | 215.4 | 216.2 | 214.3 | 213.2 | 218.9 |
| 288 | 265.1 | 267.7 | 267.8 | 264.2 | 271.1 |
| 289 | 261.0 | 262.8 | 264.1 | 259.3 | 264.5 |
| 290 | 260.0 | 261.8 | 262.5 | 258.3 | 265.4 |
| 291 | 321.1 | 320.5 | 322.9 | 317.8 | 325.0 |
| 292 | 345.8 | 346.6 | 346.9 | 344.2 | 347.3 |
| 293 | 329.2 | 329.2 | 331.2 | 325.7 | 332.9 |
| 294 | 341.2 | 341.1 | 341.6 | 336.8 | 342.6 |
| 295 | 290.4 | 289.4 | 292.0 | 285.0 | 294.1 |
| 296 | 329.7 | 329.2 | 330.4 | 324.9 | 333.4 |
| 297 | 346.1 | 346.4 | 346.7 | 342.9 | 347.9 |
| 298 | 221.5 | 224.1 | 220.9 | 220.5 | 225.2 |
| 299 | 296.7 | 295.2 | 298.2 | 292.0 | 299.3 |
| 300 | 297.0 | 295.0 | 298.5 | 292.0 | 299.5 |
| 301 | 293.4 | 292.4 | 294.8 | 289.4 | 296.6 |
| 302 | 347.0 | 346.2 | 347.2 | 343.5 | 349.0 |
| 303 | 348.0 | 347.6 | 348.5 | 344.8 | 349.7 |
| 304 | 348.5 | 347.6 | 348.9 | 344.2 | 349.3 |
| 305 | 350.8 | 350.9 | 350.9 | 348.3 | 351.6 |
| 306 | 347.2 | 346.9 | 347.5 | 343.9 | 348.9 |
| 307 | 330.0 | 329.8 | 331.5 | 326.4 | 333.0 |
| 308 | 346.1 | 346.0 | 346.6 | 342.9 | 347.0 |
| 309 | 330.9 | 330.7 | 332.9 | 327.3 | 334.4 |
| 310 | 328.4 | 328.3 | 329.7 | 323.4 | 331.8 |
| 311 | 331.5 | 331.5 | 333.6 | 328.2 | 335.0 |
| 312 | 326.6 | 327.5 | 328.9 | 323.9 | 331.6 |
| 313 | 329.7 | 329.5 | 331.4 | 326.6 | 332.8 |
| 314 | 331.5 | 332.4 | 334.0 | 330.9 | 333.6 |
| 315 | 337.5 | 339.0 | 337.8 | 334.5 | 338.8 |
| 316 | 338.8 | 339.2 | 338.8 | 336.3 | 339.8 |
| 317 | 228.9 | 230.4 | 228.3 | 228.0 | 233.6 |
| 318 | 261.0 | 261.6 | 263.2 | 258.0 | 264.5 |
| 319 | 324.8 | 325.2 | 326.3 | 320.5 | 328.1 |
| 320 | 339.2 | 339.5 | 339.6 | 336.2 | 340.7 |
| 321 | 264.7 | 265.1 | 267.5 | 261.3 | 269.5 |
| 322 | 260.0 | 260.9 | 261.3 | 257.2 | 263.7 |
| 323 | 229.7 | 231.3 | 232.3 | 228.9 | 234.4 |
| 324 | 229.7 | 231.3 | 232.3 | 228.9 | 234.4 |
| 325 | 229.5 | 232.2 | 232.0 | 228.9 | 233.5 |
| 326 | 258.6 | 259.8 | 260.7 | 257.0 | 262.8 |
| 327 | 345.4 | 345.3 | 346.1 | 342.5 | 347.1 |
| 328 | 326.0 | 325.1 | 327.4 | 321.4 | 329.2 |
| 329 | 328.7 | 327.1 | 329.4 | 324.4 | 330.7 |
| 330 | 329.6 | 328.4 | 330.8 | 325.9 | 332.0 |
| 331 | 218.5 | 219.8 | 218.2 | 216.1 | 223.8 |
| 332 | 255.3 | 256.6 | 257.1 | 251.6 | 260.1 |
| FJ-1 | 293.1 | 292.4 | 294.8 | 288.6 | 296.8 |
| FJ-10 | 289.7 | 288.8 | 290.9 | 284.5 | 292.9 |
| FJ-11 | 290.0 | 289.2 | 291.7 | 285.4 | 293.7 |
| FJ-12 | 283.9 | 283.2 | 285.1 | 279.9 | 287.2 |
| FJ-13 | 331.5 | 331.2 | 333.3 | 327.9 | 334.7 |
| FJ-14 | 281.9 | 281.2 | 283.6 | 278.3 | 285.6 |
| FJ-16 | 261.0 | 260.1 | 263.1 | 258.1 | 264.2 |
| FJ-17 | 260.8 | 259.9 | 262.9 | 257.5 | 264.2 |
| FJ-18 | 261.2 | 261.1 | 263.4 | 258.1 | 265.1 |
| FJ-19 | 261.3 | 261.2 | 263.5 | 258.4 | 264.8 |
| FJ-2 | 282.8 | 281.8 | 283.9 | 278.9 | 285.9 |
| FJ-20 | 292.6 | 291.5 | 294.0 | 288.5 | 295.3 |
| FJ-22 | 292.0 | 291.1 | 292.3 | 288.0 | 295.1 |
| FJ-23 | 282.6 | 281.4 | 284.2 | 278.9 | 286.4 |
| FJ-24 | 292.5 | 291.6 | 294.0 | 288.0 | 296.0 |
| FJ-25 | 261.3 | 261.0 | 263.9 | 259.1 | 264.9 |
| FJ-26 | 291.7 | 290.7 | 292.3 | 287.9 | 294.8 |
| FJ-27 | 328.5 | 327.8 | 330.0 | 325.0 | 331.2 |
| FJ-28 | 295.9 | 295.3 | 297.7 | 291.5 | 299.7 |
| FJ-29 | 260.6 | 260.4 | 262.7 | 257.6 | 264.5 |
| FJ-3 | 295.8 | 295.2 | 298.7 | 291.1 | 300.6 |
| FJ-30 | 260.8 | 259.9 | 262.9 | 257.5 | 264.2 |
| FJ-31 | 260.8 | 260.7 | 263.5 | 258.4 | 265.2 |
| FJ-32 | 261.4 | 260.7 | 264.2 | 258.7 | 265.5 |
| FJ-33 | 287.3 | 285.6 | 289.3 | 283.9 | 291.1 |
| FJ-34 | 282.0 | 281.5 | 284.0 | 278.0 | 285.0 |
| FJ-35 | 261.4 | 260.9 | 263.3 | 257.5 | 265.7 |
| FJ-36 | 260.6 | 260.4 | 262.7 | 257.6 | 264.5 |
| FJ-37 | 282.5 | 281.8 | 283.9 | 278.6 | 286.4 |
| FJ-4 | 294.3 | 294.1 | 296.6 | 290.3 | 298.4 |
| FJ-41 | 328.7 | 328.3 | 330.6 | 324.2 | 332.1 |
| FJ-42 | 329.7 | 330.1 | 331.7 | 325.8 | 333.4 |
| FJ-43 | 340.7 | 340.8 | 340.5 | 337.2 | 343.2 |
| FJ-48 | 329.9 | 329.5 | 331.4 | 326.4 | 332.9 |
| FJ-49 | 320.4 | 320.0 | 322.3 | 316.1 | 323.9 |
| FJ-5 | 293.3 | 293.1 | 295.8 | 288.2 | 297.5 |
| FJ-50 | 326.1 | 326.2 | 328.0 | 321.5 | 329.0 |
| FJ-52 | 322.8 | 322.0 | 324.3 | 318.0 | 326.2 |
| FJ-53 | 329.6 | 329.5 | 331.7 | 325.7 | 332.6 |
| FJ-56 | 349.0 | 348.1 | 349.7 | 345.7 | 351.5 |
| FJ-57 | 326.1 | 326.2 | 328.0 | 321.5 | 329.0 |
| FJ-58 | 332.2 | 332.4 | 333.4 | 328.6 | 334.1 |
| FJ-59 | 331.4 | 331.4 | 332.4 | 327.5 | 333.0 |
| FJ-6 | 293.3 | 293.1 | 295.8 | 288.2 | 297.5 |
| FJ-61 | 331.2 | 330.9 | 332.1 | 327.0 | 332.5 |
| FJ-62 | 331.8 | 331.8 | 333.0 | 327.9 | 333.5 |
| FJ-63 | 339.0 | 338.8 | 339.6 | 335.2 | 340.7 |
| FJ-64 | 331.4 | 331.4 | 332.4 | 327.5 | 333.0 |
| FJ-65 | 331.2 | 330.9 | 332.1 | 327.0 | 332.5 |
| FJ-66 | 345.4 | 345.3 | 346.1 | 342.5 | 347.1 |
| FJ-67 | 345.4 | 345.6 | 346.5 | 342.9 | 347.2 |
| FJ-68 | 345.7 | 345.6 | 346.7 | 342.9 | 347.3 |
| FJ-69 | 346.0 | 345.6 | 346.7 | 343.1 | 347.7 |
| FJ-7 | 294.6 | 294.8 | 297.6 | 291.4 | 299.4 |
| FJ-70 | 338.2 | 338.2 | 339.0 | 335.4 | 340.5 |
| FJ-71 | 346.6 | 346.2 | 347.3 | 343.5 | 348.2 |
| FJ-72 | 346.0 | 345.6 | 346.7 | 343.1 | 347.7 |
| FJ-73 | 328.3 | 328.4 | 330.5 | 324.0 | 331.6 |
| FJ-74 | 340.5 | 339.6 | 341.2 | 336.3 | 342.1 |
| FJ-75 | 346.0 | 345.5 | 346.7 | 342.3 | 347.8 |
| FJ-76 | 345.3 | 345.0 | 346.0 | 342.4 | 346.9 |
| FJ-77 | 346.6 | 346.2 | 347.3 | 343.5 | 348.2 |
| FJ-8 | 294.3 | 294.1 | 296.6 | 290.3 | 298.4 |
| FJ-9 | 290.9 | 290.6 | 293.3 | 286.0 | 294.7 |
| GL-1 | 329.1 | 329.0 | 330.9 | 326.0 | 333.2 |
| GL-10 | 339.8 | 339.9 | 340.0 | 336.6 | 342.7 |
| GL-11 | 341.2 | 341.2 | 341.4 | 338.1 | 343.9 |
| GL-12 | 326.4 | 326.6 | 328.7 | 323.2 | 329.6 |
| GL-13 | 326.4 | 326.6 | 328.7 | 323.2 | 329.6 |
| GL-14 | 329.4 | 330.1 | 331.4 | 327.2 | 331.3 |
| GL-15 | 329.4 | 330.1 | 331.4 | 327.2 | 331.3 |
| GL-17 | 330.7 | 331.2 | 333.1 | 327.8 | 333.0 |
| GL-18 | 328.3 | 328.3 | 328.9 | 323.3 | 331.5 |
| GL-19 | 329.5 | 328.9 | 330.9 | 325.8 | 333.1 |
| GL-2 | 320.5 | 320.6 | 322.7 | 317.3 | 324.5 |
| GL-20 | 339.5 | 339.4 | 340.4 | 336.7 | 342.7 |
| GL-21 | 327.2 | 327.0 | 328.9 | 323.3 | 331.2 |
| GL-3 | 327.9 | 328.2 | 330.3 | 324.9 | 331.2 |
| GL-4 | 327.9 | 328.2 | 330.3 | 324.9 | 331.2 |
| GL-5 | 328.7 | 328.3 | 330.6 | 325.1 | 332.8 |
| GL-6 | 324.1 | 324.1 | 326.3 | 320.8 | 328.1 |
| GL-7 | 341.9 | 342.5 | 342.3 | 339.6 | 344.2 |
| GL-8 | 325.3 | 325.0 | 327.5 | 322.6 | 328.6 |
| JGL-1 | 319.0 | 319.7 | 321.4 | 315.8 | 324.3 |
| JGL-10 | 347.2 | 346.8 | 347.8 | 344.0 | 349.1 |
| JGL-11 | 339.7 | 339.6 | 339.6 | 336.4 | 341.6 |
| JGL-13 | 341.1 | 340.4 | 341.4 | 337.8 | 342.8 |
| JGL-14 | 351.2 | 350.1 | 351.2 | 348.0 | 352.5 |
| JGL-15 | 349.9 | 349.0 | 350.5 | 347.7 | 351.6 |
| JGL-16 | 339.9 | 339.8 | 340.9 | 337.1 | 342.3 |
| JGL-17 | 319.0 | 319.7 | 321.4 | 315.8 | 324.3 |
| JGL-18 | 324.3 | 324.8 | 326.5 | 322.1 | 328.6 |
| JGL-19 | 326.0 | 326.7 | 328.2 | 323.1 | 330.9 |
| JGL-2 | 351.2 | 350.1 | 351.2 | 348.0 | 352.5 |
| JGL-20 | 326.0 | 326.7 | 328.2 | 323.1 | 330.9 |
| JGL-3 | 339.9 | 339.8 | 340.9 | 337.1 | 342.3 |
| JGL-4 | 326.1 | 326.7 | 328.1 | 322.0 | 331.2 |
| JGL-5 | 320.6 | 321.3 | 322.7 | 316.7 | 325.4 |
| JGL-6 | 322.1 | 322.2 | 324.1 | 318.9 | 326.1 |
| JGL-7 | 319.4 | 319.3 | 321.4 | 315.5 | 324.2 |
| JGL-8 | 339.5 | 340.0 | 340.4 | 336.8 | 341.8 |
| JGL-9 | 337.0 | 337.0 | 337.7 | 333.6 | 338.6 |

**Table S4** Identification of chemical components from the three parts of *G. longipes.*

| No. | *Rt*   (min) | Observed  *m/z* | Adduct | Formula | Mass error  (mDa) | Mass error  (ppm) | CCS   (*m/z*) | Fragments | Part | | | Component name |
| --- | --- | --- | --- | --- | --- | --- | --- | --- | --- | --- | --- | --- |
|  |  |  |  |  |  |  |  |  | G | J | Y |  |
| 1 | 1.78 | 771.1957 | -H | C_33_H_40_O_21_ | -2.7 | -3.5 | 258.36  (771) | neg: 771.1957, 609.1456, 301.0337 |  | + |  | 4G-α-D-glucopyranosylrutin |
| 2 | 2.24 | 609.1438 | -H | C_27_H_30_O_16_ | -1.8 | -3 | 232.02  (609) | neg: 609.1438, 301.0330, 271.0236, 255.0288 | + | + | + | rutin |
| 3 | 2.39 | 463.0870 | -H | C_21_H_20_O_12_ | -0.7 | -1.5 | 200.35  (463) | neg: 463.0870, 300.0302, 271.0232, 255.0300, 241.0126 |  | + | + | quercetin-7-O-β-D-glucoside |
| 4 | 2.42 | 593.1490 | -H | C_27_H_30_O_15_ | -1.6 | -2.7 | 230.19  (593) | neg: 593.1490, 284.0314, 255.0287, 227.0340 |  | + | + | kaempferol-3-O-α-L-rhamnopyranosyl-(1→2)-β-D-glucopyranoside |
| 5 | 2.56 | 593.1496 | -H | C_27_H_30_O_15_ | -1 | -1.7 | 228.72  (593) | neg: 593.1496, 285.0389, 255.0288, 227.0340 | + | + | + | nicotiflorin |
| 6 | 2.58 | 623.1604 | -H | C_28_H_32_O_16_ | -0.8 | -1.3 | 235.73  (623) | neg: 623.1604, 315.0497, 299.0196, 271.0237 |  | + | + | Isorhamnetin 3-rutinoside |
| 7 | 2.71 | 447.0928 | -H | C_21_H_20_O_11_ | 0.1 | 0.2 | 198.82  (447) | neg: 447.0928, 285.0382, 255.0290, 227.0338 |  | + | + | kaempferol-7-O-β-D-galactopyranoside |
| 8 | 2.77 | 477.1031 | -H | C_21_H_22_O_12_ | -0.2 | -0.4 | 206.92  (477) | neg: 477.1031, 314.0431, 271.0231, 243.0285, 227.0337 |  | + | + | isorhamnetin-7-O-β-D-glucopyranoside |
| 9 | 2.81 | 363.0712 | -H | C_17_H_16_O_9_ | -0.4 | -1.1 | 248.89  (363) | neg: 363.0712, 345.0607, 300.0264, 271.0236 | + | + | + | 2H-1-Benzopyran-6-carboxylic acid, 3,4-dihydro-3,5,7-trihydroxy-2-(3,4,5-trihydroxyphenyl)-, methyl ester, (2R,3R)- (ACI) |
| 10 ^n^ | 2.93 | 947.5196 | -H | C_47_H_80_O_19_ | -2 | -2.1 | 314.06  (993) | neg: 993.5210, 947.5196, 815.4786, 669.4205, 507.3716 | + |  |  | E-3+OH+Glc+Rha+Xyl |
| 11 | 3.03 | 915.4976 | -H | C_46_H_76_O_18_ | 2.3 | 2.5 | 329.22  (961) | neg: 961.4943, 915.4976, 783.4515, 637.3942, 505.3528, 411.2887  pos: 471.3476, 453.3369, 435.327 | + |  | + | isomer of 18-Norandrostan-19-al, 3-[O-6-α-L-rhamnopyranosyl-(1→2)-O-[β-D-xylo- pyranosyl-(1→3)]-α-L-arabinopyranosyl-oxy]-4,4,8,14-tetramethyl-17-[(1S)-1,2,4-trihydroxy-3-(1-hydroxy-1-methylethyl) cyclopentyl]-, (3β,5α,17β)- (9CI, ACI) |
| 12 | 3.03 | 917.5148 | -H | C_46_H_78_O_18_ | 3.8 | 4.1 | 332.28  (963) | neg: 963.5106, 917.5148, 785.4668, 639.4117, 545.3478 | + |  | + | β-D-Glucopyranoside, (3β,12β,23S,24R)-20,25-epoxy-12,23,24-trihydroxydamma-ran-3-yl O-β-D-xylopyranosyl-(1→2)-O-[β-D-xylopyranosyl-(1→6)]- (9CI) |
| 13 ^n^ | 3.06 | 1031.4994 | -H | C_50_H_80_O_22_ | -4.8 | -4.7 | 335.52  (1031) | neg: 1031.4994, 987.5038, 945.5002, 783.4523, 637.3951, 505.3536 |  |  | + | E-1+Glc+Rha+Xyl+Mal |
| 14 ^n^ | 3.26 | 917.5129 | -H | C_46_H_78_O_18_ | 1.9 | 2.1 | 315.75  (963) | neg: 963.5112, 917.5129, 785.4664, 639.4102, 507.3680 | + | + | + | Q-1+OH+Ara+Rha+Xyl |
| 15 ^n^ | 3.40 | 1001.4901 | -H | C_49_H_78_O_21_ | -5.6 | -5.6 | 316.41  (1001) | neg: 1001.4901, 915.4879, 783.4509, 637.3928, 505.3528  pos: 471.3468, 453.3360, 435.3264 | + | + | + | E-1+Ara+Rha+Xyl+Mal |
| 16 ^n^ | 3.42 | 1045.5227 | -H | C_51_H_82_O_22_ | 0.8 | 0.8 | 337.1  (1091) | neg: 1091.5204, 1045.5227, 961.4634, 829.4199, 667.3721, 521.3118, 389.2681  pos: 1064.5646, 457.3327, 439.3215, 421.3129 | + | + | + | C-1+OH+Ara+Rha+Xyl+Glc |
| 17 ^n^ | 3.48 | 945.5043 | -H | C_47_H_78_O_19_ | -1.6 | -1.7 | 338.11  (991) | neg: 991.5052, 945.5043, 783.4519, 637.3961, 505.3530  pos: 489.3559, 471.3486, 453.3368, 435.3268 |  |  | + | E-1+Ara+Rha+Glc |
| 18 | 3.50 | 1061.5494 | -H | C_52_H_86_O_22_ | -3.8 | -3.6 | 335.77  (1107) | neg: 1107.5519, 1061.5494, 929.5072, 767.4583, 621.3981, 521.3060, 389.2625 | + |  |  | Dammar-25-en-19-al, 3β-[O-α-L-rhamno-pyranosyl-(1→2)-O-[β-D-xylopyranosyl-(1→ 3)]-α-L-arabinopyranosyl)oxy]-21-(β-D-glu copyranosyloxy)-20,24S -dihydroxy |
| 19 ^n^ | 3.57 | 945.5030 | -H | C_47_H_78_O_19_ | -2.9 | -2.9 | 329.11  (991) | neg: 991.5049, 945.5030, 783.4520, 637.3944, 505.3527  pos: 964.5394, 471.3481, 453.3368, 435.3275 |  |  | + | E-3+OH+Glc+Rha+Xyl |
| 20 | 3.57 | 1091.5629 | -H | C_53_H_88_O_23_ | -0.9 | -0.8 | 344.9  (1137) | neg: 1137.5620, 1091.5629, 929.5099, 767.4523, 667.3640, 521.3230, 389.2726 |  | + |  | Gypenoside VN4 |
| 21 ^n^ | 3.62 | 1221.5847 | -H | C_58_H_94_O_27_ | -5.7 | -4.7 | 355.93  (1267) | neg: 1267.5878, 1221.5847, 1091.5662, 813.4258, 681.3834, 535.3276, 403.2819 |  | + |  | D-3+Ara+2Rha+Glc+Xyl+O |
| 22 ^n^ | 3.63 | 989.4930 | -H | C_48_H_78_O_21_ | -2.7 | -2.7 | 318.90  (1035) | neg: 1035.4952, 989.4930, 857.4506, 711.3973, 549.3422, 505.3603, 461.2890 | + |  |  | E-1+Glc+Rha+Xyl+Ac |
| 23 | 3.73 | 961.5336 | -H | C_48_H_82_O_19_ | -3.6 | -3.7 | 360.82  (1007) | neg: 1007.5372, 961.5336, 799.4842, 653.4282, 491.3760 | + |  |  | Neoalsoside A5 |
| 24 | 3.92 | 1075.5311 | -H | C_52_H_84_O_23_ | -1.4 | -1.3 | 332.76  (1121) | neg: 1121.5299, 1075.5311, 929.5085, 749.4478, 681.3855, 535.3286, 403.3102 |  | + |  | Dammar-23-en-19-al, 3-[O-6-rhamnopyra-nosyl-(1→2)-O-[β-D-xylopyranosyl-(1→ 3)]-α-L-arabinopyranosyl)oxy]-21-(β-D-glucopyranosyloxy)-20,25-dihydroxy-12-oxo-, (3β,23E)- (ACI) |
| 25 ^n^ | 3.93 | 1061.5527 | -H | C_52_H_86_O_22_ | -0.5 | -0.5 | 341.13  (1107) | neg: 1107.5518, 1061.5527, 929.5057, 767.4561, 681.3853, 535.3243, 403.2883  pos: 1080.5938, 455.3527, 437.3430 | + | + | + | D-3+Ara+Rha+Xyl+Glc |
| 26 ^a^ | 4.05 | 915.4930 | -H | C_46_H_76_O_18_ | -2.3 | -2.5 | 320.09  (961) | neg: 961.4935, 915.4930, 783.4503, 637.3945, 505.3540 |  |  | + | JGL-19 |
| 27 | 4.07 | 1079.5243 | +HCOO | C_50_H_82_O_22_ | -3.1 | -2.9 | 331.16  (1079) | neg: 1079.5243, 1033.5167, 901.4758, 755.4616, 593.3712  pos: 1052.5621, 575.3585, 413.3052 |  | + | + | Abutiloside E |
| 28 | 4.09 | 1063.5641 | -H | C_52_H_88_O_22_ | -4.8 | -4.5 | 343.1  (1109) | neg: 1109.5682, 1063.5641, 931.5228, 769.4722, 623.4152, 491.3752  pos: 1082.6085, 457.3698, 439.3574, 421.3467 | + |  | + | gypenoside 6 |
| 29 | 4.18 | 1063.5635 | -H | C_52_H_88_O_22_ | -5.4 | -5.1 | 343.77  (1109) | neg: 1109.5677, 1063.5635, 931.5220, 769.4722, 637.3953, 491.3725 | + | + |  | gypenoside 6 |
| 30 ^a^ | 4.24 | 915.4911 | -H | C_46_H_76_O_18_ | -4.2 | -4.6 | 341.01  (961) | neg: 961.4960, 915.4911, 783.4504, 637.3949, 505.2698  pos: 934.5361, 489.3579, 471.3477 |  |  | + | JGL-20 |
| 31 ^n^ | 4.29 | 855.4359 | -H | C_43_H_68_O_17_ | -1.9 | -2.2 | 314.52  (901) | neg: 901.4386, 855.4359, 825.4247, 693.3850, 547.3275, 415.2853, 387.2905  pos: 874.479, 429.3003 |  |  | + | E-1−C_3_H_8_O+Ara+Rha+Xyl |
| 32 ^a^ | 4.30 | 943.4856 | +HCOO | C_46_H_74_O_17_ | -4.7 | -5 | 341.47  (943) | neg: 943.4856, 897.4795, 765.4378, 619.3804, 487.3362 |  | + |  | JGL-18 |
| 33 | 4.30 | 1061.5491 | -H | C_52_H_86_O_22_ | -4.1 | -3.9 | 337.36  (1107) | neg: 1107.5516, 1061.5491, 929.5095, 767.4550, 749.4459, 603.3942, 547.3273, 415.2845  pos: 1080.5977, 455.3426, 437.3426 | + | + |  | gypenoside S4 |
| 34 | 4.41 | 1033.5186 | -H | C_50_H_82_O_22_ | -3.3 | -3.2 | 329.17  (1079) | neg: 1079.5212, 1033.5186, 901.4780, 739.4270, 593.3688, 507.3973  pos: 1052.5612, 413.3059, 395.2996 |  | + |  | Abutiloside E |
| 35 | 4.49 | 929.5067 | -H | C_47_H_78_O_18_ | -4.3 | -4.6 | 346.81  (975) | neg: 975.5090, 929.5067, 797.4669, 651.4100 |  |  | + | Gypenoside VN6 |
| 36 ^n^ | 4.51 | 959.5222 | +HCOO | C_47_H_78_O_17_ | 0.6 | 0.6 | 327.57  (959) | neg: 959.5222, 751.4651, 605.4009, 473.3505 |  |  | + | A-1+Ara+Rha+Glc |
| 37 | 4.51 | 1239.6334 | -H | C_59_H_100_O_27_ | -4 | -3.2 | 370.96  (1285) | neg: 1285.6360, 1239.6334, 1107.5862, 945.5321, 799.4937 | + |  |  | Gypenoside CI |
| 38 | 4.52 | 1209.6228 | -H | C_58_H_98_O_26_ | -4 | -3.3 | 358.32  (1255) | neg: 1255.6245, 1209.6228, 1063.5621, 931.5247, 769.4703, 685.3853, 523.3692 | + | + |  | (20S)-3β,20, 21-trihydroxydammar-24-ene-19-oxo-3-O-[α-L-rhamnopyranosyl-(1→ 2)][β-D-xylopyranosyl(1→3)]-α-L-arabino-pyranosyl-21-O-α-L-rhamnopyranosyl-(1 →6)-β-D-glucopyranoside |
| 39 ^a^ | 4.54 | 903.5323 | -H | C_46_H_80_O_17_ | 0.6 | 0.7 | 314.69  (949) | neg: 949.5311, 903.5323, 771.4891, 625.4313, 493.3921, 375.2881  pos: 459.3841, 441.3737, 423.3646 | + | + |  | JGL-22 |
| 40 ^a^ | 4.65 | 1061.5504 | -H | C_52_H_86_O_22_ | -2.8 | -2.6 | 338.78  (1107) | neg: 1107.5522, 1061.5504, 929.5084, 783.4513, 621.4025, 489.3556  pos: 1080.5940, 455.3527 | + | + | + | JGL-16 |
| 41 ^a^ | 4.73 | 929.5087 | -H | C_47_H_78_O_18_ | -4.8 | -5.2 | 317.38  (975) | neg: 975.5126, 929.5087, 797.4684, 651.4118, 489.3591, 405.3016 |  |  | + | JGL-6 |
| 42 ^a^ | 4.92 | 1061.6140 | -H | C_52_H_86_O_22_ | -2.5 | -2.4 | 341.82  (1107) | neg: 1107.5529, 1061.6140, 929.5077, 767.4573, 681.3853, 535.3289, 403.2845  pos: 1080.5978, 455.3523, 437.3441 | + | + | + | JGL-3 |
| 43 | 4.98 | 941.4323 | -H | C_46_H_70_O_20_ | -5.9 | -6.3 | 308.91  (941) | neg: 941.4323, 825.4277, 693.3837, 547.3270, 415.2846, 387.2893  pos: 960.4801, 429.2988 |  |  | + | isomer of cucurbitacin L 2-O-[α-L-rham-nopyranosyl(1→2)][α-L-arabinopyranosyl (1→3)]-β-glucopyranoside |
| 44 | 5.12 | 1077.5803 | -H | C_53_H_90_O_22_ | -4.2 | -3.9 | 356.35  (1123) | neg: 1123.5823, 1077.5803, 945.5353, 765.4775, 619.4205 | + | + |  | Isomer of JGL-14 |
| 45 ^n^ | 5.23 | 855.4344 | -H | C_43_H_68_O_17_ | -3.4 | -4 | 317.14  (901) | neg: 901.4385, 855.4344, 723.3953, 577.3391, 445.2970, 415.2856, 403.2842 |  |  | + | E-1−C_3_H_8_O+Ara+Rha+Xyl |
| 46 ^a^ | 5.27 | 943.4954 | +HCOO | C_46_H_74_O_17_ | -3.7 | -4.1 | 333.32  (943) | neg: 943.4954, 897.4811, 765.4415, 619.3846, 487.3429, 403.2850  pos: 471.3468, 453.3362, 435.3254, 417.3149 | + | + | + | JGL-1 |
| 47 ^a^ | 5.29 | 899.4985 | -H | C_46_H_76_O_17_ | -1.9 | -2.1 | 312.74  (945) | neg: 945.4996, 899.4985, 767.4568, 621.4026, 521.3121, 389.2693 | + | + | + | JGL-7 |
| 48 | 5.33 | 959.5170 | -H | C_48_H_80_O_19_ | -4.6 | -4.8 | 353.54  (1005) | neg: 1005.5203, 959.5170, 797.4646, 651.4141, 489.3561 |  |  | + | Dammar-25-en-24-one, 6-[2-O-rhamnopy-ranosyl-β-D-glucopyranosyl]oxy]-20-(β-D-glucopyranosyloxy)-3,12-dihydroxy-, (3β,6α,12β,20S)- (ACI) |
| 49 | 5.50 | 1077.5796 | -H | C_53_H_90_O_22_ | -4.9 | -4.5 | 352.35  (1123) | neg: 1123.5840, 1077.5796, 945.5380, 845.4538, 699.3924, 537.3434, 375.2888 | + | + |  | Isomer of JGL-14 |
| 50 | 5.55 | 1075.5640 | -H | C_53_H_88_O_22_ | -4.9 | -4.6 | 350.48  (1121) | neg: 1121.5684, 1075.5640, 943.5230, 781.4720, 667.3699, 521.3119, 389.2691  pos: 1094.6095, 455.3524, 437.3418, 419.3314 | + | + | + | gypenoside S3 |
| 51 | 5.57 | 1047.5665 | -H | C_52_H_88_O_21_ | -7.5 | -7.2 | 343.31  (1093) | neg: 1093.5714, 1047.5665, 915.5260, 753.4752, 653.3915, 521.3423, 389.2686 |  |  | + | isomer of β-D-glucopyranoside, (3β)-3-[O-α-L-rhamnopyranosyl-(1→2)-O-[β-D-xylo-pyranosyl-(1→3)]-α-L-arabinopyranosyl) oxy]-19,20-dihydroxydammar-24-en-21-yl |
| 52 | 5.63 | 945.5003 | +HCOO | C_46_H_76_O_17_ | -5.6 | -5.9 | 326.83  (945) | neg: 945.5003, 899.4930, 767.4570, 621.3993, 489.3586, 403.2856, 389.2698  pos: 918.5415, 473.3619, 455.3521, 437.343 |  | + | + | 18-Norandrostan-19-al, 3-[O-α-L-rhamno-pyranosyl-(1→2)-O-[β-D-xylopyranosyl-(1→3)]-α-L-arabinopyranosyl)oxy]-17-[1,2-dihydroxy-3-(1-hydroxy-1-methyle thyl)cyclopentyl]-, (3β,5α,17β)- (9CI) |
| 53 ^a^ | 5.67 | 913.4750 | -H | C_46_H_74_O_18_ | -4.7 | -5.1 | 345.93  (959) | neg: 959.4786, 913.4750, 781.4359, 681.3843, 535.3276, 403.2851, 375.2893  pos: 469.3315, 451.3211, 433.3104 | + | + | + | JGL-4 |
| 54 | 5.77 | 1077.5807 | -H | C_53_H_90_O_22_ | -3.8 | -3.5 | 351.08  (1123) | neg: 1123.5839, 1077.5807, 945.5372, 783.4782, 683.3994, 537.3443, 375.2919 | + |  |  | Isomer of JGL-14 |
| 55 ^n^ | 5.79 | 915.4945 | -H | C_46_H_76_O_18_ | -0.8 | -0.9 | 348.22  (961) | neg: 961.4950, 915.4945, 783.4506, 683.4000, 537.3430, 405.3006, 375.2911  pos: 469.3315, 451.3211, 433.3104 | + | + | + | B-6+OH+Ara+Rha+Xyl |
| 56 | 5.90 | 1163.5786 | -H | C_56_H_92_O_25_ | -6.3 | -5.4 | 358.94  (1163) | neg: 1163.5786, 1077.5820, 945.5387, 799.4843, 681.3860, 535.3298, 373.2754 | + |  |  | Malonylfloralginsenoside Rc2 |
| 57 | 5.97 | 941.4330 | -H | C_46_H_70_O_20_ | -5.2 | -5.5 | 321.09  (941) | neg: 941.4330, 897.4395, 855.4346, 767.4598, 621.4016, 489.3592 |  |  | + | isomer of cucurbitacin L 2-O-[α-L-rhamno-pyranosyl(1→2)][α-L-arabinopyranosyl(1 →3)]-β-glucopyranoside |
| 58 ^n^ | 6.00 | 911.4631 | -H | C_46_H_72_O_18_ | -0.9 | -1 | 335.37  (957) | neg: 957.4656, 911.4631, 779.4203, 633.3654, 501.3220, 489.3585, 403.2840 | + |  |  | C-2+OH+Ara+Rha+Xyl |
| 59 | 6.04 | 899.4985 | -H | C_46_H_76_O_17_ | -1.9 | -2.1 | 318.70  (945) | neg: 945.5005, 899.4985, 767.4564, 621.3995, 489.3583, 403.2854, 389.2714  pos: 918.5423, 473.3643, 455.3523, 437.3422 | + | + | + | isomer of 18-Norandrostan-19-al, 3-[O-α-L- rhamnopyranosyl-(1→2)-O-[β-D-xylo-pyranosyl-(1→3)]-α-L-arabinopyranosyl) oxy]-17-[1,2-dihydroxy-3-(1-hydroxy-1-methylethyl)cyclopentyl]-, (3β,5α,17β) |
| 60 ^n^ | 6.15 | 813.4242 | -H | C_40_H_64_O_14_ | -3.1 | -3.8 | 288.34  (813) | neg: 813.4242, 681.3864, 535.3278, 403.2852  pos: 786.4652 |  |  | + | B-3−CH_3_+OH+Ara+Rha |
| 61 | 6.23 | 875.4597 | +HCOO | C_42_H_70_O_16_ | -4.3 | -4.9 | 288.20  (875) | neg: 875.4597, 829.4821, 667.4058, 535.3286, 489.3591, 403.2852  pos: 848.5003, 471.3474, 453.3361, 435.3281 |  |  | + | Gentirigeoside E |
| 62 | 6.33 | 827.4393 | +HCOO | C_41_H_66_O_14_ | -3.6 | -4.4 | 296.28  (827) | neg: 827.4393, 781.4292, 765.4391, 681.3861, 535.3261  pos: 487.3444, 469.3321, 451.3231 |  | + | + | gypenoside UL2 |
| 63 ^n^ | 6.39 | 985.4948 | -H | C_49_H_78_O_20_ | -6 | -6.1 | 315.66  (985) | neg: 985.4948, 941.4980, 899.4995, 767.4566, 621.4019, 489.3585 |  | + | + | A-2+Ara+Rha+Xyl+Mal |
| 64 | 6.44 | 1239.6324 | -H | C_59_H_100_O_28_ | -5 | -4 | 371.49  (1285) | neg: 1285.6356, 1239.6324, 1093.5791, 929.5092, 797.4661, 651.3685, 489.3588 | + |  |  | Gypenoside XC |
| 65 ^a^ | 6.47 | 1207.6076 | -H | C_58_H_96_O_26_ | -3.6 | -3 | 354.77  (1253) | neg: 1253.6080, 1207.6076, 1075.6390, 913.5717, 751.5070, 681.4368, 535.3553, 403.3050, 375.3147  pos: 1226.6514, 439.3590, 421.3478 | + | + |  | JGL-8 |
| 66 ^n^ | 6.48 | 999.4738 | -H | C_49_H_76_O_21_ | -6.3 | -6.3 | 349.13  (999) | neg: 999.4738, 955.4850, 913.4761, 781.4363, 681.3839, 535.3274, 403.2844, 375.2896  pos: 1018.5220, 469.3318, 451.3213, 433.3117 | + | + | + | B-4+Ara+Rha+Xyl+Mal |
| 67 | 6.51 | 915.4921 | -H | C_46_H_76_O_18_ | -3.2 | -3.5 | 316.10  (961) | neg: 961.4941, 915.4921, 783.4523, 667.3689, 521.3118, 389.2690 |  |  | + | Dammar-23-en-19-al,3-[O-α-L-rhamnopy-ranosyl-(1→2)-O-[β-D-xylopyranosyl-(1→3)]-α-L-arabinopyranosyl)oxy]-25-hydroperoxy-20,21-dihydroxy-, (3β,23E) |
| 68 ^n^ | 6.53 | 767.4553 | -H | C_41_H_68_O_13_ | -2.9 | -3.8 | 287.13  (813) | neg: 813.4608, 767.4553, 621.3992, 489.3583, 389.2693  pos: 786.4989, 619.4222, 455.3524, 437.3427 |  | + | + | D-3+Rha+Xyl |
| 69 ^n^ | 6.63 | 955.4865 | -H | C_48_H_76_O_19_ | -3.8 | -4 | 430.13  (1001) | neg: 1001.4890, 955.4865, 913.4785, 781.4370, 681.3842, 535.3275, 403.2846, 375.2898  pos: 469.3317, 451.3221 | + | + |  | B-4+Ara+Rha+Xyl+Ac |
| 70 ^a^ | 6.69 | 1209.6224 | -H | C_58_H_98_O_26_ | -4.4 | -3.6 | 355.13  (1255) | neg: 1255.6241, 1209.6224, 1077.5802, 915.5255, 753.4739, 607.4354  pos: 1211.6404, 423.3618 | + | + |  | JGL-11 |
| 71 ^n^ | 6.75 | 999.4738 | -H | C_49_H_76_O_21_ | -6.3 | -6.3 | 331.27  (999) | neg: 999.4738, 955.4867, 913.4776, 767.4579, 681.3840, 535.3278, 403.2857, 375.2899  pos: 1018.5209, 469.3315, 451.3215 | + |  | + | B-3−CH_3_+OH+Ara+Rha+Rha+Mal |
| 72 ^n^ | 6.75 | 985.4943 | -H | C_49_H_78_O_20_ | -4.2 | -4.3 | 311.10  (985) | neg: 985.4943, 899.4962, 767.4554, 621.3994, 489.3581, 403.2851, 389.2693  pos: 1004.5484, 473.3648, 455.3527, 437.3407 |  |  | + | D-3+Ara+Rha+Xyl+Mal |
| 73 ^n^ | 6.75 | 1015.5074 | -H | C_50_H_80_O_21_ | -4 | -3.9 | 339.40  (1015) | neg: 1015.5074, 929.4987, 797.4656, 651.4092, 489.3572 |  | + | + | A-2+Glc+Rha+Xyl+Mal |
| 74 ^n^ | 6.76 | 797.4656 | -H | C_42_H_70_O_14_ | -3.1 | -3.9 | 298.69  (843) | neg: 843.4386, 797.4656, 651.4092, 489.3572  pos: 453.3372, 435.3271 |  |  | + | E-7+Glc+Rha |
| 75 ^n^ | 6.78 | 941.5074 | -H | C_48_H_78_O_18_ | -3.6 | -3.8 | 334.07  (987) | neg: 987.5085, 941.5074, 899.4962, 767.4554, 621.3994, 489.3581, 389.2693  pos: 473.3646, 455.3528, 437.3426 |  |  | + | D-3+Ara+Rha+Xyl+Ac |
| 76 ^n^ | 6.97 | 767.4567 | -H | C_41_H_68_O_13_ | -1.5 | -2 | 287.85  (813) | neg: 813.4599, 767.4567, 621.4028, 489.3578, 389.2687  pos: 786.5009, 619.4227, 473.3689, 455.3526, 437.3428 |  | + | + | D-3+Ara+Rha |
| 77 | 7.09 | 899.4965 | -H | C_46_H_76_O_17_ | -3.9 | -4.3 | 332.00  (945) | neg: 945.5007, 899.4965, 767.4565, 621.4010, 489.3589, 403.2859  pos: 455.3524, 437.342 | + | + | + | isomer of 18-Norandrostan-19-al, 3-[O-α-L-rhamnopyranosyl-(1→2)-O-[β-D-xylo-pyranosyl-(1→3)]-α-L-arabinopyranosyl) oxy]-17-[1,2-dihydroxy-3-(1-hydroxy-1-methylethyl)cyclopentyl]-, (3β,5α,17β) |
| 78 ^n^ | 7.09 | 941.5129 | -H | C_48_H_78_O_18_ | 1.9 | 2 | 325.62  (987) | neg: 987.5086, 941.5129, 899.4979, 767.4559, 621.4001, 489.3587, 403.2823 |  |  | + | D-3+Ara+Rha+Xyl+Ac |
| 79 ^n^ | 7.11 | 829.4553 | +HCOO | C_41_H_68_O_14_ | -3.3 | -4 | 287.48  (829) | neg: 829.4553, 765.4413, 619.3838, 487.3423  pos: 802.4944, 767.4583, 635.4155, 489.3581, 471.3474, 453.337 |  |  | + | E-1+Xyl+Rha |
| 80 ^n^ | 7.11 | 955.4882 | -H | C_48_H_76_O_19_ | -2.1 | -2.2 | 353.19  (1001) | neg: 1001.4891, 955.4882, 913.4777, 767.4565, 621.4007, 489.3588, 403.2846, 375.2911  pos: 473.3636, 455.352, 437.3421, 419.3325 |  | + | + | C-1+2OH−CH_3_+Ara+2Rha+Ac |
| 81 ^n^ | 7.16 | 985.4976 | -H | C_49_H_78_O_20_ | -3.2 | -3.2 | 308.93  (985) | neg: 985.4976, 941.5068, 899.4964, 767.4561, 621.3995, 489.3584  pos: 1004.5449, 455.3521, 437.3423 |  |  | + | A-2+Ara+Rha+Xyl+Mal |
| 82 ^n^ | 7.25 | 769.4431 | -H | C_40_H_66_O_14_ | 5.7 | 7.4 | 284.45  (815) | neg: 815.4399, 769.4431, 667.3959, 521.3127, 389.2696, 359.2590  pos: 788.4849, 471.3454, 437.3412 |  |  | + | B-1+2OH−CH_3_+Ara+Rha |
| 83 | 7.34 | 1043.5461 | -H | C_52_H_84_O_21_ | 3.4 | 3.3 | 337.25  (1089) | neg: 1089.5406, 1043.5461, 911.5025, 829.4209, 667.4029, 521.3473, 389.3051  pos: 1062.5807, 455.3527, 437.3421, 419.3329 |  |  | + | Dammara-23,25-dien-19-al, 3-[O-α-L-rha-mnopyranosyl-(1→2)-O-[β-D-xylopyran-osyl-(1→3)]-α-L-arabinopyranosyl)oxy]-21-(β-D-glucopyranosyloxy)-20-hydroxy-, (3β,23E)- (ACI) |
| 84 | 7.34 | 1091.5953 | -H | C_54_H_92_O_22_ | -4.9 | -4.5 | 364.46  (1137) | neg: 1137.5991, 1091.5953, 959.5535, 797.5030, 667.4044, 521.3474, 389.3048 | + |  |  | β-D-Glucopyranoside, (3β)-20-[6-O-α-L-rhamnopyranosyl-β-D-glucopyranosyl] oxy]-19-hydroxydammar-24-en-3-yl 2-O-β-D-glucopyranosyl- (9CI) |
| 85 | 7.40 | 899.4984 | -H | C_46_H_76_O_17_ | -2 | -2.2 | 351.79  (945) | neg: 945.5012, 899.4984, 767.4587, 667.4046, 521.3478, 389.3059 | + | + | + | isomer of 18-Norandrostan-19-al, 3-[(O-α-L-rhamnopyranosyl-(1→2)-O-[β-D-xylo-pyranosyl-(1→3)]-α-L-arabinopyranosyl) oxy]-17-[1,2-dihydroxy-3-(1-hydroxy-1-methylethyl)cyclopentyl]-, (3β,5α,17β) |
| 86 | 7.50 | 1061.5851 | -H | C_53_H_90_O_21_ | -4.5 | -4.2 | 350.33  (1107) | neg: 1107.5894, 1061.5851, 929.5407, 767.4894, 667.4056, 521.3476, 389.3050  pos: 455.3527, 437.3429 | + |  |  | 3β,20S,21-trihydroxy-25-methoxydammar-23-ene 3-O-α-L-rhamnopyranosyl (1→2)-[β-D-glucopyranosyl(1→3)]-β-D-arabino-pyranosyl-21-O-β-D-xylopyranoside |
| 87 ^n^ | 7.54 | 857.4510 | -H | C_43_H_70_O_17_ | -2.5 | -2.9 | 328.11  (903) | neg: 903.4543, 857.4510, 725.4119, 681.3841, 535.3273, 403.32849, 375.2894 |  |  | + | B-3+Ara+Rha+Xyl−C_3_H_4_ |
| 88 ^n^ | 7.55 | 783.4504 | -H | C_41_H_68_O_14_ | -2.7 | -3.4 | 286.64  (829) | neg: 829.4548,783.4504, 667.3687, 521.3110, 389.2684, 359.2778  pos: 802.4932, 455.3552, 437.3428, 419.3333 |  |  | + | D-3+Xyl+Rha+OH |
| 89 ^n^ | 7.57 | 941.5143 | -H | C_48_H_78_O_18_ | 3.3 | 3.5 | 324.51  (987) | neg: 987.5088, 941.5143, 899.4974, 767.4568, 621.4000, 489.3586 |  |  | + | D-3+Ara+Rha+Xyl+Ac |
| 90 | 7.57 | 1075.5638 | -H | C_53_H_88_O_22_ | -5.1 | -4.7 | 342.11  (1121) | neg: 1121.5682, 1075.5638, 913.5112, 751.4637, 667.4048, 521.3486, 389.3059 | + |  |  | Gypenoside VN2 |
| 91 | 7.60 | 913.4753 | -H | C_46_H_74_O_18_ | -4.4 | -4.8 | 338.70  (959) | neg: 959.4793, 913.4753, 813. 4264, 681.3848, 535.3272, 403.2851, 375.2900  pos: 469.3314, 451.3211, 433.3113 |  | + | + | (21R,23R)-3β,20ξ,21,26-Tetrahydroxy-19-oxo-21,23-epoxydammar-24-ene 3-O-[α-L-rhamnopyranosyl-(1→2)][β-D-xylopyran-osyl-(1→3)]-α-L-arabinopyranoside |
| 92 ^a^ | 7.63 | 1237.6160 | -H | C_59_H_98_O_27_ | -5.7 | -4.6 | 379.19  (1283) | neg: 1283.6179, 1237.6160, 1091.5608, 959.5090, 389.3063 |  | + |  | JGL-10 |
| 93 | 7.66 | 927.4929 | -H | C_47_H_76_O_18_ | -2.4 | -2.6 | 355.57  (973) | neg: 973.4946, 927.4929, 795.4511, 681.3845, 535. 3273, 403.2856, 375.2899  pos: 469.3315, 451.321 | + | + | + | Dammar-24-en-21-al, 3-[O-α-L-rhamno-pyranosyl-(1→2)-O-[β-D-glucopyranosyl-(1→3)]-α-L-arabinopyranosyl)oxy]-20,23-dihydroxy-12-oxo-, cyclic 21,23-hemi-acetal, (3β,20ξ,21S,23R)- (ACI) |
| 94 ^a^ | 7.69 | 1077.5787 | -H | C_53_H_90_O_22_ | -5.8 | -5.4 | 344.10  (1123) | neg: 1123.5834, 1077.5787, 915.5257, 783.4534, 637.3967, 475.3789 | + |  |  | JGL-2 |
| 95 ^n^ | 7.75 | 915.4954 | -H | C_46_H_76_O_18_ | 0.1 | 0.1 | 342.75  (961) | neg: 961.4957, 915.4954, 783.4520, 637.3953, 505.3529, 389.3052 |  | + | + | B-6+OH+Ara+Rha+Xyl |
| 96 | 7.78 | 899.4977 | -H | C_46_H_76_O_17_ | -2.7 | -3 | 337.39  (945) | neg: 945.5004, 899.4977, 767.4551, 621.3990, 489.3578, 403.2848  pos: 918.5416, 473.363, 455.3524, 437.343 |  |  | + | isomer of 18-Norandrostan-19-al, 3-[O-α-L-rhamnopyranosyl-(1→2)-O-[β-D-xylo-pyranosyl-(1→3)]-α-L-arabinopyranosyl) oxy]-17-[1,2-dihydroxy-3-(1-hydroxy-1-methylethyl)cyclopentyl]-, (3β,5α,17β) |
| 97 ^n^ | 7.81 | 985.4955 | -H | C_49_H_78_O_20_ | -5.3 | -5.4 | 322.69  (985) | neg: 985.4955, 941.5060, 899.4978, 767.4565, 621.3998, 489.3583  pos: 1004.5445, 455.3528, 437.3424, 419.3335 | + | + | + | A-2+Ara+Rha+Xyl+Mal |
| 98 ^a^ | 7.84 | 1253.6488 | -H | C_60_H_102_O_27_ | -4.2 | -3.4 | 374.32  (1299) | neg: 1299.6508, 1253.6488, 1091.5989, 929.5423, 767.4583, 621.4062, 459.3743 | + |  |  | JGL-13 |
| 99 ^n^ | 7.90 | 931.5211 | +HCOO | C_46_H_78_O_16_ | -5.5 | -5.9 | 332.95  (931) | neg: 931.5211, 885.5142, 753.4790, 607.4225, 475.3802, 375.2896 |  |  | + | E-2+Ara+Rha+Xyl |
| 100 ^n^ | 7.90 | 753.4401 | -H | C_40_H_66_O_13_ | -2.4 | -3.2 | 279.84  (799) | neg: 799.4445, 753.4401, 621.3972, 489.3581, 403.2817, 389.2844 |  | + | + | D-3+Ara+Xyl |
| 101 | 8.00 | 1075.5649 | -H | C_53_H_88_O_22_ | -4 | -3.7 | 351.14  (1121) | neg: 1121.5682, 1075.5649, 943.5229, 797.4674, 635.4164, 473.3627, 389.3031  pos: 1094.6105, 439.3576, 421.3470 | + |  |  | 3β,20,21-trihydroxydammar-24-ene-19-oxo -3-O-[α-L-rhamnopyranosyl(1→2)][β-D-xylopyranosyl(1→3)]- β-D-glucopyrano-side-21-O-β-D-glucopyranoside |
| 102 ^n^ | 8.03 | 855.4712 | +HCOO | C_43_H_70_O_14_ | -3 | -3.5 | 297.79  (855) | neg: 855.4712, 809.4613, 767.4568, 621.4011, 489.3583, 389.2688  pos: 455.3535, 437.3419 |  |  | + | D-3+Ara+Rha+Ac |
| 103 | 8.16 | 927.4929 | -H | C_47_H_76_O_18_ | -2.4 | -2.6 | 354.98  (973) | neg: 973.4966, 927.4929, 795.4510, 649.3928, 487.3429, 389.2691, 359.2993  pos: 469.3315, 451.3268 | + |  |  | Dammar-24-en-21-al, 3-[(O-α-L-rhamno-pyranosyl-(1→2)-O-[β-D-glucopyranosyl-(1→3)]-α-L-arabinopyranosyl)oxy]-20,23-dihydroxy-12-oxo-, cyclic 21,23-hemiacetal, (3β,20ξ,21S,23R)- (ACI) |
| 104 | 8.18 | 871.4696 | -H | C_44_H_72_O_17_ | 0.5 | 0.6 | 329.61  (917) | neg: 917.4695, 871.4696, 739.4260, 593.3967, 535.3489, 403.2857, 389.3057 |  | + | + | β-D-glucopyranoside, (3β,12β,23E)-3-[(2-carboxyacetyl)oxy]-25-hydroperoxy-12-hydroxydammar-23-en-20-yl 6-O-α-L-arabinopyranosyl- (ACI) |
| 105 | 8.20 | 871.4656 | -H | C_44_H_72_O_17_ | -3.5 | -4 | 310.86  (871) | neg: 871.4656, 767.4564, 621.4001, 489.3581, 389.2698, 359.2591 |  |  | + | Notoginsenoside NL-A4 |
| 106 ^a^ | 8.25 | 1077.5801 | -H | C_53_H_90_O_22_ | -4.4 | -4.1 | 351.83  (1123) | neg: 1123.5842, 1077.5801, 945.5388, 799.4822, 637.4310, 475.3801, 389.3056  pos: 1079.5988, 441.3730, 423.3626 | + |  |  | GL-11 |
| 107 ^n^ | 8.28 | 985.4952 | -H | C_49_H_78_O_20_ | -5.6 | -5.7 | 360.51  (985) | neg: 985.4952, 941.5099, 899.4923, 767.4583, 667.4050, 521.3483, 389.3056 |  | + | + | D-3+Ara+Rha+Xyl+Mal |
| 108 ^n^ | 8.30 | 885.5164 | -H | C_46_H_78_O_16_ | -4.8 | -5.4 | 331.33  (931) | neg: 931.5201, 885.5164, 753.4786, 607.4209, 475.3797, 389.3064, 375.2917 |  |  | + | E-2+Ara+Rha+Xyl |
| 109 | 8.31 | 767.4537 | -H | C_41_H_68_O_13_ | -4.5 | -5.9 | 286.15  (813) | neg: 813.4600, 767.4537, 621.4000, 489.3583  pos: 455.3528, 437.3425 | + | + | + | A-2+Ara+Rha |
| 110 | 8.40 | 899.4982 | -H | C_46_H_76_O_17_ | -2.2 | -2.4 | 337.74  (945) | neg: 945.5000, 899.4982, 767.4559, 621.4009, 489.3576, 389.3070  pos: 918.5424, 473.3629, 455.3521, 437.3431 | + | + | + | isomer of 18-Norandrostan-19-al, 3-[(O-α-L-rhamnopyranosyl-(1→2)-O-[β-D-xylopy -ranosyl-(1→3)]-α-L-arabinopyranosyl) oxy]-17-[1,2-dihydroxy-3-(1-hydroxy-1-methylethyl)cyclopentyl]-, (3β,5α,17β) |
| 111 | 8.44 | 1223.6366 | -H | C_59_H_100_O_26_ | -5.9 | -4.8 | 374.35  (1269) | neg: 1269.6412, 1223.6366, 1091.5977, 1045.5533, 913.5126, 751.4611, 605.4044, 473.3643 | + | + |  | 3β,20S,21-trihydroxydammar-24-ene-3-O-α- L-rhamnopyranosyl(1→ 2) [β-D-gluco-pyranosyl(1→ 3)] [β-D-xylopyranosyl(1→ 6)]-β-D-glucopyranosyl-20-O-β-D-glucopyranoside |
| 112 | 8.45 | 897.4813 | -H | C_46_H_74_O_17_ | -3.5 | -3.9 | 346.41  (943) | neg: 943.4857, 897.4813, 765.4418, 619.3843, 487.3323, 389.2690  pos: 453.3371, 435.3266 | + |  |  | isomer of Gypenoside A |
| 113 ^n^ | 8.46 | 941.5056 | -H | C_48_H_78_O_18_ | -5.4 | -5.7 | 339.72  (987) | neg: 987.5080, 941.5056, 899.4894, 767.4568, 621.3979, 489.3584, 403.2843  pos: 473.3633, 455.3523, 437.3423 |  |  | + | D-3+Ara+Rha+Xyl+Ac |
| 114 | 8.51 | 1193.6285 | -H | C_58_H_98_O_25_ | -3.4 | -2.8 | 360  (1239) | neg: 1239.6293, 1193.6285, 1061.5790, 899.5354, 753.4565 |  | + |  | 3β,20S,21-trihydroxydammar-24-ene 3-O-[α-L-rhamnopyranosyl(1→ 2)][β-D-xylo-pyranosyl(1→ 3)]-β-D-glucopyranosyl-20-O-[β-D-xylopyranosyl(1→ 6)]-β-D-glucopyranoside |
| 115 ^n^ | 8.52 | 999.4799 | -H | C_49_H_76_O_21_ | -0.2 | -0.2 | 339.77  (999) | neg: 999.4799, 955.4858, 913.4759, 767.4559, 681.3845, 535.3268, 403.2843, 375.2893  pos: 469.3312, 451.3212 |  |  | + | B-3+OH−CH_3_+Ara+2Rha+Mal |
| 116 ^a^ | 8.53 | 1045.5544 | -H | C_52_H_86_O_21_ | -3.9 | -3.7 | 341.79  (1091) | neg: 1091.5577, 1045.5544, 913.5126, 751.4625, 605.4061, 473.3637, 389.3059  pos: 1064.5981, 439.3573, 421.3467 | + | + | + | GL-20 |
| 117 _n_ | 8.56 | 985.4951 | -H | C_49_H_78_O_20_ | -5.7 | -5.8 | 330.46  (985) | neg: 985.4951, 941.5005, 899.4921, 767.4567, 621.4009, 489.3550, 403.2854 |  |  | + | D-3+Ara+Rha+Xyl+Mal |
| 118 ^n^ | 8.57 | 1013.4924 | -H | C_50_H_78_O_21_ | -3.3 | -3.3 | 363.00  (1013) | neg: 1013.4924, 969.4979, 927.4921, 795.4504, 649.3975, 535.3231, 403.2825  pos: 469.3312 | + |  | + | D-3+CO+Ara+Rha+Xyl+Mal |
| 119 ^n^ | 8.58 | 855.4702 | +HCOO | C_43_H_70_O_14_ | -4 | -4.7 | 302.42  (855) | neg: 855.4702, 767.4581, 621.4017, 489.3580, 403.2845, 389.2681 |  |  | + | D-3+Ara+Rha+Ac |
| 120 ^n^ | 8.61 | 871.4639 | +HCOO | C_43_H_70_O_15_ | -4.6 | -5.3 | 305.78  (871) | neg: 871.4639, 825.4573, 783.4493, 767.4971, 667.3698, 521.3107, 389.2684 |  |  | + | B-6+OH+Ara+Rha+Ac |
| 121 | 8.71 | 739.4246 | -H | C_39_H_64_O_13_ | -2.3 | -3.1 | 296.31  (785) | neg: 785.4296, 739.4246, 431.3139, 389.2667  pos: 413.3055 |  |  | + | isomer of β-D-glucopyranoside, (1β,3β, 16β,22S)-1-[(-α-L-rhamnopyranosyl)oxy]-3,22-dihydroxycholesta-5,24-dien-16-yl |
| 122 ^a^ | 8.71 | 1047.5710 | -H | C_52_H_88_O_21_ | -3 | -2.9 | 341.16  (1093) | neg: 1093.5730, 1047.5710, 915.5277, 753.4771, 607.4211, 475.3781, 391.2858  pos: 1049.5883, 441.3731, 423.3625, 405.3519 | + | + | + | GL-10 |
| 123 ^n^ | 8.73 | 1001.4920 | -H | C_49_H_78_O_21_ | -3.7 | -3.7 | 336.77  (1001) | neg: 1001.4920, 957.4952, 915.5248, 783.4517, 637.3975, 505.3513, 389.3051 |  | + | + | K-6+OH+Ara+Rha+Xyl+Mal |
| 124 ^n^ | 8.75 | 971.5153 | -H | C_49_H_80_O_19_ | -6.3 | -6.5 | 340.58  (971) | neg: 971.5153, 885.5178, 753.4786, 607.4048, 475.3783, 389.3035 |  |  | + | E-2+Ara+Rha+Xyl+Mal |
| 125 | 8.76 | 927.4902 | -H | C_47_H_76_O_18_ | -5.1 | -5.5 | 356.81  (973) | neg: 973.4969, 927.4902, 795.4528, 649.3964, 487.3628 | + |  |  | isomer of JGL-15 |
| 126 ^n^ | 8.87 | 941.5094 | -H | C_48_H_78_O_18_ | -1.6 | -1.7 | 361.45  (987) | neg: 987.5096, 941.5094, 899.5009, 767.4556, 621.4001, 489.3577  pos: 455.3523, 437.3423 |  |  | + | D-3+Ara+Rha+Xyl+Ac |
| 127 ^n^ | 8.91 | 1145.5399 | -H | C_55_H_86_O_25_ | 1.9 | 1.7 | 338.81  (1145) | neg: 1145.5399, 1059.5323, 897.4803, 765.4412, 681.3853, 535.3265, 403.2856 |  | + | + | A-3+Glc+Rha+2Xyl+Mal |
| 128 | 8.96 | 713.4098 | +HCOO | C_36_H_60_O_11_ | -1.4 | -2 | 262.23  (713) | neg: 713.4098, 505.3533, 359.2596 |  |  | + | Dammaran-21-oic acid, 3-(β-D-glucopyra-nosyl-oxy)-20,23,24,25-tetrahydroxy-, γ-lactone, (3β,23S,24R)- (ACI) |
| 129 ^n^ | 9.02 | 999.4764 | -H | C_49_H_76_O_21_ | -3.7 | -3.7 | 323.95  (999) | neg: 999.4764, 957.4640, 795.4246, 681.3846, 535.3275, 403.2846, 375.2903  pos: 1018.5202, 469.3313, 451.3220 |  | + | + | B-4+Glc+Rha+Ara+Ac |
| 130 ^n^ | 9.09 | 957.4642 | -H | C_47_H_74_O_20_ | -5.3 | -5.5 | 339.16  (957) | neg: 957.4642, 913.4783, 871.4655, 739.4256, 593.3691, 431.3156, 403.2857  pos: 413.3054 |  |  | + | B-4+O−H_2_+Glc+Rha+Ara+Ac |
| 131 ^n^ | 9.13 | 903.4536 | +HCOO | C_43_H_70_O_17_ | -3.5 | -3.9 | 326.52  (903) | neg: 903.4536, 857.4463, 725.4096, 579.3531, 447.3113 |  |  | + | B-3+Ara+Rha+Xyl−C_3_H_4_ |
| 132 ^n^ | 9.19 | 1145.5353 | -H | C_55_H_86_O_25_ | -2.9 | -2.4 | 345.17  (1145) | neg: 1145.5353, 983.5125, 939.4912, 807.4510, 765.4405, 681.3834, 535.3275, 403.2836  pos: 1164.5778, 471.3504, 453.3365, 435.3270 |  | + | + | A-3+Glc+Rha+2Xyl+Mal |
| 133 ^n^ | 9.26 | 767.4580 | -H | C_41_H_68_O_13_ | -0.2 | -0.3 | 302.85  (813) | neg: 813.4605, 767.4580, 621.3992  pos: 786.4991, 473.3642, 455.3533 |  |  | + | D-3+Ara+Rha |
| 134 | 9.28 | 871.4658 | -H | C_44_H_72_O_17_ | -3.3 | -3.8 | 331.64  (917) | neg: 917.4691, 871.4658, 739.4255, 593.3699, 535.3259, 403.2816 |  |  | + | β-D-glucopyranoside, (3β,12β,23E)-3-[(2-carboxyacetyl)oxy]-25-hydroperoxy-12-hydroxydammar-23-en-20-yl 6-O-α-L-arabinopyranosyl- (ACI) |
| 135 ^n^ | 9.33 | 983.4819 | -H | C_49_H_76_O_20_ | -3.3 | -3.4 | 354.95  (983) | neg: 983.4819, 939.4921, 897.4844, 765.4410, 619.3846, 487.3427  pos: 1002.5281, 471.3480, 453.3370, 435.3273 | + |  |  | A-3+Ara+Rha+Xyl+Mal |
| 136 ^n^ | 9.35 | 985.4971 | -H | C_49_H_78_O_20_ | -3.7 | -3.8 | 342.17  (985) | neg: 985.4971, 941.5031, 899.4974, 767.4554, 621.4001, 489.3576, 389.3032 | + | + | + | D-3+Ara+Rha+Xyl+Mal |
| 137 ^n^ | 9.38 | 825.4667 | -H | C_43_H_70_O_15_ | -3.1 | -3.8 | 304.71  (871) | neg: 871.4639, 825.4667, 783.4544, 667.3702, 521.3116, 389.2687, 359.2587 |  |  | + | B-6+OH+Ara+Rha+Ac |
| 138 | 9.40 | 929.5141 | +HCOO | C_46_H_76_O_16_ | 3.1 | 3.3 | 317.71  (929) | neg: 929.5141, 883.4982, 751.4612, 605.4016, 535.3276, 403.2826 |  |  | + | Isomer of Gylongiposide I |
| 139 ^n^ | 9.44 | 855.4707 | +HCOO | C_43_H_70_O_14_ | -3.5 | -4.1 | 319.57  (855) | neg: 855.4707, 767.4559, 621.4002, 489.3588 |  |  | + | A-2+Ara+Rha+Ac |
| 140 ^n^ | 9.52 | 1131.5538 | -H | C_55_H_88_O_24_ | -4.9 | -4.3 | 335.92  (1131) | neg: 1131.5538, 1087.5638, 1045.5549, 913.5121, 751.4617, 605.4053, 473.3645, 389.3245  pos: 1150.5986, 439.3573, 421.3465 | + | + | + | F-1+Ara+Rha+Xyl+Mal+Glc |
| 141 | 9.57 | 1075.5656 | -H | C_53_H_88_O_22_ | -3.3 | -3.1 | 354.94  (1121) | neg: 1121.5662, 1075.5656, 913.5129, 751.4621, 605.4072, 473.3604  pos: 1094.6125, 439.3575, 421.3468 |  | + |  | Gypenoside XLVIII |
| 142 | 9.65 | 1045.5538 | -H | C_52_H_86_O_21_ | -4.5 | -4.3 | 341.64  (1091) | neg: 1091.5563, 1045.5538, 913.5109, 751.4644, 667.4069, 521.3481, 389.3051  pos: 439.3575, 421.3468 |  | + |  | Dammar-24-en-19-al, 3-[O-α-L-rhamno-pyranosyl-(1→2)-O-[β-D-xylopyranosyl-(1→3)]-β-D-xylopyranosyl)oxy]-21-(β-D-glucopyranosyloxy)-20-hydroxy-, (3β) |
| 143 ^n^ | 9.65 | 1145.5353 | -H | C_55_H_86_O_25_ | -2.9 | -2.4 | 345.93  (1145) | neg: 1145.5353, 939.4917, 807.4498, 765.4396, 681.3845, 535.3267, 403.2867 |  | + | + | A-3+Glc+Rha+2Xyl+Mal |
| 144 | 9.69 | 739.4250 | -H | C_39_H_64_O_13_ | -1.9 | -2.6 | 300.48  (785) | neg: 785.4295, 739.4250, 593.3694, 431.3181, 389.2707  pos: 413.3051, 395.2961 |  |  | + | isomer of β-D-glucopyranoside, (1β,3β, 16β,22S)-1-[(6-α-L-rhamnopyranosyl)oxy]-3,22-dihydroxycholesta-5,24-dien-16-yl |
| 145 ^n^ | 9.74 | 913.4814 | -H | C_46_H_74_O_18_ | 1.7 | 1.9 | 341.25  (959) | neg: 959.4788, 913.4814, 767.4596, 681.3848, 535.3271, 403.2853, 375.2881 |  | + | + | B-3+OH−CH_3_+2Rha+Xyl |
| 146 ^n^ | 9.79 | 969.5060 | -H | C_49_H_78_O_19_ | 0.1 | 0.1 | 362.27  (1015) | neg: 1015.5053, 969.5060, 927.4911, 795.4495, 649.3946, 487.3421 | + |  |  | A-3+Glc+Rha+Xyl+Ac |
| 147 ^n^ | 9.92 | 1133.5777 | -H | C_55_H_90_O_24_ | 3.3 | 2.9 | 333.37  (1133) | neg: 1133.5777, 1047.5694, 915.5284, 783.4894, 637.4316, 475.3789 | + |  |  | F-2+Ara+Rha+Xyl+Mal+Glc |
| 148 ^n^ | 9.95 | 1161.5658 | -H | C_56_H_90_O_25_ | -3.5 | -3 | 355.48  (1161) | neg: 1161.5658, 1075.5619, 943.5268, 781.4718, 697.4112, 551.3584, 389.3056  pos: 439.3576, 421.3474 |  | + |  | F-1+2Glc+Rha+Xyl+Mal |
| 149 | 10.03 | 913.5140 | -H | C_47_H_78_O_17_ | -2.1 | -2.3 | 316.44  (959) | neg: 959.5168, 913.5140, 751.4619, 605.4045, 473.3630, 389.2664 | + | + |  | Phanoside |
| 150 | 10.06 | 927.4874 | -H | C_47_H_76_O_18_ | -5.7 | -5.9 | 355.14  (973) | neg: 973.4951, 927.4874, 795.4518, 649.3965, 519.3319, 387.2522  pos: 946.5376, 469.3364, 451.3209, 433.3245 |  |  | + | Dammar-24-en-21-al, 3-[(O-α-L-rhamno-pyranosyl-(1→2)-O-[β-D-glucopyranosyl-(1→3)]-α-L-arabinopyranosyl)oxy]-20,23-dihydroxy-12-oxo-, cyclic 21,23-hemiacetal, (3β,20ξ,21S,23R)- (ACI) |
| 151 | 10.06 | 915.5345 | -H | C_47_H_80_O_17_ | 2.8 | 3.1 | 344.97  (961) | neg: 961.5327, 915.5345, 753.4793, 607.4189, 475.3770, 389.3046 | + |  |  | (3β,6α,12β)-3,12-Dihydroxy-20-(β-D-xylo-pyranosyloxy)dammar-24-en-6-yl 2-O-(-α-L-rhamnopyranosyl)-β-D-glucopyranoside |
| 152 | 10.08 | 867.4741 | -H | C_45_H_72_O_16_ | -0.1 | -0.1 | 306.00  (913) | neg: 913.4743, 867.4741, 735.4332, 589.3748, 457.3327, 345.2780 |  |  | + | Dammar-24-en-19-al, 3-[(O-α-L-arabino-pyranosyl-(1→2)-O-β-D-xylopyranosyl-(1→3)-α-L-arabinopyranosyl)oxy]-21,23-epoxy-20-hydroxy-, (3β,20ξ,23S)- (ACI) |
| 153 ^n^ | 10.10 | 1131.5543 | -H | C_55_H_88_O_24_ | -4.4 | -3.9 | 338.26  (1131) | neg: 1131.5543, 1087.5620, 1045.5555, 913.5119, 751.4625, 605.4059, 473.3629  pos: 1150.5990, 439.3571, 421.3468 | + |  |  | F-1+Glc+Ara+Rha+Xyl+Mal |
| 154 ^n^ | 10.11 | 1145.5354 | +HCOO | C_55_H_86_O_25_ | -2.6 | -2.3 | 342.48  (1145) | neg: 1145.5354, 939.4930, 807.4507, 765.4402, 681.3824, 535.3279, 403.2853  pos: 1164.5941, 453.3365 |  | + | + | A-3+Glc+Rha+2Xyl+Mal |
| 155 ^n^ | 10.20 | 827.4801 | +HCOO | C_42_H_70_O_13_ | 0.8 | 1 | 314.00  (827) | neg: 827.4801, 781.4750, 739.4666, 607.4520, 475.4027, 375.3122 |  |  | + | D-1+2Xyl+COOH |
| 156 ^n^ | 10.21 | 753.4825 | -H | C_41_H_70_O_12_ | 3.6 | 4.8 | 286.43  (799) | neg: 799.4811, 753.4825, 607.4202, 475.3796, 375.2899 |  |  | + | D-2+Ara+Rha |
| 157 ^n^ | 10.22 | 1131.5534 | -H | C_55_H_88_O_24_ | -5.3 | -4.7 | 340.42  (1131) | neg: 1131.5534, 1087.5642, 1045.5579, 913.5118, 751.4613, 605.4055, 473.3577  pos: 1150.5999, 439.3579, 421.3474 |  | + |  | F-1+Glc+Ara+Rha+Xyl+Mal |
| 158 ^n^ | 10.30 | 983.4819 | -H | C_49_H_76_O_20_ | -3.3 | -3.4 | 336.29  (983) | neg: 983.4819, 897.4838, 765.4402, 619.3840, 487.3430 | + |  |  | A-3+Ara+Rha+Xyl+Mal |
| 159 ^n^ | 10.42 | 969.5031 | -H | C_49_H_78_O_19_ | -2.8 | -2.9 | 367.26  (1015) | neg: 1015.5056, 969.5031, 927.4941, 795.4517, 649.3965, 487.3424 | + |  |  | A-3+Glc+Rha+Xyl+Ac |
| 160 | 10.42 | 1091.5955 | -H | C_54_H_92_O_22_ | -4.7 | -4.3 | 358.07  (1137) | neg: 1137.5999, 1091.5955, 929.5441, 783.4879, 621.4376, 459.3844  pos: 1110.6409, 425.3780, 407.3677 | + |  |  | β-D-glucopyranoside, (3β)-21-(β-D-gluco-pyranosyloxy)-20-hydroxydammar-24-en-3-yl O-α-L-rhamnopyranosyl-(1→2)-O-[β-D-glucopyranosyl-(1→3)]- (9CI) |
| 161 ^n^ | 10.54 | 855.4713 | +HCOO | C_43_H_70_O_14_ | -2.9 | -3.4 | 323.21  (855) | neg: 855.4713, 767.4562, 621.401, 489.3597 |  |  | + | A-2+Ara+Rha+Ac |
| 162 ^n^ | 10.63 | 957.4651 | -H | C_47_H_74_O_20_ | -1.3 | -1.4 | 340.45  (957) | neg: 957.4651, 913.4791, 871.4692, 739.4280, 667.4068, 521.3475, 389.3055  pos: 413.3054 |  |  | + | C-1+Glc+Rha+Xyl+Mal−C_3_H_4_ |
| 163 ^n^ | 10.73 | 1131.5538 | -H | C_55_H_88_O_24_ | -4.9 | -4.3 | 342.89  (1131) | neg: 1131.5538, 1087.5608, 1045.5553, 913.5128, 751.4627, 605.4030, 473.3630  pos: 1150.5997, 439.3576, 421.3476 | + |  |  | F-1+Glc+Ara+Rha+Xyl+Mal |
| 164 ^n^ | 10.82 | 753.4807 | +HCOO, -H | C_41_H_70_O_12_ | 1.8 | 2.4 | 287.91  (799) | neg: 799.4814, 753.4807, 607.4194, 475.3800, 375.2892 |  |  | + | D-2+Ara+Rha |
| 165 ^n^ | 10.86 | 1131.5543 | -H | C_55_H_88_O_24_ | -4.4 | -3.9 | 343.88  (1131) | neg: 1131.5543, 10455516, 913.5124, 751.4610, 605.4007, 473.3654 |  | + |  | F-1+Glc+Ara+Rha+Xyl+Mal |
| 166 | 10.86 | 1177.5975 | -H | C_57_H_94_O_25_ | -3.1 | -2.6 | 359.02  (1177) | neg: 1177.5975, 1091.5964, 929.5328, 783.4867, 637.4319, 475.3781 | + |  |  | 6’’-Malonylginsenoside V |
| 167 | 10.98 | 915.5269 | -H | C_47_H_80_O_17_ | -4.8 | -5.2 | 349.55  (961) | neg: 961.5326, 915.5269, 783.4883, 637.4319, 475.3795, 403.2848  pos: 423.3621 | + |  |  | 3β,12β,24S-dihydroxydammar-25-en-20-yl O-β-D-xylopyranosyl-(1→3)-O-α-L-rham-nopyranosyl-(1→6)-β-D-glucopyranoside |
| 168 ^n^ | 10.99 | 827.4801 | +HCOO | C_42_H_70_O_13_ | 0.8 | 1 | 315.25  (827) | neg: 827.4801, 781.4644, 739.4666, 593.3998, 431.3401 |  |  | + | F-1+Glc+Ara |
| 169 | 10.99 | 927.4940 | -H | C_47_H_76_O_18_ | -1.3 | -1.4 | 352.86  (973) | neg: 973.4945, 927.4940, 765.4401, 681.3843, 535.3281, 403.2845, 375.2896 |  | + | + | Gypenoside UL5 |
| 170 ^n^ | 11.03 | 783.4859 | -H | C_42_H_72_O_13_ | -3.6 | -4.6 | 298.79  (829) | neg: 829.4922, 783.4859, 637.4312, 475.3788  pos: 423.3628 | + |  |  | D-1+Glc+Ara |
| 171 ^n^ | 11.13 | 999.4747 | -H | C_49_H_76_O_21_ | -5.4 | -5.4 | 343.98  (999) | neg: 999.4747, 913.4796, 767.4543, 681.3851, 535.3276, 403.2843, 375.2915 |  |  | + | B-3+OH−CH_3_+Ara+2Rha+Mal |
| 172 | 11.18 | 1207.6066 | -H | C_58_H_96_O_26_ | -4.6 | -3.8 | 360.26  (1253) | neg: 1253.6084, 1207.6066, 1075.5706, 913.5174, 751.4642, 605.4056, 473.3635 | + |  |  | Gypenoside UL6 |
| 173 ^n^ | 11.36 | 1015.5073 | -H | C_50_H_80_O_21_ | -4.1 | -4 | 330.86  (1015) | neg: 1015.5073, 971.5182, 929.5082, 783.4570, 489.3580  pos: 1034.5519, 455.3523, 437.3423 | + |  |  | D-3+Glc+Rha+Xyl+Mal |
| 174 ^n^ | 11.46 | 1001.5266 | -H | C_50_H_82_O_20_ | -5.5 | -5.5 | 359.05  (1001) | neg: 1001.5266, 915.5289, 783.4875, 637.4305, 475.3794  pos: 473.3653, 455.3524, 437.3429 | + |  |  | D-1+Glc+Rha+Xyl+Mal |
| 175 ^a^ | 11.58 | 1061.5851 | -H | C_53_H_90_O_21_ | -4.5 | -4.2 | 356.12  (1107) | neg: 1107.5879, 1061.5851, 929.5427, 783.4874, 621.4362, 459.3836, 375.2903  pos: 1080.6312, 425.3784, 407.3677 | + | + |  | GL-7 |
| 176 | 11.61 | 927.4918 | -H | C_47_H_76_O_18_ | -3.5 | -3.8 | 354.34  (973) | neg: 973.4964, 927.4918, 795.4544, 711.3972, 565.3359, 403.2843, 375.2844 | + | + |  | Gypenoside UL5 |
| 177 ^n^ | 11.78 | 1001.5277 | -H | C_50_H_82_O_20_ | -4.4 | -4.4 | 358.15  (1001) | neg: 1001.5277, 915.5282, 783.4885, 637.4333, 475.3798 | + |  |  | D-1+Glc+Rha+Xyl+Mal |
| 178 | 11.92 | 1031.5752 | -H | C_52_H_88_O_20_ | -3.9 | -3.8 | 343.18  (1077) | neg: 1077.5789, 1031.5752, 899.5341, 737.4833, 591.4258, 459.3823, 375.2917  pos: 1050.6210, 425.3782, 407.3677 | + | + |  | β-D-glucopyranoside, (3β)-3-[O-α-L-rham-nopyranosyl-(1→2)-O-[β-D-xylopyranosyl-(1→3)]-α-L-arabinopyranosyl)oxy]-20-hydroxydammar-24-en-21-yl (ACI) |
| 179 ^n^ | 12.12 | 1147.5851 | -H | C_56_H_92_O_24_ | -4.9 | -4.3 | 358.99  (1147) | neg: 1147.5851, 1103.5947, 1061.5841, 929.5428, 783.4867, 621.4354, 535.3269, 403.2846, 375.2903  pos: 1166.6312, 425.3782, 407.3677 | + |  |  | J-6+Glc+Rha+Xyl+Ara |
| 180 ^n^ | 12.15 | 1013.4913 | -H | C_50_H_78_O_21_ | -4.4 | -4.3 | 361.92  (1013) | neg: 1013.4913, 927.4945, 765.4480, 681.3851, 535.3272, 403.2849, 375.2915  pos: 1032.5353, 453.3369, 435.3288 |  | + | + | A-3+Ara+Rha+Glc+Mal |
| 181 ^n^ | 12.22 | 1161.5658 | -H | C_56_H_90_O_25_ | -3.5 | -3 | 351.70  (1161) | neg: 1161.5658, 1075.5665, 929.5265, 783.4754, 681.3854, 535.3278, 403.2859, 375.2894 |  | + |  | B-6+Ara+3Rha+Mal+OH |
| 182 ^n^ | 12.27 | 1073.5497 | +HCOO, -H | C_53_H_86_O_22_ | -3.5 | -3.3 | 362.05  (1119) | neg: 1119.5525, 1073.5497, 941.5090, 779.4564, 761.4471, 615.3907, 453.3374, 403.2840, 359.2956  pos: 1092.5947, 455.3525, 437.3424 | + |  |  | C-1+2Glc+Rha+Xyl |
| 183 ^n^ | 12.29 | 1013.4913 | -H | C_50_H_78_O_21_ | -4.4 | -4.3 | 365.26  (1013) | neg: 1013.4913, 927.4932, 795.4520, 711.3943, 565.3376, 403.2851, 375.2903  pos: 1032.5374, 471.3470, 453.337, 435.3283 | + | + |  | A-3+Glc+Rha+Xyl+Mal |
| 184 | 12.40 | 1075.6006 | -H | C_54_H_92_O_21_ | -4.7 | -4.4 | 355.91  (1121) | neg: 1121.6048, 1075.6006, 929.5435, 767.4664, 683.4008, 537.3438, 375.2745  pos: 455.3525, 437.3424 | + |  |  | Gypenoside VII |
| 185 | 12.42 | 899.4973 | -H | C_46_H_76_O_17_ | -3.1 | -3.4 | 343.97  (945) | neg: 945.5006, 899.4973, 767.4568, 683.3995, 537.3430, 405.3000, 375.2899  pos: 901.5145, 455.3524, 437.3426 | + | + | + | isomer of JGL-7 |
| 186 ^a^ | 12.54 | 897.4819 | -H | C_46_H_74_O_17_ | -2.9 | -3.2 | 343.61  (943) | neg: 943.4840, 897.4819, 765.4410, 681.3842, 535.3271, 403.2846, 375.2892  pos: 453.3365, 435.3260, 417.3153, 407.3317 | + | + | + | GL-21 |
| 187 ^n^ | 12.92 | 1117.5752 | +HCOO | C_55_H_90_O_23_ | -4.3 | -3.8 | 341.01  (1117) | neg: 1117.5752, 1073.5848, 1031.5756, 899.5337, 737.4843, 605.4056, 473.3637, 389.2686 | + | + |  | F-3+Ara+2Xyl+Glc+Mal+CH_3_ |
| 188 ^n^ | 12.95 | 913.5121 | -H | C_47_H_78_O_17_ | -4 | -4.4 | 342.99  (959) | neg: 959.5168, 913.5121, 751.4618, 605.4056, 473.3637, 389.2686  pos: 932.5580, 439.3574, 421.3469 | + | + | + | F-1+Ara+Rha+Glc |
| 189 ^a^ | 13.25 | 895.4681 | -H | C_46_H_72_O_17_ | -1 | -1.1 | 335.05  (941) | neg: 941.4715, 895.4681, 763.4262, 617.3701, 505.3173, 373.2741  pos: 469.3315, 451.3214, 433.3107 | + |  |  | GL-8 |
| 190 | 13.36 | 841.4928 | +HCOO | C_43_H_70_O_13_ | -2.1 | -2.5 | 299.72  (841) | neg: 841.4928, 753.4814, 607.4197, 475.3796, 375.2901 |  |  | + | β-D-glucopyranoside, (3β,12α,20S,24R)-3-(4-O-acetyl-α-L-arabinopyranosyloxy)-20,24-epoxy-25-hydroxydammaran-12-yl 6-deoxy- (ACI) |
| 191 | 13.40 | 897.4822 | -H | C_46_H_74_O_17_ | -2.6 | -2.9 | 344.70  (943) | neg: 943.4852, 897.4822, 765.4409, 681.3833, 535.3278, 403.2846, 375.2900  pos: 916.5264, 471.3479, 453.3366, 435.3271 | + | + | + | isomer of dammar-24-ene-19,21-dial, 3-[O -α-L-rhamnopyranosyl-(1→2)-O-[β-D-xylopyranosyl-(1→3)]-α-L-arabinopyra-nosyl)oxy]-20,23-dihydroxy-, cyclic 21,23-hemiacetal, (3β,20ξ)- (9CI, ACI) |
| 192 ^n^ | 13.47 | 969.5002 | -H | C_49_H_78_O_19_ | -5.7 | -5.9 | 366.74  (1015) | neg: 1015.5052, 969.5002, 927.4931, 795.4504, 711.3940, 565.3400, 403.2851, 375.2910 | + |  |  | A-3+Glc+Rha+Xyl+Ac |
| 193 ^n^ | 13.47 | 1147.5833 | -H | C_56_H_92_O_24_ | -3.9 | -3.4 | 350.61  (1147) | neg: 1147.5833, 1103.5957, 1061.5851, 929.5436, 767.4923, 621.4376, 521.3508, 389.2703  pos: 1166.6335, 439.3574, 421.3468 | + |  |  | J-6+Glc+Rha+Xyl+Ara |
| 194 ^n^ | 13.51 | 913.5145 | -H | C_47_H_78_O_17_ | -1.6 | -1.8 | 348.53  (959) | neg: 959.5167, 913.5145, 781.4716, 635.4175, 473.3640, 389.2703  pos: 439.3585, 421.3478 | + |  |  | F-1+Glc+Rha+Xyl |
| 195 ^n^ | 13.73 | 983.4810 | -H | C_49_H_76_O_20_ | -4.2 | -4.3 | 349.48  (983) | neg: 983.4810, 939.4922, 897.4830, 765.4413, 681.3846, 535.3270, 403.2846, 375.2901  pos: 1002.5281, 453.3367, 435.3266 | + | + | + | B-3+Ara+Rha+Xyl+Mal |
| 196 ^n^ | 13.79 | 985.4949 | -H | C_49_H_78_O_20_ | -4.3 | -4.4 | 353.62  (985) | neg: 985.4949, 941.5087, 899.5181, 767.4581, 683.3998, 537.3431, 405.3006, 375.2900 | + |  |  | B-1+Ara+Rha+Xyl+Mal |
| 197 | 13.83 | 895.4654 | -H | C_46_H_72_O_17_ | -3.7 | -4.1 | 339.48  (941) | neg: 941.4712, 895.4654, 763.4260, 617.3708, 485.3274, 373.2755  pos: 469.3314 | + |  |  | isomer of Dammar-24-en-21-oic acid, 3-[O-α-L-rhamnopyranosyl-(1→2)-O-[β-D-xylopyranosyl-(1→3)]-α-L-arabinopyra-nosyl)oxy]-20,23-dihydroxy-19-oxo-, γ-lactone, (3β,20R,23R)- (ACI) |
| 198 ^n^ | 13.92 | 1147.5836 | -H | C_56_H_92_O_24_ | -4 | -3.5 | 358.33  (1147) | neg: 1147.5836, 1103.5984, 1061.5870, 929.5424, 783.4885, 621.4387, 475.3804  pos: 1166.6317, 459.3829, 441.3734 | + |  |  | F-1+Glc+2Rha+Xyl+Mal |
| 199 | 13.95 | 885.5173 | -H | C_46_H_78_O_16_ | -3.9 | -4.4 | 341.41  (931) | neg: 931.5219, 885.5173, 753.4774, 607.4211, 535.3270, 403.2853  pos: 887.5361, 459.384, 441.3735 |  | + |  | 3β,19,20S,21-tetrahydroxydammar-24-ene 3-O-[α-L-rhamnopyranosyl (1→2)]-[β-D-xylo-pyranosyl (1→3)]-α-L-arabinopyranosyl |
| 200 ^n^ | 13.97 | 911.4986 | -H | C_47_H_76_O_17_ | -1.8 | -2 | 350.15  (957) | neg: 957.5011, 911.4986, 765.4427, 681.3842, 535.3272, 403.2847, 375.2901  pos: 453.337, 435.3258 |  |  | + | B-3+Ara+Rha |
| 201 ^n^ | 14.01 | 751.4254 | -H | C_40_H_64_O_13_ | -1.5 | -2 | 289.81  (797) | neg: 797.4290, 751.4254, 619.3846, 535.3273, 403.2848, 373.2273  pos: 770.4688, 471.3471, 453.3367, 435.3260, 417.3154, 407.3311 | + | + | + | A-3+Ara+Xyl |
| 202 ^n^ | 14.13 | 971.5189 | -H | C_49_H_80_O_19_ | -2.7 | -2.8 | 359.39  (1017) | neg: 1017.5210, 971.5189, 839.4766, 753.4785, 607.4197, 489.3596 | + |  |  | D-1+Ara+Rha+Xyl+Mal |
| 203 | 14.15 | 885.5190 | -H | C_46_H_78_O_16_ | -2.2 | -2.5 | 334.09  (931) | neg: 931.5204, 885.5190, 753.4775, 607.4189, 475.3770, 403.2851, 373.2690 | + |  | + | (3β,20S)-3,19,20,21-tetrahydroxydammar-24-ene 3-O-[α-L-rhamnopyranosyl (1→2)]-[β-D-xylopyranosyl (1→3)]-α-L-arabinopyranosyl |
| 204 ^n^ | 14.22 | 969.5021 | -H | C_49_H_78_O_20_ | -3.8 | -3.9 | 359.75  (1015) | neg: 1015.5074, 969.5021, 927.4954, 765.4431, 681.3851, 535.3275, 403.2841, 375.2898  pos: 988.5488, 453.3371, 435.3268 | + |  |  | A-3+Glc+Rha+Xyl+Ac |
| 205 | 14.28 | 765.4413 | -H | C_41_H_66_O_13_ | -1.2 | -1.6 | 297.53  (811) | neg: 811.4445, 765.4413, 681.3849, 535.3276, 403.2849, 373.2744  pos: 471.3471, 453.3365, 435.3260, 417.3152, 407.3312 | + | + | + | gypenoside UL1 |
| 206 ^n^ | 14.31 | 941.5056 | -H | C_48_H_78_O_18_ | -5.4 | -5.7 | 352.88  (987) | neg: 987.5096, 941.5056, 899.4898, 767.4545, 683.4012, 537.3430, 405.3009, 375.2899 |  | + |  | B-1+OH+Ara+Rha+Xyl+Ac |
| 207 | 14.42 | 1075.5636 | -H | C_53_H_88_O_22_ | -5.3 | -4.9 | 351.63  (1121) | neg: 1121.5668, 1075.5636, 913.5117, 781.4715, 635.4130, 473.3638, 389.3065, 359.2942 | + | + |  | isomer of 3β,20,21-trihydroxydammar-24-ene-19-oxo-3-O-[α-L-rhamnopyranosyl(1 →2)][β-D-xylopyranosyl(1→3)]-β-D-glu-copyranoside-21-O-β-D-glucopyranoside |
| 208 ^a^ | 14.54 | 883.5010 | -H | C_46_H_76_O_16_ | -4.5 | -5.1 | 336.47  (929) | neg: 929.5047, 883.5010, 751.4611, 605.4050, 473.3629, 389.2689  pos: 439.3572, 421.3466 | + | + | + | GL-6 |
| 209 ^n^ | 14.55 | 939.4911 | -H | C_48_H_76_O_18_ | -4.2 | -4.5 | 353.98  (985) | neg: 985.4936, 939.4911, 897.4807, 765.4412, 681.3839, 535.3269, 403.2844, 375.2890  pos: 471.3476, 453.3367, 435.3265 | + | + | + | B-3+Ara+Rha+Xyl+Ac |
| 210 | 14.56 | 943.5226 | -H | C_48_H_80_O_18_ | -4 | -4.2 | 361.12  (989) | neg: 989.5288, 943.5226, 781.4719, 697.4161, 551.3593, 389.3054  pos: 439.3573, 421.3468 | + |  |  | (3β)-3-[(O-α-L-rhamnopyranosyl-(1→2)-O-[β-D-glucopyranosyl-(1→3)]-β-D-glu-copyranosyl)oxy]-20-hydroxydammar-24-en-21-oic acid |
| 211 ^n^ | 14.68 | 1057.5178 | -H | C_52_H_82_O_22_ | -4.1 | -3.9 | 346.60  (1057) | neg: 1057.5178, 1013.5284, 971.5189, 825.4622, 765.4438, 681.3831, 535.3286, 403.2848, 375.2905 | + |  |  | E-1+Ara+2Rha+Mal+Ac |
| 212 ^n^ | 14.81 | 971.5157 | -H | C_49_H_80_O_19_ | -5.9 | -6.1 | 351.47  (971) | neg: 971.5157, 927.5276, 885.5287, 753.4780, 607.4200, 475.3752 |  | + |  | F-2+Ara+Rha+Xyl+Mal |
| 213 | 14.86 | 897.4818 | -H | C_46_H_74_O_17_ | -3 | -3.3 | 334.36  (943) | neg: 943.4846, 897.4818, 765.4423, 681.3829, 535.3279, 403.2859, 375.2967 |  |  | + | isomer of Dammar-24-ene-19,21-dial, 3-[O-α-L-rhamnopyranosyl-(1→2)-O-[β-D-xylopyranosyl-(1→3)]-α-L-arabinopy-ranosyl)oxy]-20,23-dihydroxy-, cyclic 21,23-hemiacetal, (3β,20ξ)- (9CI, ACI) |
| 214 ^n^ | 14.87 | 1029.5200 | -H | C_51_H_82_O_21_ | -7 | -6.8 | 369.54  (1029) | neg: 1029.5200, 883.5020, 781.4728, 697.4132, 551.3591, 389.3063  pos: 1048.5629 | + | + |  | O-3+OH+Glc+2Rha+Mal |
| 215 ^n^ | 14.90 | 981.4653 | -H | C_49_H_74_O_20_ | -4.2 | -4.3 | 339.35  (981) | neg: 981.4653, 937.4768, 895.4656, 763.4260, 617.3688, 485.3275, 373.2741  pos: 1000.5124, 469.3318, 451.3221 | + |  |  | F-3+2Glc+Rha+Xyl+Ac |
| 216 | 14.93 | 1103.5962 | -H | C_55_H_92_O_22_ | -4 | -3.6 | 368.68  (1149) | neg: 1149.5989, 1103.5962, 1061.5845, 929.5428, 783.4860, 621.4368, 459.3834, 375.2904  pos: 1122.6406, 425.378, 407.3674 | + |  |  | β-D-glucopyranoside, (3β)-21-(β-D-glucopyranosyloxy)-20-hydroxydammar-24-en-3-yl O-α-L-rhamnopyranosyl-(1→2) -O-[β-D-xylopyranosyl-(1→3)]-, 6-acetate |
| 217 ^n^ | 15.16 | 941.5126 | -H | C_48_H_78_O_18_ | 1.6 | 1.7 | 354.33  (987) | neg: 987.5099, 941.5126, 899.4932, 767.4520, 683.3994, 537.3409, 405.3006, 375.2895 |  | + |  | B-1+OH+Ara+Rha+Xyl+Ac |
| 218 | 15.17 | 929.5058 | +HCOO | C_46_H_76_O_16_ | -5.2 | -5.6 | 335.56  (929) | neg: 929.5058, 883.4992, 751.4615, 605.4078, 473.3628, 389.2690 |  |  | + | Isomer of Gylongiposide I |
| 219 ^n^ | 15.24 | 939.4932 | -H | C_48_H_76_O_18_ | -2.1 | -2.2 | 354.34  (985) | neg: 985.4960, 939.4932, 897.4808, 765.4409, 681.3845, 535.3270, 403.2844, 375.2892  pos: 471.3462, 453.3263, 435.3263 | + | + | + | B-3+Ara+Rha+Xyl+Ac |
| 220 | 15.28 | 957.5396 | -H | C_49_H_82_O_18_ | -2.7 | -2.8 | 363.56  (1003) | neg: 1003.5436, 957.5396, 915.5302, 783.4874, 637.4318, 475.3788  pos: 976.5847, 423.3626 | + |  |  | β-D-glucopyranoside, (3β,5α,17β)-17-[1,2-dihydroxy-3-(1-hydroxy-1-methylethyl) cyclopentyl]-4,4,8,14-tetramethyl-18-norandrostan-3-yl O-6-α-L-rhamnopyranosyl-(1→2)-O-[β-D-xylopyranosyl-(1→3)]-, 6-acetate (9CI) |
| 221 ^n^ | 15.28 | 1115.5618 | -H | C_55_H_88_O_23_ | -2 | -1.8 | 379.24  (1161) | neg: 1161.5626, 1115.5618, 983.5165, 821.4658, 803.4594, 761.4478, 615.3897, 453.3354  pos: 1134.6061, 455.3526, 437.3426 | + |  |  | C-1+2Glc+Rha+Xyl+Ac |
| 222 ^n^ | 15.36 | 1055.5023 | -H | C_52_H_80_O_22_ | -4 | -3.8 | 371.42  (1055) | neg: 1055.5023, 909.4827, 763.4255, 681.3839, 535.3271, 403.2840, 373.2739 | + |  |  | C-2+Xyl+3Rha-CH_4_+O |
| 223 ^n^ | 15.41 | 1189.5964 | -H | C_58_H_94_O_25_ | -4.2 | -3.5 | 373.79  (1189) | neg: 1189.5964, 1103.5964, 1061.5861, 929.5422, 783.4866, 621.4381, 459.3832  pos: 1208.6425, 425.3783, 407.368 | + |  |  | F-3+2Glc+Rha+Xyl+Ac+Mal |
| 224 ^n^ | 15.55 | 939.4928 | -H | C_48_H_76_O_18_ | -2.5 | -2.7 | 346.14  (985) | neg: 985.4968, 939.4928, 897.4848, 765.4415, 681.3845, 535.3265, 403.2846, 375.2908  pos:958.5377, 471.3477, 453.337, 435.3267 | + | + | + | B-3+Ara+Rha+Xyl+Ac |
| 225 ^n^ | 15.56 | 969.5012 | -H | C_49_H_78_O_19_ | -4.7 | -4.8 | 341.03  (969) | neg: 969.5012, 925.5139, 883.5033, 751.4625, 605.4055, 473.3633, 389.2687  pos: 988.547, 457.3681, 439.3573, 421.347 | + | + | + | F-1+Ara+Rha+Xyl+Mal |
| 226 | 15.58 | 883.5024 | -H | C_46_H_76_O_16_ | -3.1 | -3.5 | 341.52  (929) | neg: 929.5059, 883.5024, 751.4601, 605.4047, 473.3616, 389.2858  pos: 885.5204, 439.3584, 421.3487 | + |  | + | Gypenoside UL3 |
| 227 ^n^ | 15.67 | 1131.5597 | -H | C_55_H_88_O_24_ | 1 | 0.9 | 335.80  (1131) | neg: 1131.5597, 913.5045, 751.4612, 605.4075, 473.3626  pos: 1150.6019, 439.3574 |  | + |  | F-1+Glc+Ara+Rha+Xyl+Mal |
| 228 | 15.82 | 881.4875 | -H | C_46_H_74_O_16_ | -2.4 | -2.7 | 340.12  (927) | neg: 927.4896, 881.4875, 749.4465, 603.3896, 471.3475, 389.3043, 359.2591  pos: 900.5308, 455.3519, 437.3416, 419.3308 | + | + | + | isomer of GL-12 |
| 229 ^n^ | 15.82 | 985.4966 | +HCOO | C_48_H_76_O_18_ | -4.2 | -4.3 | 355.35  (985) | neg: 985.4966, 897.4832, 765.4402, 681.3831, 535.3270, 403.2843  pos: 958.5376, 453.3373, 435.3275 | + |  |  | B-3+Ara+Rha+Xyl+Ac |
| 230 | 15.86 | 807.4509 | -H | C_43_H_68_O_14_ | -2.2 | -2.7 | 326.02  (953) | neg: 853.4545, 807.4509, 765.4407, 681.3837, 535.3268, 403.2851, 373.2742  pos: 471.3478, 453.3362, 435.326, 417.3156, 407.3339 | + | + | + | Dammar-24-en-21-oic acid, 3-[(6-O-acetyl-3-O-β-D-xylopyranosyl-β-D-glucopyra-nosyl)oxy]-20,23-dihydroxy-, γ-lactone, (3β,23R)- (ACI) |
| 231 ^n^ | 15.88 | 1013.5278 | -H | C_51_H_82_O_20_ | -4.3 | -4.2 | 362.05  (1059) | neg: 1059.5320, 1013.5278, 971.5185, 839.4775, 765.4423, 681.3860, 535.3271, 403.2847  pos: 453.3368, 435.3253 | + |  |  | A-2+Ara+Rha+Xyl+C_5_H_6_O_3_ |
| 232 ^n^ | 15.91 | 913.5129 | -H | C_47_H_78_O_17_ | -3.2 | -3.5 | 356.67  (959) | neg: 959.5166, 913.5129, 781.5142, 697.4164, 551.3589, 389.3051  pos: 932.5575, 439.3572, 421.3467 | + | + | + | F-1+Glc+Rha+Xyl |
| 233 ^n^ | 16.03 | 781.4718 | -H | C_42_H_70_O_13_ | -2 | -2.6 | 313.96  (827) | neg: 827.4769, 781.4718, 697.4142, 551.3571, 389.3048  pos: 439.3577, 421.3472 | + |  |  | B-1+ Glc+Rha |
| 234 | 16.05 | 737.4478 | -H | C_40_H_66_O_12_ | 0.2 | 0.3 | 285.77  (783) | neg: 783.4510, 737.4478, 605.4073, 473.3617, 389.3050  pos: 756.4904, 457.3695, 439.3577, 421.3472 | + |  |  | Dammar-24-en-19-al, 20,21-dihydroxy-3-[(3-O-β-D-xylopyranosyl-α-L-arabinopyranosyl)oxy]-, (3β)- (ACI) |
| 235 | 16.18 | 881.4858 | -H | C_46_H_74_O_16_ | -4.1 | -4.7 | 341.46  (927) | neg: 927.4899, 881.4858, 749.4461, 603.3905, 471.3489, 443.3522, 389.2674, 359.25752  pos: 900.5319, 455.3521, 437.3417, 419.3310, 409.3463 | + | + | + | isomer of GL-13 |
| 236 ^n^ | 16.21 | 999.5107 | -H | C_50_H_80_O_20_ | -5.8 | -5.8 | 361.89  (999) | neg: 999.5107, 955.5218, 913.5129, 781.4726, 697.4149, 551.3587, 389.3055  pos: 1018.5579, 439.3576, 421.3469 | + | + | + | F-1+Glc+Rha+Xyl+Mal |
| 237 | 16.34 | 751.4619 | -H | C_41_H_68_O_12_ | -1.4 | -1.9 | 299.47  (797) | neg: 797.4654, 751.4619, 605.4056, 473.3634, 389.2691  pos: 775.4587, 439.3571, 421.3463, 313.2527, 297.2598 | + |  | + | Gypenoside D |
| 238 ^n^ | 16.48 | 925.5128 | -H | C_48_H_78_O_17_ | -3.3 | -3.6 | 338.5  (971) | neg: 971.5156, 925.5128, 883.5020, 751.4636, 605.4067, 473.3642, 389.3049  pos: 457.368, 439.3573, 421.3466 | + |  | + | B-1+Ara+Rha+Xyl+Ac |
| 239 | 16.56 | 883.5025 | -H | C_46_H_76_O_16_ | -3 | -3.4 | 346.88  (929) | neg: 929.5059, 883.5025, 751.4619, 667.4058, 521.3485, 389.3059  pos: 439.3574, 421.3466 | + | + | + | Isomer of Gylongiposide I |
| 240 ^n^ | 16.56 | 1013.5280 | -H | C_51_H_82_O_20_ | -4.1 | -4 | 361.15  (1059) | neg: 1059.5315, 1013.5280, 971.5179, 839.4798, 751.4628, 667.4089, 521.3462, 389.3048 | + |  |  | A-1+Ara+Rha+Xyl+C_5_H_6_O_4_ |
| 241 ^n^ | 16.60 | 1029.5228 | -H | C_51_H_82_O_21_ | -4.2 | -4.1 | 344.81  (1029) | neg: 1029.5228, 985.5338, 943.5240, 925.5131, 883.5027, 751.4635, 667.4058, 521.3484, 389.3055  pos: 1048.5677, 455.3521, 439.3571, 421.3468 | + |  |  | G-2-H_2_+Ara+Rha+Xyl+Ac+Mal |
| 242 | 16.72 | 941.5075 | -H | C_48_H_78_O_18_ | -3.5 | -3.7 | 361.01  (987) | neg: 987.5108, 941.5075, 779.4561, 633.4001, 471.3463, 359.2967  pos: 455.352, 437.3415 | + |  |  | isomer of Dammar-24-en-21-oic acid, 3-[O-α-L-rhamnopyranosyl-(1→2)-O-[β-D-glucopyranosyl-(1→3)]-β-D-glucopyran-osyl)oxy]-20,23-dihydroxy-, γ-lactone, (3β,23S)- (9CI) |
| 243 ^n^ | 16.78 | 807.4520 | -H | C_43_H_68_O_14_ | -1.1 | -1.4 | 324.06  (853) | neg: 853.4544, 807.452, 765.4398, 681.3831, 535.3269, 403.2843, 373.2732  pos: 471.3475, 453.3364, 435.3262 | + | + | + | C-1+Glc+Xyl+Ac |
| 244 | 16.84 | 823.4810 | -H | C_44_H_72_O_14_ | -3.4 | -4.1 | 330.45  (869) | neg: 869.4866, 823.4810, 781.4723, 751.4631, 667.4063, 521.3488, 389.3052 | + |  |  | isomer of Damulin D |
| 245 ^n^ | 16.94 | 927.5290 | -H | C_48_H_80_O_17_ | -2.7 | -2.9 | 356.27  (973) | neg: 973.5330, 927.5290, 781.4719, 697.4151, 551.3588, 389.3055  pos: 946.5753, 439.3576, 421.347 | + |  |  | J-15+Glc+2Rha |
| 246 ^n^ | 16.98 | 967.4847 | -H | C_49_H_76_O_19_ | -5.6 | -5.8 | 348.61  (967) | neg: 967.4847, 923.4966, 881.4871, 749.4456, 603.3893, 471.3473, 359.2585  pos: 986.5308, 455.3519, 437.3415, 419.3309 |  | + | + | C-1+Ara+Rha+Xyl+Mal |
| 247 | 17.02 | 913.5136 | -H | C_47_H_78_O_17_ | -2.5 | -2.7 | 334.27  (959) | neg: 959.5177, 913.5136, 751.4626, 605.4055, 473.3634, 389.3048 | + |  |  | isomer of β-D-glucopyranoside, (3β,20ξ)-21,23-epoxy-20,21-dihydroxydammar-24-en-3-yl O-α-L-rhamnopyranosyl-(1→2)-O-[β-D-xylopyranosyl-(1→3)]- (9CI, ACI) |
| 248 ^n^ | 17.03 | 853.4548 | +HCOO | C_43_H_68_O_14_ | -3.8 | -4.5 | 317.69  (853) | neg: 853.4548, 807.4451, 765.4417, 681.3837, 535.3268, 403.2843, 373.2736  pos: 453.3367, 435.3273 |  |  | + | C-1+Glc+Xyl+Ac |
| 249 ^n^ | 17.03 | 939.4907 | -H | C_48_H_76_O_18_ | -4.6 | -4.9 | 358.14  (985) | neg: 985.4957, 939.4907, 897.4826, 765.4421, 681.3840, 535.3273, 403.2849, 375.2896  pos: 958.5364, 471.3476, 453.3362, 435.3259 | + | + | + | B-3+Ara+Rha+Xyl+Ac |
| 250 | 17.04 | 929.5436 | -H | C_48_H_82_O_17_ | -3.8 | -4.1 | 356.24  (975) | neg: 975.5474, 929.5436, 767.4930, 621.4366, 459.3838, 375.2901  pos: 948.5888, 425.3785, 407.3674 | + |  |  | (3β)-20,21-Dihydroxydammar-24-en-3-yl O-α-L-rhamnopyranosyl-(1→2)-O-[β-D-glucopyranosyl-(1→3)]-β-D-glucopyranoside |
| 251 ^n^ | 17.07 | 1015.5434 | -H | C_51_H_84_O_20_ | -4.4 | -4.3 | 363.04  (1015) | neg: 1015.5434, 929.5446, 767.4929, 621.4372, 459.3841, 375.2901 | + |  |  | F-3+2Glc+Rha+Mal |
| 252 ^n^ | 17.15 | 735.4321 | -H | C_40_H_64_O_12_ | 0.1 | 0.1 | 292.28  (781) | neg: 781.4349, 735.4321, 603.3912, 471.3479  pos: 754.4755, 455.3528, 437.3421, 419.3315 |  | + | + | C-1+Ara+Xyl |
| 253 ^n^ | 17.19 | 997.4995 | -H | C_50_H_78_O_20_ | -1.3 | -1.3 | 370.11  (1043) | neg: 1043.5007, 997.4995, 897.4814, 765.4408, 681.3852, 535.3276, 403.2854, 375.2895  pos: 1016.5413, 453.3365, 435.326, 417.3155 | + | + | + | E-1+Ara+Rha+Xyl+C_4_H_2_O_2_ |
| 254 ^n^ | 17.27 | 749.4454 | -H | C_41_H_66_O_12_ | -2.2 | -2.9 | 310.23  (795) | neg: 795.4500, 749.4454, 603.3893, 471.3492  pos: 773.4437, 455.3520, 437.3417, 419.3310 |  | + | + | C-1+Ara+Rha |
| 255 ^n^ | 17.27 | 1029.5225 | -H | C_50_H_80_O_19_ | -4.5 | -4.4 | 367.12  (1029) | neg: 1029.5225, 985.5355, 943.5225, 811.4832, 665.4280, 503.3744, 473.3643, 389.3058  pos: 455.3526, 437.3566 | + |  |  | D-1+Glc+Rha+Xyl+Mal+CHO |
| 256 ^n^ | 17.36 | 967.4850 | -H | C_49_H_76_O_19_ | -5.3 | -5.5 | 351.91  (967) | neg: 967.4850, 923.4985, 881.4864, 749.4466, 603.3887, 471.3487, 389.3045, 359.2582  pos: 986.5322, 455.3527, 437.3419, 419.3318 |  |  | + | C-1+Ara+Rha+Xyl+Mal |
| 257 | 17.44 | 899.5360 | -H | C_47_H_80_O_16_ | -0.8 | -0.9 | 343.21  (945) | neg: 945.5366, 899.5360, 737.4820, 591.4258, 459.3866, 375.2905 | + |  |  | Isomer of β-D-Glucopyranoside, (3β)-20,21-dihydroxydammar-24-en-3-yl O-α-L-rhamnopyranosyl-(1→2)-O-[β-D-xylopyranosyl-(1→3)]- (ACI) |
| 258 ^n^ | 17.44 | 969.5002 | -H | C_49_H_78_O_19_ | -5.7 | -5.9 | 354.93  (969) | neg: 969.5002, 925.5121, 883.5029, 751.4611, 667.4062, 521.3483, 389.3056  pos: 439.3573, 421.3466 | + | + | + | F-1+Ara+Rha+Xyl+Mal |
| 259 ^n^ | 17.50 | 1027.5074 | -H | C_51_H_80_O_21_ | -4 | -3.9 | 369.03  (1027) | neg: 1027.5074, 941.5100, 779.4578, 633.4014, 521.3477, 389.3065, 359.2949 | + |  |  | B-3+CH_3_+Ara+Rha+Glc+Mal |
| 260 a | 17.51 | 941.5081 | -H | C_48_H_78_O_18_ | -2.9 | -3.1 | 359.08  (987) | neg: 987.5113, 941.5081, 779.4562, 633.3986, 521.3470, 359.2941  pos: 960.5522, 455.3519, 437.3419 | + |  | + | GJ-17 |
| 261 | 17.53 | 929.5061 | -H | C_47_H_78_O_18_ | -4.9 | -5.3 | 346.57  (975) | neg: 975.5101, 929.5061, 797.4661, 489.3600 | + | + | + | Gypenoside VN7 |
| 262 ^n^ | 17.63 | 749.4451 | -H | C_41_H_66_O_12_ | -2.5 | -3.3 | 309.91  (795) | neg: 795.4509, 749.4451, 603.3890, 471.3445, 359.2564  pos: 773.4495, 455.3523, 437.3421, 419.332 |  | + | + | C-1+Ara+Rha |
| 263 ^n^ | 17.66 | 999.5110 | -H | C_50_H_80_O_20_ | -5.5 | -5.5 | 363.82  (999) | neg: 999.5110, 955.5239, 913.5137, 751.4632, 667.4052, 521.3483, 389.3052 | + | + |  | F-1+Glc+Rha+Ara+Mal |
| 264 ^n^ | 17.69 | 923.4979 | -H | C_48_H_76_O_17_ | -2.5 | -2.7 | 350.18  (969) | neg: 969.4984, 923.4979, 881.4890, 749.4470, 603.3906, 471.3489, 359.2580  pos: 455.3523, 437.3408, 419.3321 |  | + | + | C-1+Ara+Rha+Xyl+Ac |
| 265 | 17.74 | 913.5110 | -H | C_47_H_78_O_17_ | -5.1 | -5.6 | 346.00  (959) | neg: 959.5162, 913.5110, 781.4720, 635.4160, 473.3642, 389.3048  pos: 915.5337, 455.3529, 437.3423, 419.3293 | + | + |  | β-D-glucopyranoside, (3β,20ξ)-21,23-epoxy-20,21-dihydroxydammar-24-en-3-yl O-α-L-rhamnopyranosyl-(1→2)-O-[β-D-xylopyranosyl-(1→3)]- (9CI, ACI) |
| 266 ^n^ | 17.76 | 793.4716 | -H | C_43_H_70_O_13_ | -2.2 | -2.8 | 322.84  (839) | neg: 839.4751, 793.4716, 751.4610, 605.4054, 473.3655, 389.2697  pos:439.3575, 421.3467 | + | + | + | F-1+Xyl+Rha+CH3CO |
| 267 ^n^ | 17.76 | 923.4950 | -H | C_48_H_76_O_17_ | -5.4 | -5.8 | 350.69  (969) | neg: 969.4995, 923.4950, 881.4883, 749.4467, 603.3894, 471.3472, 389.3058  pos: 455.3583, 437.3472, 419.3378 |  |  | + | N-2+Ara+Rha+Xyl+Ac |
| 268 ^a^ | 17.83 | 911.4978 | -H | C_47_H_76_O_17_ | -2.6 | -2.9 | 349.48  (957) | neg: 957.5021, 911.4978, 779.4568, 633.4002, 471.3476, 359.2944  pos: 930.5430, 455.3524, 437.3419, 419.3315 | + |  |  | GL-3 |
| 269 ^n^ | 18.03 | 997.4973 | -H | C_50_H_78_O_20_ | -3.5 | -3.5 | 357.02  (997) | neg: 997.4973, 953.5067, 911.4991, 779.4581, 633.3996, 471.3461, 359.2948 | + |  |  | C-1+ Glc+Rha+Ara+Mal |
| 270 ^n^ | 18.07 | 923.4984 | -H | C_48_H_76_O_17_ | -2 | -2.5 | 350.81  (969) | neg: 969.4989, 923.4984, 881.4870, 749.4490, 603.3903, 471.3467, 359.2579 |  |  | + | C-1+Ara+Rha+Xyl+Ac |
| 271 ^a^ | 18.19 | 899.5344 | -H | C_47_H_80_O_16_ | -2.4 | -2.7 | 351.09  (945) | neg: 945.5371, 899.5344, 767.4939, 621.4386, 459.3829, 375.2930 | + | + | + | GL-18 |
| 272 | 18.28 | 955.5248 | -H | C_49_H_80_O_18_ | -1.8 | -1.9 | 366.89  (1001) | neg: 1001.5278, 955.5248, 913.5140, 781.4727, 697.4162, 551.3591, 389.3058 | + |  |  | (3β)-3-[(O-α-L-rhamnopyranosyl-(1→2)-O-[β-D-xylopyranosyl-(1→3)]-6-O-acetyl-β-D-glucopyranosyl)oxy]-20,21-dihydroxy dammar-24-en-19-al |
| 273 | 18.29 | 867.4716 | -H | C_45_H_72_O_16_ | -2.6 | -3 | 331.16  (913) | neg: 913.4739, 867.4716, 735.4323, 589.3740 |  |  | + | Dammar-24-en-19-al, 3-[(O-α-L-arabino-pyranosyl-(1→2)-O-β-D-xylopyranosyl-(1→3)-α-L-arabinopyranosyl)oxy]-21,23-epoxy-20-hydroxy-, (3β,20ξ,23S)- (ACI) |
| 274 ^n^ | 18.35 | 985.5330 | -H | C_50_H_82_O_19_ | -4.2 | -4.3 | 357.60  (985) | neg: 985.5330, 941.5459, 899.5359, 767.4931, 621.4377, 459.3857, 389.3056, 359.2954 | + |  |  | F-3+Glc+Rha+Ara+Mal |
| 275 | 18.36 | 925.5148 | -H | C_48_H_78_O_17_ | -1.3 | -1.4 | 357.15  (971) | neg: 971.5170, 925.5148, 779.4558, 667.4072, 521.3492, 389.3059, 359.2953 | + |  |  | isomer of Longipenoside A |
| 276 ^n^ | 18.36 | 767.4938 | -H | C_42_H_72_O_12_ | -1.6 | -2.1 | 310.33  (813) | neg: 813.4986, 767.4938, 667.4061, 521.3480, 389.3055, 359.2954  pos: 455.3522, 437.3418 | + |  |  | F-3+Ara+Rha+CH3 |
| 277 ^n^ | 18.37 | 923.4961 | -H | C_48_H_76_O_17_ | -4.3 | -4.7 | 342.77  (969) | neg: 969.4997, 923.4961, 881.4875, 749.4463, 603.3909, 471.3480, 359.2575  pos: 455.3528, 437.3424, 419.3286 |  | + | + | C-1+Ara+Rha+Xyl+Ac |
| 278 | 18.39 | 911.4986 | -H | C_47_H_76_O_17_ | -1.8 | -2 | 351.36  (957) | neg: 957.5027, 911.4986, 779.4576, 633.4008, 521.3484, 359.2950  pos: 930.5436, 455.3523, 437.3418 | + |  |  | isomer of GL-3 |
| 279 | 18.40 | 955.5251 | -H | C_49_H_80_O_18_ | -1.5 | -1.6 | 367.37  (1001) | neg: 1001.5259, 955.5251, 913.5128, 781.4719, 697.4166, 551.3599, 389.3061 |  | + |  | β-D-glucopyranoside, (3β,20ξ)-21,23-epoxy-20,21-dihydroxydammar-24-en-3-yl O-α-L-rhamnopyranosyl-(1→2)-O-[β-D-xylopyranosyl-(1→3)]-, 6-acetate |
| 280 ^a^ | 18.44 | 881.4877 | -H | C_46_H_74_O_16_ | -2.2 | -2.5 | 340.68  (927) | neg: 927.4924, 881.4877, 749.4455, 603.3904, 471.3468, 389.3043, 359.2592  pos: 900.5425, 455.3524, 437.3425 | + | + |  | GL-12 |
| 281 n | 18.45 | 969.5005 | -H | C_49_H_78_O_19_ | -5.4 | -5.6 | 337.33  (969) | neg: 969.5005, 925.5123, 883.5003, 751.4620, 667.4046, 521.3483, 389.3052  pos: 988.5483, 439.3578, 421.3472 |  | + | + | F-1+Ara+Rha+Xyl+Mal |
| 282 ^n^ | 18.50 | 897.5173 | -H | C_47_H_78_O_16_ | -3.9 | -4.3 | 353.03  (943) | neg: 943.5220, 897.5173, 765.4723, 633.4000, 521.3479, 359.2944  pos: 455.3521, 437.3417 | + | + |  | F-3+Glc+2Xyl+CH_2_ |
| 283 | 18.56 | 925.5125 | -H | C_48_H_78_O_17_ | -3.6 | -3.9 | 354.23  (971) | neg: 971.5170, 925.5125, 779.4573, 633.3996, 471.3471, 389.3055, 359.2947 | + | + | + | Longipenoside A |
| 284 ^a^ | 18.63 | 911.4954 | -H | C_47_H_76_O_17_ | -5 | -5.5 | 350.08  (957) | neg: 957.5002, 911.4954, 779.4562, 633.3993, 501.3830, 471.3489 | + | + | + | GL-4 |
| 285 ^n^ | 18.69 | 793.4716 | -H | C_43_H_70_O_13_ | -2.2 | -2.8 | 318.93  (839) | neg: 839.4754, 793.4716, 751.4609, 605.4056, 473.3624, ,389.2690  pos: 439.3575, 421.3473 |  |  | + | F-1+Ara+Rha+CH_3_CO |
| 286 ^a^ | 18.70 | 881.4884 | -H | C_46_H_74_O_16_ | -1.5 | -1.7 | 343.88  (927) | neg: 927.4895, 881.4884, 749.4476, 603.3909, 491.3360, 359.2951  pos: 900.5332, 455.3524, 437.344 | + |  | + | GL-13 |
| 287 ^n^ | 18.72 | 971.5167 | +HCOO | C_48_H_78_O_17_ | -4.9 | -5 | 350.07  (971) | neg: 971.5167, 925.5104, 897.4815, 751.4628, 605.4081, 473.3644, 389.3035 |  | + | + | B-1+Ara+2Rha+CHO |
| 288 ^n^ | 18.73 | 913.5192 | -H | C_47_H_78_O_17_ | 3.1 | 3.4 | 351.97  (959) | neg: 959.5152, 913.5192, 781.4720, 635.4156, 491.3360, 359.2951 |  |  | + | B-1+Ara+Rha+Xyl+CH_2_O |
| 289 ^n^ | 18.85 | 983.5149 | -H | C_50_H_80_O_19_ | -6.7 | -6.8 | 364.02  (1029) | neg: 1029.5198, 983.5149, 883.5014, 751.4619, 605.4067, 473.3638, 389.3029  pos: 1002.5617, 457.3677, 439.3575, 421.3486 |  |  | + | B-1+Ara+Rha+Xyl+C_4_H_4_O_3_ |
| 290 ^n^ | 18.89 | 839.4749 | +HCOO | C_43_H_70_O_13_ | -4.4 | -5.2 | 319.21  (839) | neg: 839.4749, 751.4597, 605.4041, 473.3644, 389.2702 |  |  | + | F-1+Ara+Rha+CH_3_CO |
| 291 ^n^ | 18.90 | 999.5106 | -H | C_50_H_80_O_20_ | -5.9 | -5.9 | 358.58  (999) | neg: 999.5106, 955.5230, 913.5164, 781.4711, 635.4160, 473.3624 | + |  |  | F-1+Glc+Rha+Xyl+Mal |
| 292 | 18.92 | 869.5244 | -H | C_46_H_78_O_15_ | -1.8 | -2.1 | 337.66  (915) | neg: 915.5280, 869.5244, 737.4836, 591.4265, 459.3841, 375.2900  pos: 888.5688, 425.3780, 407.3673 | + | + | + | α-L-Arabinopyranoside, (3β)-20,21-dihydroxydammar-24-en-3-yl O-α-L-rhamnopyranosyl-(1→2)-O-[β-D-xylopyranosyl-(1→3)]- (ACI) |
| 293 | 18.95 | 837.4599 | +HCOO | C_43_H_68_O_13_ | -3.7 | -4.4 | 328.50  (837) | neg: 837.4599, 749.4458, 603.3884, 441.3370, 359.2574  pos: 455.3522, 437.3418, 419.3317 |  |  | + | New(Lanost-7-en-18-oic acid, 16-(acetyloxy)-3-[[4-O-(6-deoxy-β-D-glucopyranosyl)-β-D-xylopyranosyl]oxy]-20-hydroxy-, γ-lactone, (3β,9β,16α)- (9CI)) |
| 294 ^n^ | 18.95 | 897.5191 | -H | C_47_H_78_O_16_ | -2.1 | -2.3 | 349.44  (943) | neg: 943.5215, 897.5191, 765.4775, 619.4214, 473.3629, 359.2943 | + |  |  | F-3+Glc+2Xyl+CH_2_ |
| 295 | 18.97 | 925.5139 | -H | C_48_H_78_O_17_ | -2.2 | -2.4 | 355.94  (971) | neg: 971.5175, 925.5139, 779.4564, 633.4013, 521.3486, 389.3056, 359.2947 | + |  |  | isomer of Longipenoside A |
| 296 | 19.04 | 913.5500 | -H | C_48_H_82_O_16_ | -2.5 | -2.7 | 351.91  (959) | neg: 959.5547, 913.5500, 767.4929, 621.4377, 459.3847, 375.2882  pos: 425.3792, 407.3681 | + |  |  | Gynosaponin II |
| 297 ^n^ | 19.14 | 925.5109 | -H | C_48_H_78_O_17_ | -5.2 | -5.6 | 357.63  (971) | neg: 971.5162, 925.5109, 883.5031, 751.4633, 667.4054, 521.3476, 389.3054  pos: 439.3577, 421.3506 |  | + | + | B-1+Ara+Rha+Xyl+Ac |
| 298 ^n^ | 19.17 | 967.4863 | -H | C_49_H_76_O_19_ | -4 | -4.1 | 348.87  (967) | neg: 967.4863, 923.4988, 881.4886, 749.4476, 603.3901, 491.3379, 359.3140  pos: 986.5316, 455.3523, 437.3419 | + |  |  | C-1+Ara+Rha+Xyl+Mal |
| 299 | 19.20 | 943.5231 | -H | C_48_H_80_O_18_ | -3.5 | -3.7 | 356.41  (989) | neg: 989.5528, 943.5231, 781.5082, 635.4529 | + |  |  | isomer of (3β)-3-[(O-α-L-rhamnopy-ranosyl-(1→2)-O-[β-D-glucopyranosyl-(1→3)]-β-D-glucopyranosyl)oxy]-20-hydroxydammar-24-en-21-oic acid |
| 300 ^n^ | 19.24 | 913.5496 | -H | C_48_H_82_O_16_ | -2.9 | -3.2 | 356.88  (959) | neg: 959.5527, 913.5496, 781.5082, 635.4529, 503.3750, 389.3062 | + |  |  | F-2+Ara+Rha+Xyl+C2H6 |
| 301 | 19.27 | 955.5223 | -H | C_49_H_80_O_18_ | -4.3 | -4.5 | 365.29  (1001) | neg: 1001.5278, 955.5223, 913.5151, 781.4718, 697.4156, 551.3580, 389.3054  pos: 974.5676, 439.3572, 421.3466 | + | + |  | (3β)-3-[(O-α-L-rhamnopyranosyl-(1→2)-O-[β-D-xylopyranosyl-(1→3)]-6-O-acetyl-β-D-glucopyranosyl)oxy]-20,21-dihydroxydammar-24-en-19-al |
| 302 ^n^ | 19.29 | 751.4606 | -H | C_41_H_68_O_12_ | -2.7 | -3.6 | 298.34  (797) | neg: 797.4658, 751.4606, 667.4070, 521.3484, 389.3058  pos: 439.3577, 421.3469, 313.2548, 297.2604 |  | + | + | B-1+Ara+Rha |
| 303 | 19.44 | 911.4961 | -H | C_47_H_76_O_17_ | -4.3 | -4.7 | 350.34  (957) | neg: 957.9980, 911.4961, 779.4557, 633.3996, 501.3581, 389.3245  pos: 453.3361, 435.3263, 417.3168, 407.3323 |  | + | + | isomer of Dammar-24-ene-19,21-dial, 3-[(O-α-L-rhamnopyranosyl-(1→2)-O-[β-D-xylopyranosyl-(1→3)]-α-L-arabinopy-ranosyl)oxy]-20,23-dihydroxy-, cyclic 21,23-(methyl acetal), (3β,20ξ,23S)- (ACI) |
| 304 | 19.49 | 913.5148 | -H | C_47_H_78_O_17_ | -1.3 | -1.4 | 349.49  (959) | neg: 959.5161, 913.5148, 781.4716, 635.4153, 473.36345, 389.3069  pos: 915.5314, 455.3500, 437.3413 | + |  |  | isomer of β-D-Glucopyranoside, (3β,20ξ)-21,23-epoxy-20,21-dihydroxydammar-24-en-3-yl O-α-L-rhamnopyranosyl-(1→2)-O-[β-D-xylopyranosyl-(1→3)]- (9CI, ACI) |
| 305 ^n^ | 19.55 | 997.4974 | -H | C_50_H_78_O_20_ | -3.4 | -3.4 | 358.96  (997) | neg: 997.4974, 953.5070, 911.4968, 779.4567, 633.4003, 501.3589, 473.3892, 389.3047, 375.2899  pos: 1016.541, 453.3368, 435.3261 | + | + | + | B-3+CH_3_+Ara+Rha+Xyl+Mal |
| 306 ^n^ | 19.57 | 1041.5228 | -H | C_52_H_82_O_21_ | -4.2 | -4 | 312.92  (1041) | neg: 1041.5228, 997.5834, 955.5756, 823.5270, 781.5142, 697.4529, 551.3877, 389.3245  pos: 455.3661, 437.355, 419.1177 | + |  |  | B-1+Glc+Rha+Xyl+Mal+Ac |
| 307 ^n^ | 19.63 | 867.5074 | -H | C_46_H_76_O_15_ | -3.2 | -3.7 | 341.35  (913) | neg: 913.5115, 867.5074, 735.4668, 589.4112, 457.3669, 375.2902  pos: 423.3627, 405.3523 | + | + | + | N-3+Ara+Rha+Xyl |
| 308 ^n^ | 19.65 | 985.5339 | -H | C_50_H_82_O_19_ | -3.3 | -3.3 | 370.80  (1031) | neg: 1031.5385, 985.5339, 853.4936, 781.4725, 503.3735  pos: 1004.5793, 455.3523, 437.3415, 419.329 | + |  |  | B-1+Ara+Rha+Xyl+C_4_H_6_O_3_ |
| 309 | 19.75 | 791.4593 | -H | C_43_H_68_O_13_ | 1.1 | 1.4 | 325.25  (837) | neg: 837.4598, 791.4593, 749.4459, 603.3898, 441.3396, 359.2604  pos: 455.3526, 437.3422, 419.3320 |  |  | + | Lanost-7-en-18-oic acid, 16-(acetyloxy)-3-[[4-O-(6-deoxy-β-D-glucopyranosyl)-β-D-xylopyranosyl]oxy]-20-hydroxy-, γ-lactone, (3β,9β,16α)- (9CI)) |
| 310 ^n^ | 19.85 | 849.4668 | -H | C_45_H_70_O_15_ | 3.2 | 3.8 | 323.73  (895) | neg: 895.4647, 849.4668, 717.4222, 571.3659, 439.3213  pos: 441.3368, 423.3261 |  |  | + | P-1+Ara+Rha+Xyl−H_2_ |
| 311 ^n^ | 19.88 | 1071.5322 | -H | C_53_H_84_O_22_ | -5.4 | -5 | 365.66  (1071) | neg: 1071.5322, 1027.5436, 985.5343, 853.4943, 779.4576, 633.3997, 567.3688, 503.3735  pos: 1090.5804, 455.3524, 437.3425 | + |  |  | B-1+OH+Ara+Rha+Xyl+CH_3_+Mal+C_3_H_4_O_2_ |
| 312 | 19.89 | 1001.5284 | +HCOO | C_49_H_80_O_18_ | -3.7 | -3.7 | 365.69  (1001) | neg: 1001.5284, 955.5169, 913.5134, 751.4636, 667.4053, 521.3475, 389.3047  pos: 974.5717, 439.3582, 421.3486 | + |  |  | (3β)-3-[(O-α-L-rhamnopyranosyl-(1→2)-O-[β-D-xylopyranosyl-(1→3)]-6-O-acetyl-β-D-glucopyranosyl)oxy]-20,21-dihydroxy dammar-24-en-19-al |
| 313 | 19.94 | 837.4598 | +HCOO | C_43_H_68_O_13_ | -3.8 | -4.5 | 324.76  (837) | neg: 837.4598, 749.446, 603.3900, 457.3331 |  |  | + | Lanost-7-en-18-oic acid, 16-(acetyloxy)-3-[[4-O-(6-deoxy-β-D-glucopyranosyl)-β-D-xylopyranosyl]oxy]-20-hydroxy-, γ-lactone, (3β,9β,16α)- (9CI)) |
| 314 ^n^ | 19.98 | 985.5323 | -H | C_50_H_82_O_19_ | -4.9 | -5 | 355.48  (985) | neg: 985.5323, 941.5444, 899.5334, 767.4946, 621.4367, 459.3839, 375.2890  pos: 1004.5781, 425.3794, 407.3687 | + |  |  | F-3+Glc+Rha+Ara+Mal |
| 315 ^n^ | 20.09 | 867.5082 | -H | C_46_H_76_O_15_ | -2.4 | -2.8 | 344.00  (913) | neg: 913.5109, 867.5082, 735.4686, 589.4114, 457.3642, 375.2932 | + | + | + | N-3+Ara+Rha+Xyl |
| 316 | 20.09 | 953.5079 | -H | C_49_H_78_O_18_ | -3.1 | -3.3 | 361.60  (999) | neg: 999.5142, 953.5079, 911.4986, 779.4582, 633.4008, 471.3476, 359.2949  pos: 455.3525, 437.3426 | + |  |  | Dammar-24-en-21-oic acid, 3-[(O-α-L-rhamnopyranosyl-(1→2)-O-[β-D-xylopy-ranosyl-(1→3)]-β-D-glucopyranosyl)oxy]-20,23-dihydroxy-, γ-lactone, (3β,23R) |
| 317 | 20.22 | 765.4380 | -H | C_41_H_66_O_13_ | -4.5 | -5.9 | 308.56  (811) | neg: 811.4453, 765.4380, 633.3997, 551.3615, 389.3059  pos: 784.4855, 453.3369, 435.3291 |  | + |  | Gypenbioside A |
| 318 ^a^ | 20.24 | 911.4959 | -H | C_47_H_76_O_17_ | -4.5 | -4.9 | 350.54  (957) | neg: 957.5004, 911.4959, 779.4564, 633.4005, 473.3629, 387.2538  pos: 930.5418, 453.3363, 435.3258, 417.3160, 407.3312 | + | + | + | GL-5 |
| 319 | 20.38 | 953.5085 | -H | C_49_H_78_O_18_ | -2.5 | -2.6 | 363.73  (999) | neg: 999.5115, 953.5085, 911.4982, 779.4578, 633.4009, 471.3477, 359.2953  pos: 455.3521, 437.342 | + |  |  | isomer of Dammar-24-en-21-oic acid, 3-[(O-α-L-rhamnopyranosyl-(1→2)-O-[β-D-xylopyranosyl-(1→3)]-β-D-glucopyran-osyl)oxy]-20,23-dihydroxy-, γ-lactone, (3β,23R)- (ACI) |
| 320 ^n^ | 20.41 | 997.4964 | -H | C_50_H_78_O_20_ | -4.4 | -4.4 | 360.34  (997) | neg: 997.4964, 953.5074, 911.4999, 779.4562, 633.4016, 471.3462, 359.2570 |  | + | + | C-1+Glc+Rha+Ara+Mal |
| 321 ^n^ | 20.43 | 779.4600 | -H | C_42_H_68_O_13_ | 1.8 | 2.3 | 320.55  (825) | neg: 825.4603, 779.4600, 751.4592, 667.4073, 521.3492, 389.3068  pos: 453.3365, 435.3262, 417.3173, 407.3329 |  |  | + | B-3+CH_3_+Ara+Rha |
| 322 | 20.54 | 953.5120 | -H | C_49_H_78_O_18_ | 1 | 1 | 361.30  (999) | neg: 999.5090, 953.5120, 911.4991, 779.4571, 633.4004, 471.3476, 389.3089 |  | + | + | isomer of Dammar-24-en-21-oic acid, 3-[(O-α-L-rhamnopyranosyl-(1→2)-O-[β-D-xylopyranosyl-(1→3)]-β-D-glucopyran-osyl)oxy]-20,23-dihydroxy-, γ-lactone, (3β,23R)- (ACI) |
| 323 ^n^ | 20.55 | 941.5450 | -H | C_49_H_82_O_17_ | -2.4 | -2.5 | 360.76  (987) | neg: 987.5476, 941.5450, 899.5336, 767.4932, 621.4367, 459.3838, 375.2900 | + |  |  | F-3+Glc+Rha+Ara+Ac |
| 324 ^n^ | 20.65 | 957.5391 | -H | C_49_H_82_O_18_ | -3.2 | -3.3 | 366.63  (1003) | neg: 1003.5420, 957.5391, 795.4861, 649.4337 | + |  |  | F-1+2Glc+Rha+CH_3_ |
| 325 | 20.68 | 953.5064 | -H | C_49_H_78_O_18_ | -4.6 | -4.8 | 362.73  (999) | neg: 999.5113, 953.5064, 911.4972, 779.4570, 633.4008, 471.3282, 359.2948  pos: 455.3523, 437.3432 | + | + |  | isomer of Dammar-24-en-21-oic acid, 3-[(O-α-L-rhamnopyranosyl-(1→2)-O-[β-D-xylopyranosyl-(1→3)]-β-D-glucopyran-osyl)oxy]-20,23-dihydroxy-, γ-lactone, (3β,23R)- (ACI) |
| 326 ^n^ | 20.73 | 935.4609 | -H | C_48_H_72_O_18_ | -3.1 | -3.3 | 323.19  (935) | neg: 935.4609, 849.4623, 717.4210, 571.3635, 439.3209 |  |  | + | R-1+Ara+Rha+Xyl+Mal-CH3 |
| 327 | 20.75 | 911.5264 | -H | C_48_H_80_O_16_ | 2.6 | 2.7 | 348.54  (957) | neg: 957.5449, 911.5264, 869.5256, 737.4836, 591.4269, 459.3854, 375.2849 |  |  | + | Hexopyranoside, (3β,16β)-16-hydroxy-3-(pentopyranosyloxy)dammar-24-en-20-yl 3-O-(3-O-acetylpentopyranosyl)-6-deoxy |
| 328 ^n^ | 20.79 | 1043.5367 | -H | C_52_H_84_O_21_ | -6 | -5.7 | 372.26  (1043) | neg: 1043.5367, 957.5368, 795.4886, 649.4338, 521.3755, 359.2963 | + |  |  | B-1+2Glc+Rha+CH_3_+Mal |
| 329 ^n^ | 20.87 | 923.4973 | -H | C_48_H_76_O_17_ | -3.1 | -3.4 | 349.85  (969) | neg: 969.5015, 923.4973, 881.4880, 749.4470, 603.3885, 471.3717, 389.3049, 359.2951 | + |  |  | C-1+Ara+Rha+Xyl+Ac |
| 330 | 21.01 | 953.5054 | -H | C_49_H_78_O_18_ | -5.6 | -5.9 | 352.55  (953) | neg: 953.5054, 909.5204, 867.5089, 735.4692, 589.4110, 425.3432, 389.3061 |  |  | + | isomer of Dammar-24-en-21-oic acid, 3-[(O-α-L-rhamnopyranosyl-(1→2)-O-[β-D-xylopyranosyl-(1→3)]-6-O-acetyl-β-D-glucopyranosyl)oxy]-20,23-dihydroxy-, γ-lactone, (3β,23S)- (9CI, ACI) |
| 331 ^n^ | 21.02 | 1041.5232 | -H | C_52_H_82_O_21_ | -3.8 | -3.6 | 369.46  (1041) | neg: 1041.5232, 997.5344, 955.5239, 823.4844, 781.4728, 697.4162, 551.3586, 389.3052 | + |  |  | B-1+Glc+Rha+Xyl+Mal+Ac |
| 332 ^n^ | 21.07 | 997.5344 | -H | C_51_H_82_O_19_ | -2.8 | -2.8 | 372.82  (1043) | neg: 1043.5367, 997.5344, 955.5239, 823.4844, 781.4728, 697.4162, 551.3586, 389.3052 | + |  |  | B-1+Glc+Rha+Xyl+2Ac |
| 333 ^n^ | 21.15 | 953.5063 | -H | C_49_H_78_O_18_ | -4.7 | -4.9 | 358.04  (999) | neg: 999.5096, 953.5063, 911.5397, 779.4557, 633.3992, 501.3599  pos: 453.3363, 435.3258 | + | + | + | B-3+Ara+Rha+Xyl+Ac+CH_3_ |
| 334 ^n^ | 21.21 | 1043.5363 | -H | C_52_H_84_O_21_ | -6.4 | -6.1 | 370.91  (1043) | neg: 1043.5363, 957.5383, 795.4877, 649.4318 | + | + |  | B-1+2Glc+Rha+CH_3_+Mal |
| 335 ^a^ | 21.30 | 925.5137 | -H | C_48_H_78_O_17_ | -2.4 | -2.6 | 357.31  (971) | neg: 971.5181, 925.5137, 883.5033, 751.4650, 667.4066, 521.3493, 389.3049 | + |  |  | GL-19 |
| 336 ^n^ | 21.32 | 997.4965 | -H | C_50_H_78_O_20_ | -4.3 | -4.3 | 358.83  (997) | neg: 997.4965, 953.5073, 911.4980, 779.4567, 633.3998, 501.3608, 473.3632  pos: 1016.5413, 453.3363, 435.3259 | + | + | + | B-3+CH_3_+Ara+Rha+Xyl+Mal |
| 337 ^n^ | 21.35 | 957.5377 | -H | C_49_H_82_O_18_ | -4.6 | -4.8 | 367.10  (1003) | neg: 1003.5420, 957.5377, 795.4877, 649.4315  pos: 976.5844, 439.3577, 421.3469 | + | + |  | F-1+2Glc+Rha+CH_3_ |
| 338 ^n^ | 21.36 | 1071.5337 | -H | C_53_H_84_O_22_ | -3.9 | -3.6 | 357.02  (1071) | neg: 1071.5337, 1027.5432, 985.5330, 853.4934, 779.4570, 633.4012  pos: 1090.5790, 455.3522, 437.3419, 419.1195 | + |  |  | B-1+OH+Ara+Rha+  Xyl+CH_3_+Mal+C_3_H_4_O_2_ |
| 339 ^n^ | 21.38 | 909.5169 | -H | C_48_H_78_O_16_ | -4.3 | -4.7 | 351.29  (955) | neg: 955.5206, 909.5169, 867.5121, 735.4693, 589.4100, 521.3499, 389.3064 |  | + | + | B-5−O+Ara+Rha+Xyl+Ac |
| 340 ^a^ | 21.52 | 953.5104 | -H | C_49_H_78_O_18_ | -0.6 | -0.6 | 360.60  (999) | neg: 999.5112, 953.5104, 911.4882, 779.4568, 633.4021, 471.3477, 359.2967 | + |  | + | GL-14 |
| 341 | 21.74 | 779.4572 | -H | C_42_H_68_O_13_ | -1 | -1.3 | 320.67  (825) | neg: 825.4606, 779.4572, 633.3997, 471.3450  pos: 453.3376, 435.326, 417.3261 |  |  | + | 3S,20S,23S-3,20,23-Trihydroxydammar-24-en-21-oic Acid-21,23-lactoneβ-D-glucopyranoside |
| 342 ^n^ | 21.76 | 1039.5065 | -H | C_52_H_80_O_21_ | -4.9 | -4.7 | 369.21  (1039) | neg: 1039.5065, 995.5187, 953.5091, 821.4675, 779.4575, 633.4005, 471.3477, 359.2944  pos: 1058.5546, 455.3526, 437.3421, 419.3318 | + | + | + | 3,20,23-trihydroxy dammar-24-en-21-oic acid 21,23-lactone-3-O-[4-O-acetyl-α-L-rhamnopyranosyl (1→2)]-[β-D-xylopyra-nosyl(1→3)]-6-O-malonyl-β-D-glucopyranoside |
| 343 ^n^ | 21.84 | 737.4845 | -H | C_41_H_70_O_11_ | 0.5 | 0.7 | 301.95  (783) | neg: 783.4873, 737.4845, 591.4280, 459.3870, 375.2905  pos: 425.3785, 407.3683 |  |  | + | F-3+Xyl+Rha |
| 344 | 21.88 | 913.5132 | -H | C_47_H_78_O_17_ | -2.9 | -3.2 | 353.45  (959) | neg: 959.5172, 913.5132, 781.4724, 635.4127, 473.3624  pos: 932.5574, 457.3687, 439.3572 | + | + | + | isomer of β-D-Glucopyranoside, (3β,20ξ)-21,23-epoxy-20,21-dihydroxydammar-24-en-3-yl O-α-L-rhamnopyranosyl-(1→2)-O-[β-D-xylopyranosyl-(1→3)]- (9CI, ACI) |
| 345 | 21.96 | 735.4688 | -H | C_41_H_68_O_11_ | 0.5 | 0.7 | 314.21  (781) | neg: 781.4717, 735.4688, 589.4124, 375.2870  pos: 441.3750, 423.3626, 405.3524 |  | + | + | Notoginsenoside ST12 |
| 346 ^n^ | 21.97 | 941.5443 | -H | C_49_H_82_O_17_ | -3.1 | -3.3 | 376.43  (987) | neg: 987.5477, 941.5443, 809.5032, 767.4932, 621.4366, 459.3839, 375.2887  pos: 960.5881, 425.3780, 407.3672 | + |  |  | F-3+Glc+Rha+Ara+Ac |
| 347 ^n^ | 21.99 | 1027.5436 | -H | C_52_H_84_O_20_ | -4.2 | -4.1 | 379.30  (1073) | neg: 1073.5474, 1027.5436, 985.5342, 853.4952, 779.4589, 633.3734, 503.3734 | + |  |  | B-1+OH+CH3+Ac+C_3_H_4_O_2_+2Xyl+Rha |
| 348 ^n^ | 22.03 | 999.5109 | -H | C_50_H_80_O_20_ | -5.6 | -5.6 | 349.44  (999) | neg: 999.5109, 955.5242, 913.5140, 781.4727, 635.4097, 473.3865, 389.3025 | + | + | + | B-1+Glc+Rha+Xyl+Mal |
| 349 | 22.07 | 927.5256 | -H | C_48_H_80_O_17_ | -6.1 | -6.6 | 356.59  (973) | neg: 973.5311, 927.5256, 765.4790, 619.4189, 473.3552 | + |  |  | isomer of Longipenoside GL3 |
| 350 ^a^ | 22.15 | 953.5092 | -H | C_49_H_78_O_18_ | -1.8 | -1.9 | 359.23  (999) | neg: 999.5115, 953.5092, 821.4680, 779.4564, 633.4017, 521.3482, 359.2944  pos: 972.5535, 455.3517, 437.3416 | + |  |  | GL-15 |
| 351 ^n^ | 22.17 | 1039.5065 | -H | C_52_H_80_O_21_ | -4.9 | -4.7 | 311.01  (1039) | neg: 1039.5065, 995.5190, 953.5081, 821.4677, 779.4577, 633.4006, 521.3481, 359.2951  pos: 1058.5538, 455.3523, 437.3418, 419.3328 | + |  |  | C-1+Glc+Rha+Xyl+Ac+Mal |
| 352 ^n^ | 22.24 | 909.5193 | -H | C_48_H_78_O_16_ | -1.9 | -2.1 | 353.75  (955) | neg: 955.5214, 909.5193, 867.5076, 735.4698, 589.4113, 473.3688 |  | + | + | B-5−O+Ara+Rha+Xyl+Ac |
| 353 | 22.42 | 927.5296 | -H | C_48_H_80_O_17_ | -2.1 | -2.3 | 357.82  (973) | neg: 973.5325, 927.5296, 795.4883, 649.4321, 583.4017  pos: 946.5735, 439.3575, 421.3575 | + | + | + | Dammar-24-en-21-al, 3-[(O-α-L-rhamno-pyranosyl-(1→2)-O-[β-D-glucopyranosyl-(1→3)]-α-L-arabinopyranosyl)oxy]-20,23-dihydroxy-, cyclic 21,23-(methyl acetal), (3β,20R,21S,23S)- (ACI) |
| 354 ^n^ | 22.55 | 985.5339 | -H | C_50_H_82_O_19_ | -3.3 | -3.3 | 366.29  (1031) | neg: 1031.5389, 985.5339, 853.4936, 707.4257, 503.3738  pos: 1004.5801, 455.3517, 437.3430 | + |  |  | B-1+Glc+Rha+Xyl+Ac+CH_3_+OH |
| 355 ^n^ | 22.61 | 1053.5228 | -H | C_53_H_82_O_21_ | -4.2 | -4 | 371.85  (1053) | neg: 1053.5228, 967.5237, 821.4681, 779.4575, 633.4011, 471.3495, 359.2958  pos: 455.3524, 437.3433 | + |  |  | C-1+Glc+2Rha+Mal+Ac |
| 356 ^n^ | 22.68 | 1071.5334 | -H | C_53_H_84_O_22_ | -4.2 | -3.9 | 374.32  (1071) | neg: 1071.5334, 1027.5453, 985.5339, 853.4942, 779.4579, 633.4005  pos: 1090.5797, 455.3525, 437.3428 | + |  |  | B-1+OH+Ara+Rha+  Xyl+CH_3_+Mal+C_3_H_4_O_2_ |
| 357 ^n^ | 22.68 | 825.4969 | +HCOO | C_43_H_72_O_12_ | -3.1 | -3.8 | 326.66  (825) | neg: 825.4969, 779.4559, 737.4845, 591.4286, 459.3828 |  |  | + | F-3+Xyl+Rha+CH_3_CO |
| 358 ^n^ | 22.70 | 1013.5266 | -H | C_51_H_82_O_20_ | -5.5 | -5.4 | 363.99  (1013) | neg: 1013.5266, 969.5384, 927.5279, 795.4880, 649.4311, 487.3776, 373.2732  pos: 1032.5737, 439.3575, 421.3470 | + | + | + | B-1+Glc+Rha+Xyl+Mal+CH3 |
| 359 | 22.74 | 927.5281 | -H | C_48_H_80_O_17_ | -3.6 | -3.9 | 359.67  (973) | neg: 973.5331, 927.5281, 795.4885, 649.4326  pos: 946.5735, 439.3574, 421.3466 | + | + | + | Dammar-24-en-21-al, 3-[(O-α-L-rham-nopyranosyl-(1→2)-O-[β-D-glucopyran-osyl-(1→3)]-α-L-arabinopyranosyl)oxy]-20,23-dihydroxy-, cyclic 21,23-(methyl acetal), (3β,20R,21S,23S)- (ACI) |
| 360 | 22.77 | 883.5020 | -H | C_46_H_76_O_16_ | -3.5 | -4 | 338.85  (929) | neg: 929.5060, 883.5020, 751.4614, 605.4044, 473.3827 | + | + | + | Isomer of Gylongiposide I |
| 361 ^n^ | 23.13 | 1043.5365 | -H | C_52_H_84_O_21_ | -3.1 | -3.1 | 372.88  (1043) | neg: 1043.5365, 957.4904, 795.4904, 649.4313 | + |  |  | B-1+2Glc+Rha+CH_3_+Mal |
| 362 | 23.24 | 999.5127 | +HCOO | C_49_H_78_O_18_ | -3.8 | -3.8 | 362.99  (999) | neg: 999.5127, 911.4955, 779.4575, 633.4014, 521.3476, 359.3164 | + |  |  | isomer of Dammar-24-en-21-oic acid, 3-[(O-α-L-rhamnopyranosyl-(1→2)-O-[β-D-xylopyranosyl-(1→3)]-β-D-glucopyran-osyl)oxy]-20,23-dihydroxy-, γ-lactone, (3β,23R)- (ACI) |
| 363 ^n^ | 23.28 | 823.4811 | +HCOO | C_43_H_70_O_12_ | -3.3 | -4 | 327.97  (823) | neg: 823.4811, 777.4717, 735.4671, 589.4091, 375.2905  pos: 423.3625 |  |  | + | N-3+Xyl+Rha+CH_3_CO |
| 364 ^n^ | 23.39 | 969.4999 | -H | C_49_H_78_O_19_ | -6 | -6.2 | 328.96  (969) | neg: 969.4999, 925.5122, 883.5019, 751.4614, 605.4066, 473.3627  pos: 988.5479, 457.3676, 439.3587 | + | + | + | B-1+Ara+Rha+Xyl+Mal |
| 365 | 23.39 | 995.5189 | -H | C_51_H_80_O_19_ | -2.7 | -2.7 | 370.22  (1041) | neg: 1041.5212, 995.5189, 953.5084, 821.4680, 779.4579, 633.4005, 471.3480, 359.2949  pos: 455.3521, 437.3419, 419.1180 | + |  |  | Dammar-24-en-21-oic acid, 3-[(O-4-O-acetyl-α-L-rhamnopyranosyl-(1→2)-O-[β-D-xylopyranosyl-(1→3)]-6-O-acetyl-β-D-glucopyranosyl)oxy]-20,23-dihydroxy-, γ-lactone, (3β,20R,23R)- (9CI, ACI) |
| 366 ^n^ | 23.59 | 983.5174 | -H | C_50_H_80_O_19_ | -4.2 | -4.3 | 362.09  (983) | neg: 983.5174, 939.5352, 897.5124, 765.4773, 619.4216, 473.3594 |  |  | + | F-1+2Rha+Xyl+Mal |
| 367 ^n^ | 23.62 | 985.5344 | -H | C_50_H_82_O_19_ | -2.8 | -2.8 | 370.65  (1031) | neg: 1031.5376, 985.5344, 853.4947, 811.4795, 665.3914, 503.3723  pos: 1004.5833, 455.3526, 437.3413 | + |  |  | B-1+Glc+Rha+Xyl+Ac+CH_3_+OH |
| 368 | 23.67 | 995.5187 | -H | C_51_H_80_O_19_ | -2.9 | -2.9 | 370.31  (1041) | neg: 1041.5212, 995.5187, 953.5080, 821.4672, 779.4571, 633.4001, 471.3476, 359.2943  pos: 455.3524, 437.3418, 419.1189 | + |  |  | Dammar-24-en-21-oic acid, 3-[(O-4-O-acetyl-α-L-rhamnopyranosyl-(1→2)-O-[β-D-xylopyranosyl-(1→3)]-6-O-acetyl-β-D-glucopyranosyl)oxy]-20,23-dihydroxy-, γ-lactone, (3β,20R,23R)- (9CI, ACI) |
| 369 ^n^ | 23.69 | 943.5219 | +HCOO | C_47_H_78_O_16_ | -4.7 | -5 | 350.06  (943) | neg: 943.5219, 897.5155, 765.4779, 619.4216  pos: 439.3576, 421.3467 | + | + | + | F-1+Ara+2Rha |
| 370 ^n^ | 23.77 | 1027.5424 | -H | C_52_H_84_O_20_ | -5.4 | -5.3 | 365.53  (1027) | neg: 1027.5424, 941.5444, 795.4874, 649.4316, 583.3983, 455.3533 | + |  |  | F-1+Glc+2Rha+CH_3_+Mal |
| 371 ^n^ | 23.83 | 1027.5439 | -H | C_52_H_84_O_20_ | -3.9 | -3.8 | 385.09  (1073) | neg: 1073.5490, 1027.5439, 985.5342, 853.4942, 779.4569, 633.3997, 503.3745 | + |  |  | B-1+OH+CH3+Ac+C_3_H_4_O_2_+2Xyl+Rha |
| 372 | 24.06 | 995.5172 | -H | C_51_H_80_O_19_ | -4.4 | -4.4 | 371.62  (1041) | neg: 1041.5212, 995.5172, 953.5068, 821.4675, 779.4568, 633.4003, 471.3469, 359.2945  pos: 1014.5643, 455.3521, 437.3418, 419.1190 | + |  |  | Dammar-24-en-21-oic acid, 3-[(O-4-O-acetyl-α-L-rhamnopyranosyl-(1→2)-O-[β-D-xylopyranosyl-(1→3)]-6-O-acetyl-β-D-glucopyranosyl)oxy]-20,23-dihydroxy-, γ-lactone, (3β,20R,23R)- (9CI, ACI) |
| 373 ^n^ | 24.13 | 983.5182 | -H | C_50_H_80_O_19_ | -3.4 | -3.5 | 359.86  (983) | neg: 983.5182, 939.5266, 897.5159, 765.4769, 619.4200, 473.3584  pos: 1002.5628, 439.3565, 421.3460 | + | + | + | F-1+2Rha+Xyl+Mal |
| 374 | 24.18 | 995.5187 | -H | C_51_H_80_O_19_ | -2.9 | -2.9 | 371.61  (1041) | neg: 1041.5220, 995.5187, 953.5074, 821.4680, 779.4578, 633.4007, 471.3495, 359.2943  pos: 455.3521, 437.3419, 419.1180 | + |  |  | Dammar-24-en-21-oic acid, 3-[(O-4-O-acetyl-α-L-rhamnopyranosyl-(1→2)-O-[β-D-xylopyranosyl-(1→3)]-6-O-acetyl-β-D-glucopyranosyl)oxy]-20,23-dihydroxy-, γ-lactone, (3β,20R,23R)- (9CI, ACI) |
| 375 ^n^ | 24.25 | 823.4810 | +HCOO | C_43_H_70_O_12_ | -3.4 | -4.1 | 327.77  (823) | neg: 823.4810, 735.680, 589.4097, 473.3754, 375.2905 |  |  | + | N-3+Xyl+Rha+CH_3_CO |
| 376 ^n^ | 24.31 | 969.5387 | -H | C_50_H_82_O_18_ | -3.6 | -3.7 | 368.64  (1015) | neg: 1015.5416, 969.5387, 927.5281, 795.4874, 649.4316, 583.4009, 455.3534  pos: 439.3573, 421.3465 | + | + |  | F-1+Glc+Rha+Xyl+CH_3_+Ac |
| 377 ^n^ | 24.33 | 943.5300 | +HCOO | C_47_H_78_O_16_ | 3.4 | 3.6 | 352.99  (943) | neg: 943.5300, 897.5142, 765.4754, 619.4187, 473.3643 |  |  | + | F-1+Ara+2Rha |
| 378 ^n^ | 24.33 | 969.5005 | -H | C_49_H_78_O_19_ | -5.4 | -5.6 | 320.28  (969) | neg: 969.5005, 925.5123, 883.5013, 751.4617, 605.4028, 473.3625  pos: 988.5479, 439.3596 |  | + | + | B-1+Ara+Rha+Xyl+Mal |
| 379 ^n^ | 24.37 | 925.5128 | -H | C_48_H_78_O_17_ | -3.3 | -3.6 | 339.78  (971) | neg: 971.5165, 925.5128, 883.5023, 751.4624, 667.4034, 521.3464, 389.3065 | + |  |  | F-1+Ara+Rha+Xyl+Ac |
| 380 ^n^ | 24.41 | 983.5168 | -H | C_50_H_80_O_19_ | -4.8 | -4.9 | 361.87  (983) | neg: 983.5168, 939.5271, 897.5182, 765.4771, 619.4218, 473.3699  pos: 1002.5663, 439.3579, 421.3466 | + | + | + | F-1+2Rha+Xyl+Mal |
| 381 ^n^ | 24.51 | 1015.5432 | +HCOO, -H | C_50_H_82_O_18_ | -4.6 | -4.5 | 370.06  (1015) | neg: 1015.5432, 969.5343, 927.5325, 795.4879, 649.4323, 583.3993, 455.3529  pos: 439.3583, 421.3475 | + |  |  | B-1+Glc+Rha+Xyl+CH_3_+Ac |
| 382 ^n^ | 24.55 | 951.4917 | -H | C_49_H_76_O_18_ | -3.6 | -3.8 | 348.09  (951) | neg: 951.4917, 865.4958, 733.4536, 587.3922, 455.3520 |  |  | + | E-2+Ara+Rha+Xyl+Mal−H_4_O |
| 383 ^n^ | 24.68 | 835.4836 | -H | C_45_H_72_O_14_ | -0.8 | -1 | 329.64  (881) | neg: 881.4855, 835.4836, 703.4417, 557.3859, 425.3418 |  |  | + | P-1−OH+Ara+Rha+Xyl |
| 384 | 24.75 | 737.4473 | -H | C_40_H_66_O_12_ | -0.3 | -0.4 | 286.75  (783) | neg: 783.4506, 737.4473, 605.4059, 473.3625 |  |  | + | Dammar-24-en-19-al, 20,21-dihydroxy-3-[(3-O-β-D-xylopyranosyl-α-L-arabinopyranosyl)oxy]-, (3β)- (ACI) |
| 385 | 24.76 | 999.5495 | -H | C_51_H_84_O_19_ | -3.4 | -3.4 | 372.24  (1045) | neg: 1045.5536, 999.5495, 867.5098, 765.4753, 619.4163, 457.3654 | + |  |  | (2α,3β,12β)-2,12-Dihydroxy-20-(β-D-xylopyranosyloxy)dammar-24-en-3-yl 2-O-[6-O-[(2E)-1-oxo-2-buten-1-yl]-β-D-glucopyranosyl]-β-D-glucopyranoside |
| 386 ^n^ | 24.81 | 969.5391 | -H | C_50_H_82_O_18_ | -3.2 | -3.3 | 368.21  (1015) | neg: 1015.5427, 969.5391, 927.5297, 795.4876, 649.4318, 583.3995, 455.3495  pos: 439.3572, 421.3468 | + | + |  | F-1+Glc+Rha+Xyl+CH_3_+Ac |
| 387 ^n^ | 24.98 | 925.5107 | -H | C_48_H_78_O_17_ | -5.4 | -5.8 | 343.94  (971) | neg: 971.5152, 925.5107, 883.5010, 751.4631, 605.4062, 473.3611  pos: 439.3611 | + | + | + | F-1+Ara+Rha+Xyl+Ac |
| 388 ^n^ | 25.26 | 939.5286 | -H | C_49_H_80_O_17_ | -3.1 | -3.3 | 359.88  (985) | neg: 985.5295, 939.5286, 897.5184, 765.4772, 619.4217 | + | + | + | N-3+Glc+Rha+Xyl+Ac |
| 389 ^n^ | 25.29 | 983.5186 | -H | C_50_H_80_O_19_ | -3 | -3.1 | 349.91  (983) | neg: 983.5186, 939.5286, 897.5184, 765.4772, 619.4217  pos: 1002.5625, 439.3575, 421.3471 |  | + |  | F-1+2Rha+Xyl+Mal |
| 390 | 25.38 | 995.5172 | -H | C_51_H_80_O_19_ | -4.4 | -4.4 | 369.75  (1041) | neg: 1041.5220, 995.5172, 953.5073, 821.4664, 779.4561, 633.3998, 471.3473, 359.2945  pos: 1014.5627, 455.3519, 437.3420 | + |  |  | Dammar-24-en-21-oic acid, 3-[(O-4-O-acetyl-α-L-rhamnopyranosyl-(1→2)-O-[β-D-xylopyranosyl-(1→3)]-6-O-acetyl-β-D-glucopyranosyl)oxy]-20,23-dihydroxy-, γ-lactone, (3β,20R,23R)- (9CI, ACI) |
| 391 ^n^ | 25.80 | 1015.5442 | +HCOO | C_50_H_82_O_18_ | -3.5 | -3.6 | 368.21  (1015) | neg: 1015.5442, 969.5368, 927.5286, 795.4885, 649.4321, 583.4009, 455.3535  pos: 988.5836, 439.3574, 421.3469 | + |  |  | B-1+Glc+Rha+Xyl+CH_3_+Ac |
| 392 ^n^ | 25.81 | 939.5276 | -H | C_49_H_80_O_17_ | -4.1 | -4.4 | 362.76  (985) | neg: 985.5305, 939.5276, 897.5126, 765.4780, 619.4214 |  | + | + | N-3+Glc+Rha+Xyl+Ac |
| 393 ^n^ | 25.97 | 1055.5380 | -H | C_53_H_84_O_21_ | -4.7 | -4.5 | 374.63  (1055) | neg: 1055.5380, 1011.5461, 969.5394, 837.5011, 795.4874, 649.4282  pos: 1074.5848, 439.3593, 421.3476 | + |  |  | F-1+Ara+Glc+Rha+CH_3_+Ac+Mal |
| 394 | 26.15 | 995.5200 | -H | C_51_H_80_O_19_ | -1.6 | -1.6 | 372.00  (1041) | neg: 1041.5220, 995.5200, 953.5085, 821.4663, 779.4554, 633.4005, 359.2941  pos: 1014.5635, 455.3524, 437.3423 | + |  |  | Dammar-24-en-21-oic acid, 3-[(O-4-O-acetyl-α-L-rhamnopyranosyl-(1→2)-O-[β-D-xylopyranosyl-(1→3)]-6-O-acetyl-β-D-glucopyranosyl)oxy]-20,23-dihydroxy-, γ-lactone, (3β,20R,23R)- (9CI, ACI) |
| 395 ^n^ | 26.20 | 1055.5385 | -H | C_53_H_84_O_21_ | -4.2 | -4 | 374.88  (1055) | neg: 1055.5385, 1011.5494, 969.5393, 837.4979, 795.4870, 649.4305  pos: 1074.5840, 439.3575, 421.3467 | + |  |  | F-1+Ara+Glc+Rha+CH_3_+Ac+Mal |
| 396 ^n^ | 26.25 | 995.5200 | -H | C_50_H_82_O_18_ | -3.3 | -3.4 | 367.04  (1015) | neg: 1015.5376, 969.5390, 837.4975, 795.4875, 649.4304, 583.3985, 455.3538 | + | + |  | B-1+Glc+Rha+Xyl+CH_3_+Ac |

a: reference standards, n: new compounds
